# Optimizing multifunctional fluorescent ligands for intracellular labeling

**DOI:** 10.1073/pnas.2510046122

**Published:** 2025-10-27

**Authors:** Pratik Kumar, Jason D. Vevea, Ariana N. Tkachuk, Kirby R. Campbell, Emma T. Watson, Anthony X. Ayala, Jonathan B. Grimm, Edwin R. Chapman, David J. Solecki, Luke D. Lavis

**Affiliations:** ^a^Janelia Research Campus, HHMI, Ashburn, VA 20147; ^b^Neuronal Cell Biology Division, Department of Developmental Neurobiology, St. Jude Children’s Research Hospital, Memphis, TN 38104; ^c^Department of Neuroscience, University of Wisconsin–Madison, Madison, WI 53705; ^d^HHMI, University of Wisconsin–Madison, Madison, WI 53705

**Keywords:** chemical biology, chemistry, fluorescence, protein purification, HaloTag

## Abstract

Enzyme-based self-labeling tags enable the covalent attachment of synthetic molecules to proteins inside living cells. A frontier of this field is designing cell-permeable multifunctional ligands that contain fluorophores in combination with affinity tags or pharmacological agents. This is challenging since attachment of additional chemical moieties onto fluorescent ligands can adversely affect membrane permeability. To address this problem, we examined the chemical properties of rhodamine-based self-labeling tag ligands through the lens of medicinal chemistry. We found that the lactone–zwitterion equilibrium constant (*K*_L–Z_) of rhodamines inversely correlates with their distribution coefficients (log*D*_7.4_), suggesting that ligands based on dyes exhibiting low *K*_L–Z_ and high log*D*_7.4_ values, such as Si-rhodamines, would efficiently enter cells. We designed cell-permeable multifunctional HaloTag ligands with a biotin moiety to purify mitochondria or a JQ1 appendage to translocate BRD4 within the nucleus. We found that translocation of BRD4 to constitutive heterochromatin in cells leads to apparent increases in transcriptional activity. These fluorescent reagents enable affinity capture and translocation of intracellular proteins in living cells, and our general design concepts will facilitate the design of multifunctional chemical tools for biology.

## Significance

Understanding cellular processes requires tools to measure and manipulate biomolecules in living systems. Self-labeling tags, such as the HaloTag, enable the attachment of synthetic molecules to specific proteins inside cells. Creating ligands for these systems with more than one chemical motif remains challenging due to competing demands between cell permeability and functionality. We found that multifunctional ligands based on certain rhodamines efficiently entered cells and enabled affinity purification of mitochondria or translocation of nuclear proteins; the performance of these molecules could be verified by fluorescence microscopy. These compounds are useful for a variety of biological experiments, and our general framework will allow the design of other multifunctional ligands to study living systems.

**Fig. 1.**
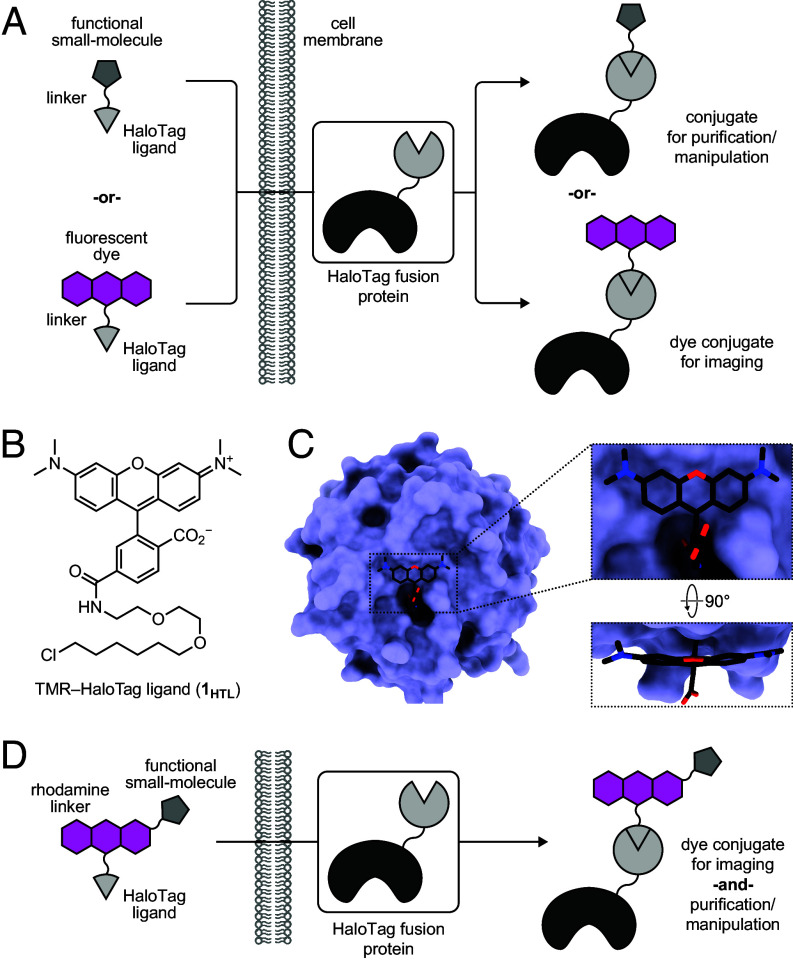
Rhodamine-based multifunctional ligands for self-labeling tags. (*A*) Schematic illustrating the modularity of self-labeling tag systems for a singular function. (*B*) Chemical structure of **1**_**HTL**_. (*C*) Crystal structure of **1**_**HTL**_ covalently bound to the HaloTag (PDB:6U32) with zoom-in of the dye–protein interface. (*D*) Schematic showing the concept of multifunctional ligands.

Research at the interface of organic chemistry and protein biochemistry has generated powerful tools to visualize, purify, and manipulate cellular components (1–3). Introducing synthetic moieties into cells can be accomplished in various ways. Metabolic incorporation (4, 5) utilizes endogenous enzymes to install nonnative moieties into cells while genetic code expansion, (6, 7) engineered ligases, (8–12) or self-labeling tags (13–15) utilize exogenously expressed enzymes. In particular, engineering of enzyme–substrate interactions has produced self-labeling tags such as HaloTag (13) and SNAP-tag (14), which have found broad use in modern biology. These protein tags react specifically and irreversibly with a ligand motif that can be appended to a variety of functionalities, including fluorescent dyes, affinity tags, and pharmacological agents (Fig. 1*A*) (16–21).

Despite the prospective flexibility of self-labeling tag systems, the major application of this technology has been to label proteins with small-molecule fluorophores. These systems complement fluorescent proteins, and their original engineering focused on dye labeling. For example, the directed evolution campaign to generate the HaloTag used tetramethylrhodamine (TMR) linked to a 1-chlorohexane ligand at the 6-position via a short polyethylene glycol (PEG) unit (13). Reaction of the TMR–HaloTag ligand (**1_HTL_**, Fig. 1*B*) with the HaloTag protein positions TMR close to the HaloTag surface (Fig. 1*C*; PDB:6U32) (22). This general design of the PEG_2_-chlorohexane moiety (*i.e.*, the HaloTag ligand) attached to 6-carboxyrhodamines has generated a portfolio of spectrally distinct fluorescent HaloTag ligands that can function in cells, tissues, and animals (16, 22–26).

The intimate dye–protein contact exemplified by the **1_HTL_**–HaloTag complex (Fig. 1*C*) can be advantageous for fluorescent ligands. The tight association can shift some dyes from nonfluorescent forms in solution to fluorescent conjugates, yielding high-contrast fluorogenic systems (26–30). Association with the HaloTag can also improve brightness and photostability (31). Nevertheless, the relatively short PEG_2_-chloroalkane substrate motif can be problematic when the rhodamine moiety is replaced with other functionalities, such as affinity tags or pharmacological agents. Given this issue, an emerging idea in the field is to retain the dye and append it with motifs for purification or perturbation, creating multifunctional ligands (Fig. 1*D*). This takes advantage of the rapid labeling kinetics of the HaloTag system (~10^7^ M^−1^s^−1^), even when functionality is added to the dye moiety, (13, 21, 32) and improvements in chemistry (33–36) that allow the straightforward synthesis of functionalized fluorophores (37–43). Incorporation of a fluorescent dye also enables visualization of the subcellular distribution of ligands inside cells to verify their performance using fluorescence imaging.

Multifunctional fluorescent ligands can be relatively large molecules that show variable cell permeability since they combine ligand, dye, and affinity tag or pharmacological agent into a single compound. We set out to elucidate general design principles for such molecules, exploring how the chemical properties of the parent rhodamine structure (27–30, 36, 44) affect the permeability of multifunctional ligands built from that dye. We found that the lactone–zwitterion equilibrium constant (*K*_L–Z_) of rhodamine dyes is inversely correlated with their octanol–water distribution coefficient at physiological pH (log*D*_7.4_), a property commonly used in medicinal chemistry (45–49). Although the addition of functionality onto the dye structure affects *K*_L–Z_ and log*D*_7.4_, the changes are relatively minor; the rhodamine scaffold exerts a strong effect on the resulting multifunctional ligand properties. Dyes with relatively low *K*_L–Z_ values and high log*D*_7.4_, such as the Si-rhodamine-based Janelia Fluor 646 (JF_646_) and JF_635_, were particularly useful scaffolds for constructing cell-permeable compounds bearing the polar affinity tag biotin or the lipophilic pharmacological agent JQ1. These ligands enable advanced cell biological experiments, and the general concepts described below should aid the design of new multifunctional chemical tools for biology.

## Results and Discussion

### *K*_L–Z_ and log*D*_7.4_ Are Correlated.

**Fig. 2.**
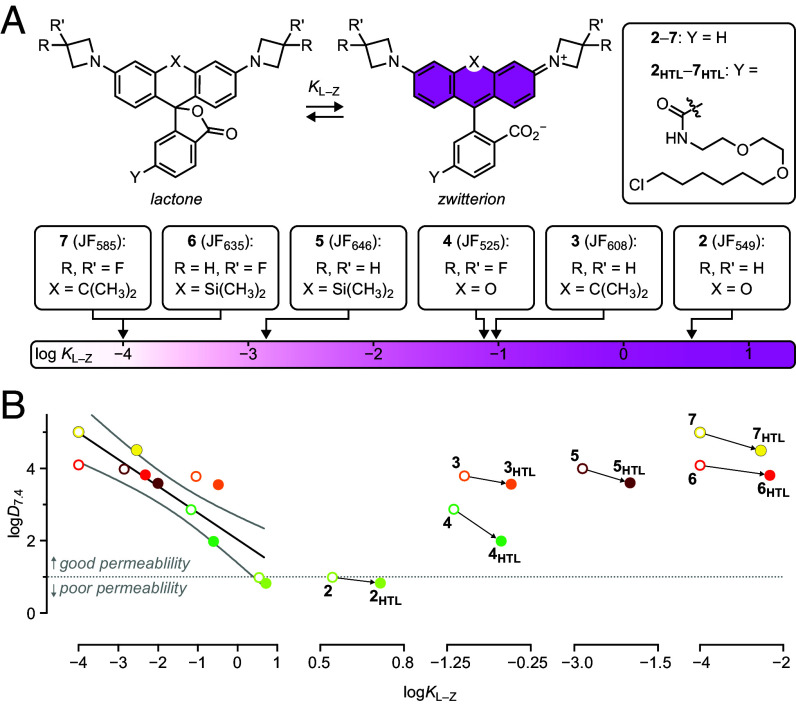
Relationship between *K*_L–Z_ and logD_7.4_ for rhodamines **2**–**7** and ligands **2**_**HTL**_–**7**_**HTL**_. (*A*) Dynamic equilibrium between the nonfluorescent lactone (L) and fluorescent zwitterion (Z) along with the lactone–zwitterion equilibrium constant (*K*_L–Z_; log scale) for rhodamine-based Janelia Fluor (JF) dyes **2**–**7** and their HaloTag ligands (**2**_**HTL**_–**7**_**HTL**_). (*B*) Plots of log*D*_7.4_ *vs.* log*K*_L–Z_ with linear fit (black line) and 95% CI (gray curves) for **2**–**7** and **2**_**HTL**_–**7**_**HTL**_; the dotted line denotes the region of optimal cellular permeability based on log*D*_7.4_. Individual pairs of free dyes and their respective HaloTag ligands show the consistent shift to lower log*D*_7.4_ and higher *K*_L–Z_ values upon installation of the HaloTag ligand moiety.

In previous work, we showed that the *K*_L–Z_ of the parent rhodamine (Fig. 2*A*) exerts a strong effect on the performance of dye-ligands in biological systems (36). Dyes with high *K*_L–Z_ values, such as Janelia Fluor 549 (JF_549_, **2**; *K*_L–Z_ = 3.5), predominantly exist in the zwitterion form to yield bright and environmentally insensitive ligands. JF_549_ is structurally similar to TMR, the base dye of **1_HTL_**, but contains azetidines instead of *N,N*-dimethylamino groups, which increase brightness and photostability (50). The *K*_L–Z_ can be tuned lower by replacing the xanthene oxygen in **2** with a *gem*-dimethylcarbon (e.g., JF_608_, **3**; *K*_L–Z_ = 0.091) or by installing 3,3-difluoroazetidine auxochromes, as in JF_525_ (**4**; *K*_L–Z_ = 0.068). Carborhodamine **3** exhibits a 60 nm bathochromic shift in absorption maximum (*λ*_abs_) and fluorescence emission maximum (*λ*_em_) relative to JF_549_ (**2**), whereas the 3,3-difluoroazetidine groups in **4** cause a 24-nm hypsochromic shift in *λ*_abs_ and *λ*_em_ (21). Ligands based on JF_608_ or JF_525_ exhibit improved membrane permeability and bioavailability in animals (27). Dyes with even lower *K*_L–Z_ values can be accessed by incorporating a *gem*-dimethylsilicon substituent (e.g., JF_646_, **5**; *K*_L–Z_ = 0.0014), which results in a larger 100-nm bathochromic shift in *λ*_abs_ and *λ*_em_, or by combining silicon or carbon substitutions with fluorinated azetidine auxochromes, as in JF_635_ (**6**; *K*_L–Z_ ≈ 0.0001) and JF_585_ (**7**; *K*_L–Z_ ≈ 0.001) (51–53). Compounds based on **5**–**7** show high cell permeability and are fluorogenic, predominantly existing in the lactone form in aqueous media but shifting to the fluorescent zwitterion upon binding their cognate biomolecular target (27).

We investigated whether the *K*_L–Z_ was correlated to log*D*_7.4_, which is a metric employed in medicinal chemistry to rationalize the permeability of small-molecule pharmacological agents (46, 48). This parameter has not been applied to self-labeling tag ligands, however, so the *K*_L–Z_ values of the HaloTag ligands (**2_HTL_**–**7_HTL_**; Fig. 2*A*) (27, 36) and the log*D*_7.4_ values for **2**–**7** and **2_HTL_**–**7_HTL_** were measured (54). These data revealed that log*D*_7.4_ is inversely correlated to *K*_L–Z_ (Fig. 2*B*). Although the relationship between log*D*_7.4_ and cell permeability is complex, cellular entry is optimal when log*D*_7.4_ is greater than 1 but less than 3–5; (45–49) the permeability of higher molecular weight molecules benefits from log*D*_7.4_ values at the upper end of this range (46). Based on these general rules, we hypothesized that multifunctional ligands based on JF_608_ (**3**; log*D*_7.4_ = 3.78), JF_646_ (**5**; log*D*_7.4_ = 3.98), and JF_635_ (**6**; log*D*_7.4_ = 4.10) could serve as effective scaffolds for cell-permeable compounds. We also surmised that the utility of JF_549_ (**2**) in multifunctional ligands could be limited due to its lower log*D*_7.4_ = 0.98, which is at the edge of the optimal range. Although JF_525_ (**4**) and JF_585_ (**7**) also exhibit potentially useful log*D*_7.4_ values—2.86 and 5.01, respectively—the 3,3-difluoroazetidine moiety in these compounds prevents attachment of functional small molecules using the expedient (39) azetidine derivatization; these dyes were not investigated further as scaffolds for multifunctional ligands.

### Synthesis and Properties of Biotin–JF–HaloTag Ligands.

**Scheme 1.**
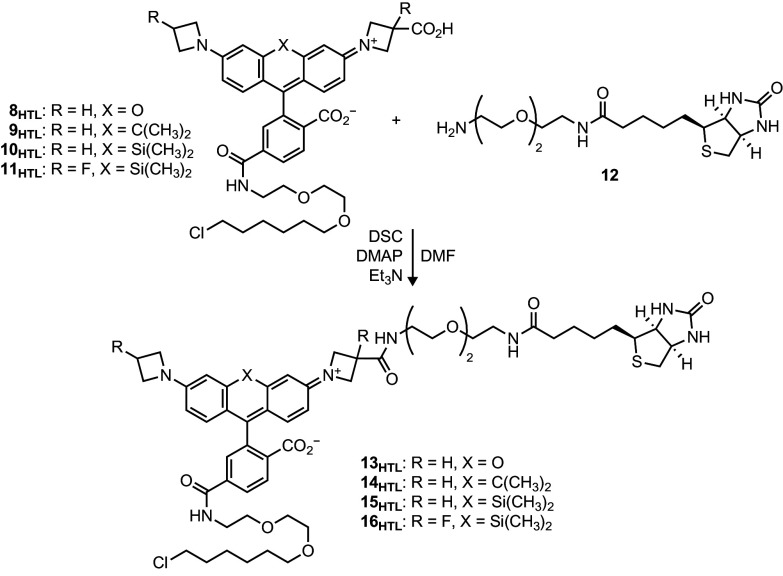
Synthesis of biotin–JF–HaloTag ligands **13**_**HTL**_–**16**_**HTL**_ from 3″-carboxyazetidine dyes **8**_**HTL**_–**11**_**HTL**_ and biotin–amine **12**.

We prepared a series of HaloTag ligands containing biotin (55) to test how different rhodamine scaffolds affect the properties of the resulting multifunctional compound. Coupling 3-carboxyazetidine-containing rhodamine HaloTag ligands **8_HTL_**–**11_HTL_** with the commercially available biotin–PEG_2_–NH_2_ (**12**) yielded the biotin–JF–HaloTag ligand compounds **13_HTL_**–**16_HTL_** (Scheme 1). The 3″-carboxy-JF_549_–HaloTag ligand (**8_HTL_**) and the 3″-carboxy-JF_646_–HaloTag ligand (**10_HTL_**) starting materials were prepared as previously described (39). The JF_608_ and JF_635_ derivatives **9_HTL_** and **11_HTL_** were synthesized using analogous five-step sequences (*SI Appendix*, Schemes S1 and S2).

**Fig. 3.**
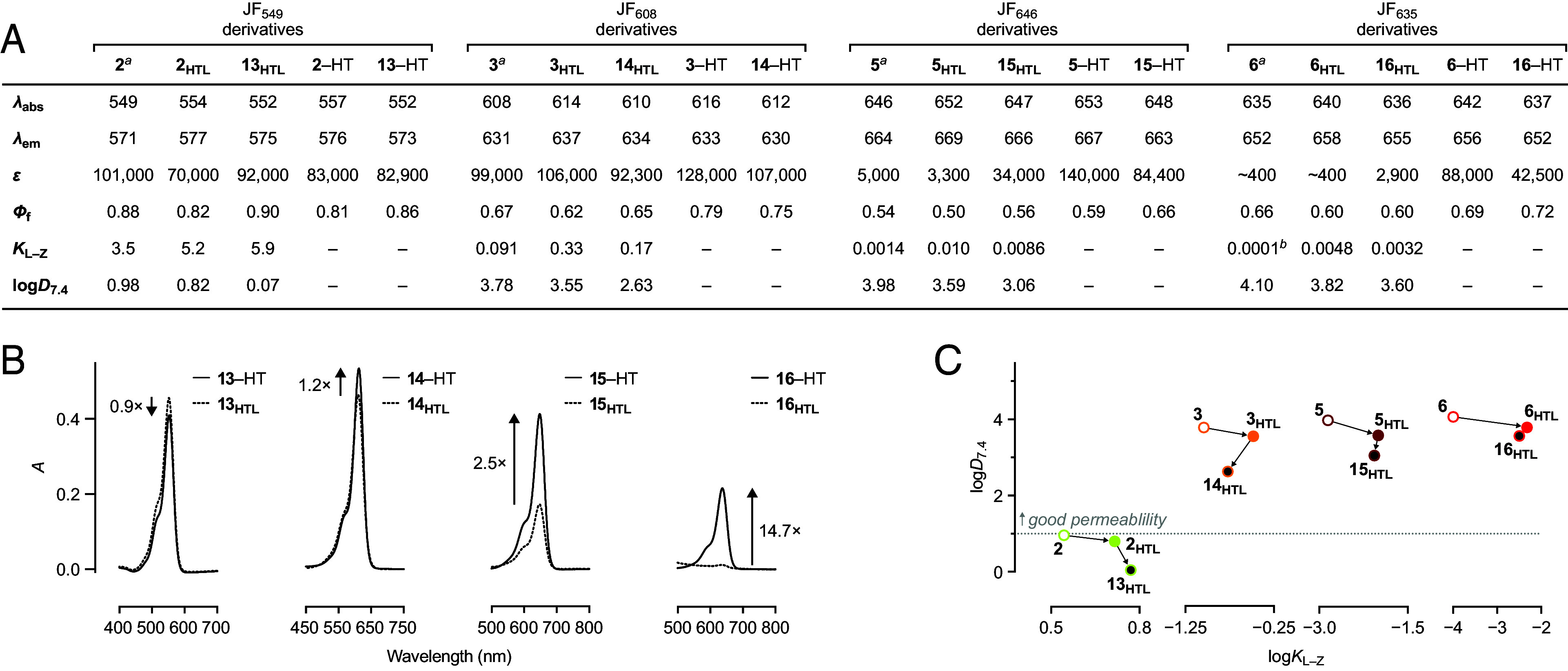
Spectral and chemical properties of biotin–rhodamine–HaloTag ligands. (*A*) Spectral properties (*λ*_abs_, *λ*_em_, *ε*, and *Φ*_f_), *K*_L–Z_, and log*D_7_*_.4_ of rhodamines **2**–**6**, HaloTag ligands **2**_**HTL**_–**6**_**HTL**_, biotin–rhodamine–HaloTag ligands **13**_**HTL**_–**16**_**HT**L_, and the HaloTag (HT) conjugates of **2**_**HTL**_–**6**_**HTL**_ and **13**_**HTL**_–**16**_**HTL**_; *λ*_abs_/*λ*_em_ are in nm, and *ε* are in M^−1^cm^−1^. Spectral properties were measured in 10 mM HEPES, pH 7.3, and *K*_L–Z_ measurements were performed in 1:1 (v/v) dioxane–water. *^a^*Data taken from refs. 27 and 50. *^b^*Data taken from ref. 27 (*B*) Absolute absorbance of **13**_**HTL**_–**16**_**HTL**_ in the absence or presence of excess (+HT) HaloTag protein. (*C*) Plots of log*D*_7.4_ *vs.* log*K*_L–Z_ for compounds based on JF_549_ (**2**, **2**_**HTL**_, and **13**_**HTL**_), JF_608_ (**3**, **3**_**HTL**_, and **14**_**HTL**_), JF_646_ (**5**, **5**_**HTL**_, and **15**_**HTL**_), and JF_635_ (**6**, **6**_**HTL**_, and **16**_**HTL**_) showing the consistent shift to lower log*D*_7.4_ values upon installation of the biotin moiety; the dotted line denotes region of optimal cellular permeability based on log*D*_7.4_.

The spectral properties of the biotin–JF–HaloTag ligands **13_HTL_**–**16_HTL_** and their HaloTag conjugates were similar to the parent dyes **2**–**6** and HaloTag ligands **2_HTL_**–**6_HTL_**(27, 50) with only minor shifts in *λ*_abs_ and *λ*_em_, regardless of chemical substitution or attachment to the HaloTag protein (Fig. 3*A*). The fluorescence quantum yield (*Φ*_f_) showed little change for the JF_549_ compounds upon HaloTag labeling but modestly increased (~20%) for the other compounds built on JF_608_, JF_646_, and JF_635_. The compounds based on JF_549_ (**2_HTL_** and **13_HTL_**) and JF_608_ (**3_HTL_** and **14_HTL_**) exhibited relatively high extinction coefficients (*ε*) before and after conjugation to the HaloTag, but the *ε* of the Si-rhodamine-based JF_646_–HaloTag ligands (**5** and **15_HTL_**) and JF_635_–HaloTag ligands (**6** and **16_HTL_**) were strongly affected by protein binding (Fig. 3 *A* and *B*). The absorptivity of biotin–JF_646_–HaloTag ligand (**15_HTL_**; *ε* = 34,000 M^−1^cm^−1^) increased to *ε* = 84,400 M^−1^cm^−1^ as the HaloTag conjugate **15**–HT. The biotin–JF_635_–HaloTag ligand (**16_HTL_**) exhibits a lower extinction coefficient (*ε* = 2,900 M^−1^cm^−1^) due to the fluorine-induced shift in the lactone–zwitterion equilibrium, but upon binding to HaloTag, the absorptivity increased; conjugate **16**–HT showed *ε* = 42,500 M^−1^cm^−1^. These HaloTag-induced increases in *ε* corresponded to fluorogenicities of 2.9-fold and 17.6-fold for **15_HTL_** and **16_HTL_**, respectively, driven primarily by the increase in absorptivity (Fig. 3*B*).

Measuring the *K*_L–Z_ and log*D*_7.4_ values of HaloTag ligands **13_HTL_**–**16_HTL_** revealed the effect of biotin attachment on chemical properties. The JF_549_-based **13_HTL_** exhibited a *K*_L–Z_ = 5.9, which is higher than both JF_549_ (**2**; *K*_L–Z_ = 3.5) and JF_549_–HaloTag ligand (**2_HTL_**; *K*_L–Z_ = 5.2), suggesting the polar biotin functionality stabilizes the zwitterion form of the rhodamine, perhaps through direct interaction with the dye. This effect was also observed in the decreased log*D*_7.4_ = 0.07 measured for **13_HTL_**, which places it below the optimal range for cell-permeant molecules (45–49). For ligands **14_HTL_**–**16_HTL_**, the incorporation of the biotin moiety caused a small decrease in *K*_L–Z_ compared to the unfunctionalized HaloTag ligands **3_HTL_**–**6_HTL_**, likely due to the electron-withdrawing carboxamide on the azetidine substituent. The lower *K*_L–Z_ values are reflected in the relatively high log*D*_7.4_ values measured for **14_HTL_** (log*D*_7.4_ = 2.63), **15_HTL_** (log*D*_7.4_ = 3.06), or **16_HTL_** (log*D*_7.4_ = 3.60). Overall, we found that incorporating biotin into rhodamine–HaloTag ligands has modest effects on *K*_L–Z_ and decreases the log*D*_7.4_ (Fig. 3*C*). This decrease in log*D*_7.4_ is substantial for the JF_549_-based **13_HTL_** but attenuated for compounds containing carbo- or Si-rhodamines (**14_HTL_**–**16_HTL_**).

### Live Cell Imaging with Multifunctional Ligands.

**Fig. 4.**
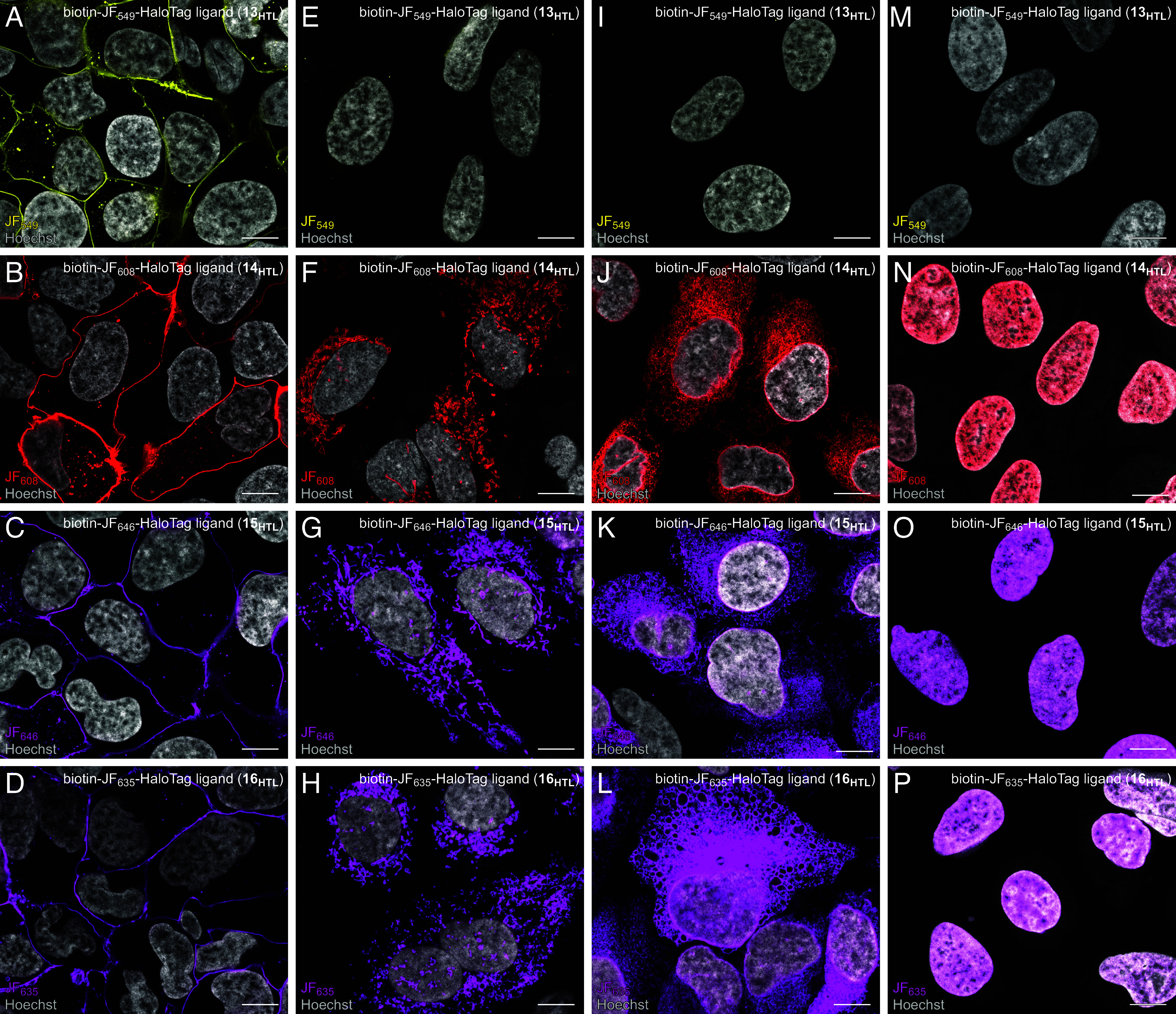
Evaluation of live-cell labeling of biotin–rhodamine–HaloTag ligands **13**_**HTL**_–**16**_**HTL**_. Airyscan fluorescence microscopy images of U2OS cells expressing HaloTag fusion proteins at different subcellular locations, incubated with 100 nM **13**_**HTL**_–**16**_**HTL**_ for 1 h followed by fixation, counterstaining with Hoechst 33342 (gray) and imaging. Columns indicate different HaloTag fusion proteins: (*A*–*D*) Cell-surface-localized HaloTag–PDGFR; (*E*–*H*) Outer mitochondrial membrane-localized HaloTag–TOMM20; (*I*–*L*) Endoplasmic reticulum membrane-localized HaloTag–Sec61β; (*M*–*P*) Nucleus-localized HaloTag–histone H2B. Rows indicate different ligands: (*A*,*E*,*I*, and *M*) biotin–JF_549_–HaloTag ligand (**13**_**HTL**_; yellow); (*B*, *F*, *J*, and *N*) biotin–JF_608_–HaloTag ligand (**14**_**HTL**_; red); (*C*, *G*, *K*, and *O*) biotin–JF_646_–HaloTag ligand (**15**_**HTL**_; magenta); (*D*, *H*, *L*, and *P*) biotin–JF_635_–HaloTag ligand (**16**_**HTL**_; magenta). Each ligand image set (*A*/*E*/*I*/*M*, *B*/*F*/*J*/*N*, *C*/*G*/*K*/*O*, or *D*/*H*/*L*/*P*) was taken using the same microscope settings and is displayed with the same dynamic range settings; (Scale bar, 10 μm.)

Based on these in vitro log*D*_7.4_ measurements, we anticipated that the JF_549_ compound (**13_HTL_**; log*D*_7.4_ = 0.07) would exhibit poor cell permeability, while other compounds **14_HTL_**–**16_HTL_** (log*D*_7.4_ = 2.63 to 3.60) would readily enter cells. We evaluated the biotin ligands in U2OS cells transiently transfected with plasmids encoding HaloTag fused to the following proteins: i) cell-surface-localized platelet-derived growth factor receptor (PDGFR); ii) outer mitochondria membrane localized TOMM20; iii) endoplasmic reticulum-localized Sec61β; and iv) nucleus-localized histone H2B. Evaluating the ligands with HaloTag fusions at different subcellular locations was important, as some rhodamine dyes inherently localize to specific regions (35, 56, 57). Carborhodamine and Si-rhodamine-based biotin ligands **14_HTL_**–**16_HTL_** exhibited bright and robust labeling of all the HaloTag fusions regardless of subcellular location, whereas the biotin–JF_549_–HaloTag ligand (**13_HTL_**) demonstrated low intracellular signals, only labeling the cell-surface-localized PDGFR–HaloTag fusion (Fig. 4). We note that **13_HTL_** has a similar design to the recently reported biotin–TMR–HaloTag ligand, whose in vitro labeling kinetics approached those of the unmodified TMR–HaloTag ligand (21) (**1_HTL_**); the biotin–TMR compound has not been evaluated in living cells. Measuring the loading kinetics in U2OS cells expressing HaloTag–H2B confirmed negligible intracellular labeling over 4 h using rhodamine ligand **13_HTL_** (200 nM), whereas the carborhodamine ligand (**14_HTL_**) and Si-rhodamine ligands (**15_HTL_** and **16_HTL_**) label intracellular proteins, reaching maximal intensity at 4 h and 1 h, respectively (*SI Appendix*, Fig. S1). These data affirm that log*D*_7.4_ is predictive of HaloTag ligand performance in cells and that low *K*_L–Z_ dyes can balance the addition of polar moieties and preserve cell permeability.

### Affinity Purification Using Biotin–Rhodamine–HaloTag Ligands.

**Fig. 5.**
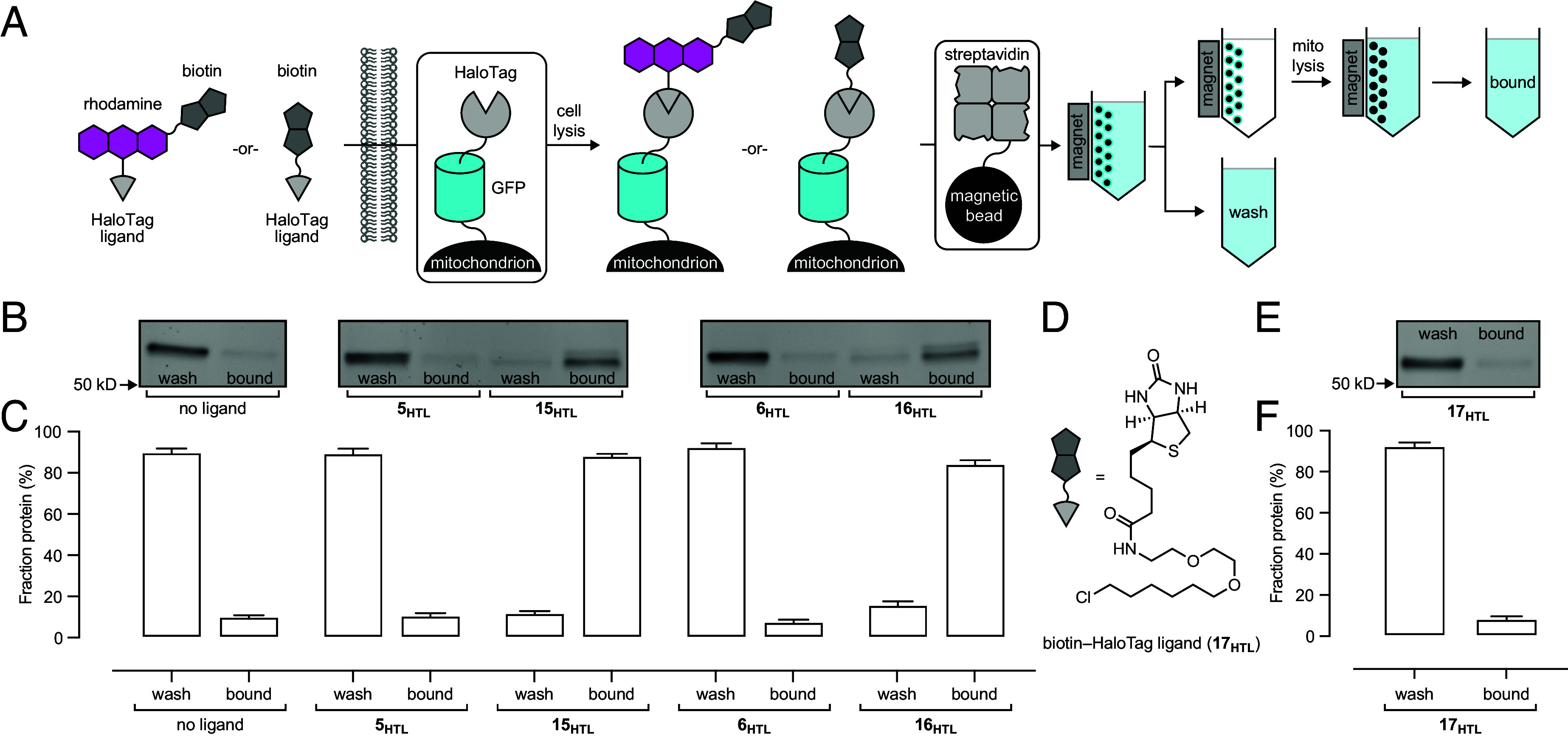
Affinity purification of mitochondria using biotin-containing multifunctional HaloTag ligands. (*A*) Schematic illustrating the workflow to purify mitochondria using biotin-containing HaloTag ligands by live-cell labeling and affinity purification using streptavidin-coated magnetic beads. (*B* and *C*) Representative GFP SDS–PAGE/in-gel fluorescence (*B*) and quantification (*C*) of OMP25–msGFP–HaloTag fusion protein in the wash and bound to streptavidin after labeling with vehicle (DMSO-only), JF_646_–HaloTag ligand (**5**_**HTL**_), biotin–JF_646_–HaloTag ligand (**15**_**HTL**_), JF_635_–HaloTag ligand (**6**_**HTL**_), and biotin–JF_635_–HaloTag ligand (**16**_**HTL**_); *n* = 3, error bars indicate mean ± SEM. (*D*) Schematic and chemical structure of commercial biotin–HaloTag ligand **17**_**HTL**_. (*E* and *F*) Representative GFP SDS–PAGE/in-gel fluorescence (*E*) and quantification (*F*) of captured OMP25–msGFP–HaloTag fusion protein in the wash and bound to streptavidin after labeling with **17**_**HTL**_; *n* = 3, error bars indicate mean ± SEM.

We applied the biotin-containing multifunctional ligands **13_HTL_**–**16_HTL_** for affinity purification of mitochondria from HEK293T cells expressing a fusion protein consisting of outer membrane protein 25 (OMP25), monomeric superfolder green fluorescent protein (msGFP), and HaloTag. This construct localizes HaloTag to the outer mitochondrial membrane (58, 59) and allows straightforward measurement of labeling and capture efficiency using a pulse–chase assay coupled with sodium dodecyl sulfate–polyacrylamide gel electrophoresis (SDS–PAGE) followed by in-gel fluorescence (Fig. 5*A* and *SI Appendix*, Figs. S2 and S3). Evaluation of the labeling efficiency in these cells confirmed that 1 µM unfunctionalized ligands **2_HTL_**–**6_HTL_** showed near complete labeling of the msGFP–HaloTag fusion; by contrast the biotin–JF_549_–HaloTag ligand (**13_HTL_**) gave a low degree of labeling (< 3%) at both 100 nM and 1 µM concentration, consistent with the log*D*_7.4_ measurements (Fig. 3*A*) and imaging results in U2OS cells (Fig. 4 *A*, *E*, *I*, and *M*). The carborhodamine-based biotin–JF_608_–HaloTag ligand (**14_HTL_**) gave substantially higher labeling efficiency: 11.3 ± 6.4% (mean ± SEM; 100 nM) and 57.7 ± 9.6% (1 µM). The Si-rhodamine ligands provided the highest overall labeling efficiencies with JF_646_-based **15_HTL_** showing 13.6 ± 3.7% (100 nM) and 61.7 ± 6.8% (1 µM) and JF_635_-based **16_HTL_** yielding 18.0 ± 4.9% (100 nM) and 69.0 ± 4.2% (1 µM). These results were also consistent with live HEK293T cell imaging experiments in which **13_HTL_** showed low intracellular signal (*SI Appendix*, Fig. S4), whereas Si-rhodamine ligands **15_HTL_** and **16_HTL_** showed robust labeling and colocalization with mitochondrial-localized GFP with intensities consistent with their in vitro brightness properties (Fig. 3*A* and *SI Appendix*, Fig. S5).

We then used **15_HTL_** and **16_HTL_** to purify mitochondria from these cells by incubation followed by washing and cell lysis. The crude supernatant was incubated with streptavidin-coated magnetic microbeads and washed. The bead-bound mitochondria were lysed, and the resulting solution was analyzed by SDS–PAGE/in-gel fluorescence (Fig. 5 *A* and *B*). The biotin-free ligands **5_HTL_** and **6_HTL_** gave no appreciable protein capture, but 100 nM biotin–JF_646_–HaloTag ligand (**15_HTL_**) and biotin–JF_635_–HaloTag ligand (**16_HTL_**) gave substantial capture efficiency of 84.2 ± 1.8% and 88.2 ± 1.1%, respectively (Fig. 5*C*). Use of 10-fold higher concentrations of **15_HTL_** and **16_HTL_** (1 µM) did not increase mitochondria pulldown efficiency (*SI Appendix*, Fig. S6), demonstrating that the numerous HaloTag proteins on each mitochondrion facilitate efficient capture, even without saturating the mitochondrial surface with the biotin-containing ligand. The lower in-gel fluorescence brightness for the wash and bound fractions of **15_HTL_** or **16_HTL_** compared to the wash and bound fractions of their parent dyes **5_HTL_** or **6_HTL_** (Fig. 5 *B* and *C*) stems from the variability of transient transfection and band broadening due to the larger ligand.

We performed the same protocol using the commercial biotin–HaloTag ligand (**17_HTL_**; Fig. 5*D*), which contains the standard PEG_2_–chloroalkane HaloTag ligand directly attached to the biotin carboxyl group. Pulse–chase labeling revealed that **17_HTL_** achieved higher intracellular labeling (32.0 ± 9.3% at 100 nM and 92.1 ± 0.4% at 1 µM; *SI Appendix*, Fig. S7) than ligands **14_HTL_**–**16_HTL_** but negligible mitochondrial capture efficiency at both 100 nM and 1 µM (8.6 ± 1.4%; Fig. 5 *E* and *F*), similar to the no-ligand control (7.0 ± 0.5%; *SI Appendix*, Fig. S8). We surmised the intimate association of ligand and protein in HaloTag conjugates with the short PEG_2_ linker (Fig. 1*C*) results in a biotin species less accessible for streptavidin binding. Previous attempts to remedy this problem by incorporating an extra PEG_4_ linker between the biotin and the chloroalkane yielded a molecule with low cell permeability, (60) rendering it unsuitable for experiments in live cells. Similar observations were made in a concurrent study where an extra PEG_1_ linker was incorporated between the biotin and HaloTag ligand to increase linker length but preserve cell permeability (61). These results demonstrate that the existing commercial biotin ligands are suboptimal for streptavidin-mediated affinity purification using the HaloTag system due to poor cell permeability or insufficient linker length. In contrast, inserting a low *K*_L–Z_ dye like Si-rhodamine between the polar biotin and HaloTag ligand balances cellular permeability and functionality; biotin ligands **15_HTL_** and **16_HTL_** enter cells, efficiently label HaloTag proteins, enable confirmation of protein labeling by microscopy, and present a biotin moiety accessible for affinity capture (Figs. 3 and 4).

### Protein Translocation Using JQ1–Rhodamine–HaloTag Ligands.

**Fig. 6.**
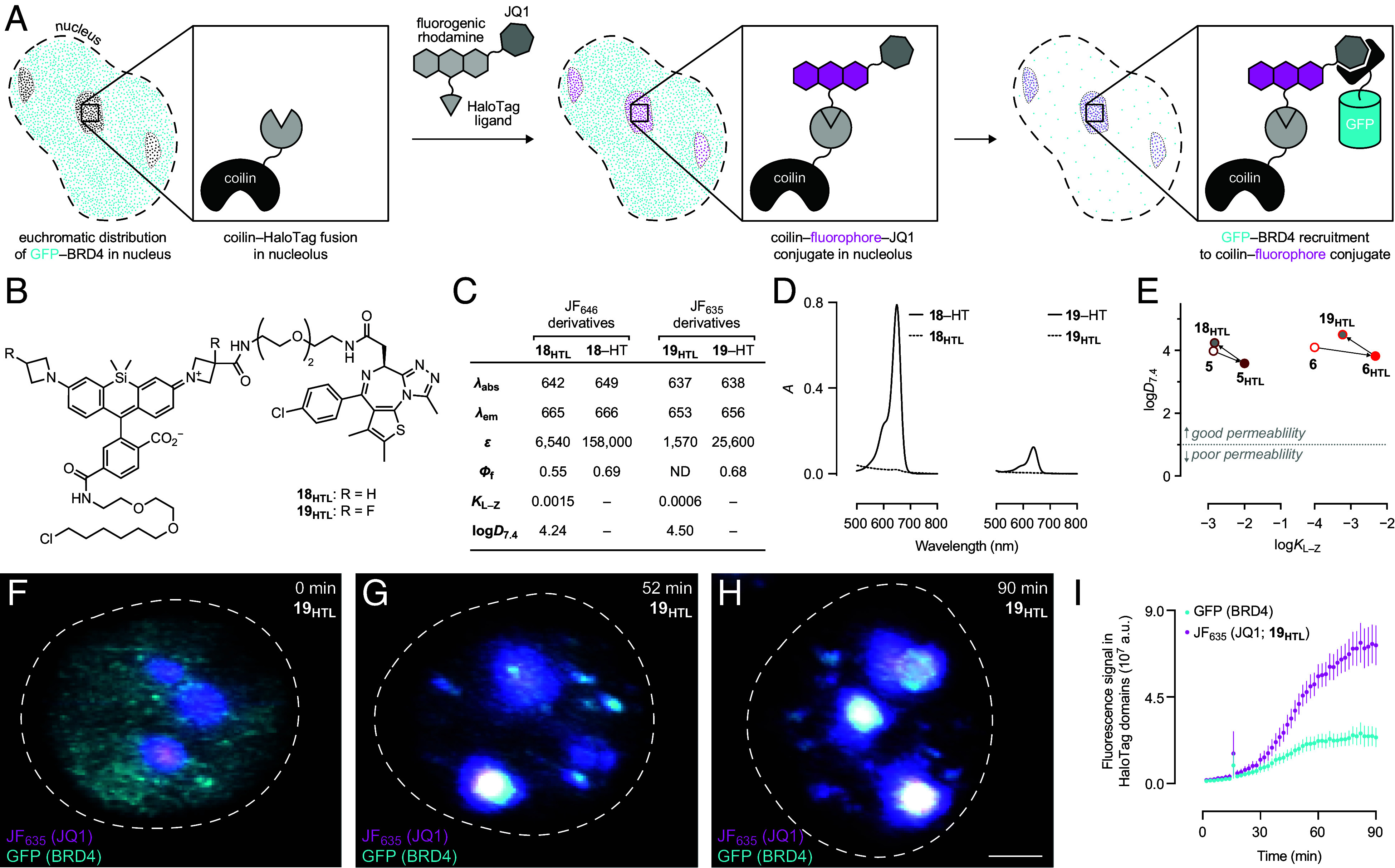
Driving BRD4 translocation using JQ1-containing multifunctional HaloTag ligands. (*A*) Schematic illustrating the translocation of sfGFP–BRD4 using a JQ1-containing multifunctional HaloTag ligand. (*B*) Chemical structures of (*S*)-JQ1–JF_646_–HaloTag ligand (**18**_**HTL**_) and (*S*)-JQ1–JF_635_–HaloTag ligand (**19**_**HTL**_). (*C*) Spectral properties (*λ*_abs_, *λ*_em_, *ε*, and *Φ*_f_), *K*_L–Z_, and log*D_7_*_.4_ of HaloTag ligands **18**_**HTL**_, **19**_**HTL**_, and their HaloTag (HT) conjugates; *λ*_abs_/*λ*_em_ are in nm, and *ε* are in M^−1^cm^−1^. Spectral properties were measured in 10 mM HEPES, pH 7.3 and *K*_L–Z_ measurements were performed in 1:1 (v/v) dioxane–water. The small *ε* of **19**_**HTL**_ prevented accurate measurement of *Φ*_f_. (*D*) Absolute absorbance of **18**_**HTL**_ and **19**_**HTL**_ in the absence or presence (+HT) of excess HaloTag protein. (*E*) Plots of log*D*_7.4_ *vs.* log*K*_L–Z_ for compounds based on JF_646_ (**5**, **5**_**HTL**_, and **18**_**HTL**_), and JF_635_ (**6**, **6**_**HTL**_, and **19**_**HTL**_) showing the consistent shift to higher log*D*_7.4_ values upon installation of the JQ1 moiety; the dotted line denotes region of optimal cellular permeability based on log*D*_7.4_. (*F*–*H*) Representative LLSM images (maximum intensity projections) of Neuro2A cells expressing coilin–HaloTag and sfGFP–BRD4 after incubation with **19**_**HTL**_ at 0, 52, and 90 min timepoints; (Scale bar, 5 μm.) (*I*) Cumulative rhodamine and BRD4 fluorescence intensity within HaloTag domains from LLSM images of Neuro2A cells expressing coilin–HaloTag and sfGFP–BRD4 upon incubation with **19**_**HTL**_; *n* = 13 nuclei; error bars indicate mean ± SEM.

Having validated our design principles with biotin ligands, we next explored pharmacologically active multifunctional compounds. We appended JF–HaloTag ligands with (*S*)-JQ1, an inhibitor of the bromodomain and extraterminal motif (BET) family of proteins including bromodomain containing 4 (BRD4). Our goal was to develop reagents that could be added to cells and elicit rapid translocation of BRD4 to nuclear regions expressing the HaloTag fusions (Fig. 6*A*). We focused on Si-rhodamine-based ligands since they are fluorogenic (Fig. 3*B*) and show efficient cellular labeling (Fig. 4), circumventing the need to wash out the excess compound. As with the biotin compounds (Scheme 1), the 3-carboxyazetidine motif was used as a convenient attachment point for a (*S*)-JQ1 moiety (*SI Appendix*, Scheme S3), yielding (*S*)-JQ1–JF_646_–HaloTag ligand (**18_HTL_**) and (*S*)-JQ1–JF_635_–HaloTag ligand (**19_HTL_**; Fig. 6*B* and *SI Appendix*, Scheme S4).

The trends in spectral properties for **18_HTL_** and **19_HTL_** were similar to their biotin-containing counterparts **15_HTL_** and **16_HTL_**; *λ*_abs_ and *λ*_em_ did not show substantial changes upon HaloTag binding, and the *Φ*_f_ of **18_HTL_** increased (Fig. 6*C*). The lipophilic JQ1 moiety decreased the absorptivity relative to the biotin ligands with JF_646_-based **18_HTL_** showing *ε* = 6,540 M^−1^cm^−1^ and **19_HTL_** exhibiting *ε* = 1,570 M^−1^cm^−1^; binding to the HaloTag ligand increases *ε* to 158,000 M^−1^cm^−1^ and *ε* = 25,600 M^−1^cm^−1^, respectively (Fig. 6*D*). Compounds **18_HTL_** and **19_HTL_** did not show substantial increases in absorption or fluorescence when incubated with recombinant BRD4 (*SI Appendix*, Fig. S9), indicating that the fluorogenic effect observed in cells is mainly driven by HaloTag binding. Installation of the JQ1 functionality also lowered *K*_L–Z_ and increased log*D*_7.4_, but this effect was relatively minor (Fig. 6*E*), similar to the results observed for the biotin-containing Si-rhodamine-based multifunctional ligands. The parent dye properties dominate in these multifunctional compounds even when attaching a hydrophobic moiety like JQ1.

The ability of JQ1 ligands to enter living cells and recruit BRD4 to defined genomic regions was evaluated using lattice light sheet fluorescence microscopy (LLSM). Neuro2a cells were transiently transfected with plasmids encoding three fusion proteins: i) HaloTag protein fused to coilin, which localizes to the nucleolus; ii) superfolder GFP–BRD4; and iii) histone H2B–mCherry as a nuclear marker. The Cajal body component coilin localizes in distinct puncta in the nucleus (62) compared to the diffuse euchromatic distribution of BRD4. Upon addition to cells, both (*S*)-JQ1–JF_646_–HaloTag ligand (**18_HTL_**) and (*S*)-JQ1–JF_635_–HaloTag ligand (**19_HTL_**) rapidly labeled the coilin–HaloTag in a fluorogenic manner (Fig. 6 *F*–*H* and *SI Appendix*, Fig. S11) and induced simultaneous BRD4 accumulation at coilin-rich sites in the nucleus (Fig. 6*I*), consistent with the high mobility of this protein (63, 64). Ligand **18_HTL_** displayed higher fluorescent signal in the far-red channel compared to **19_HTL_** (*SI Appendix*, Fig. S10), in line with the in vitro spectroscopic measurements (Fig. 6*C*). JF_646_-based **18_HTL_** labeled faster than JF_635_-derived **19_HTL_**, perhaps due to its lower log*D*_7.4_, but **19_HTL_** ultimately provided higher GFP–BRD4 signal in the regions of interest (*SI Appendix*, Fig. S10).

**Fig. 7.**
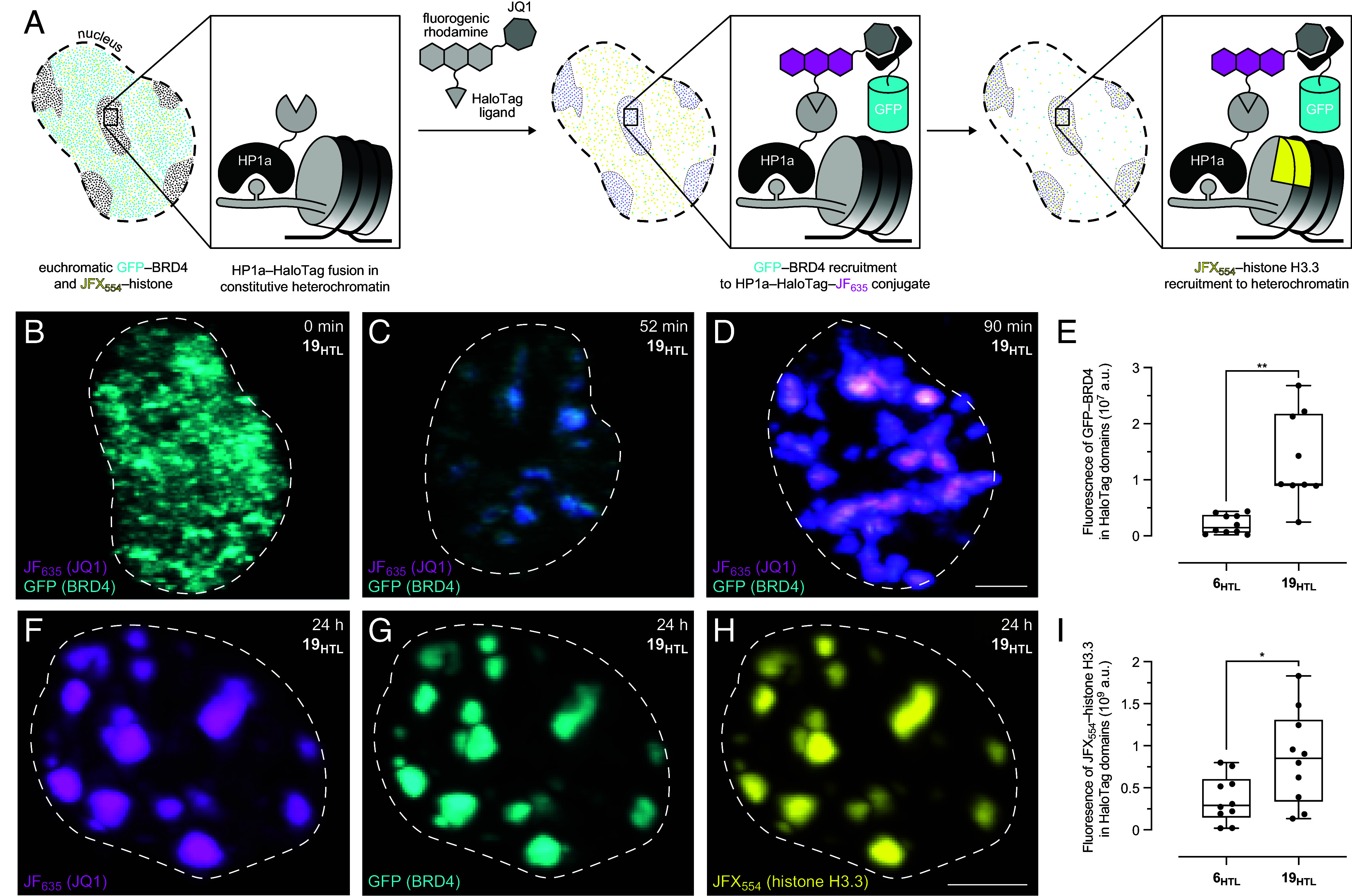
Driving BRD4 to heterochromatin alters chromatin state. (*A*) Schematic illustrating the translocation of sfGFP–BRD4 to constitutive heterochromatin using a JQ1-containing multifunctional HaloTag ligand and resulting histone H3.3 accumulation. (*B*–*D*) Representative LLSM images (maximum intensity projections) of Neuro2A cells expressing HP1a–HaloTag and sfGFP–BRD4 upon incubation with (*S*)-JQ1–JF_635_–HaloTag ligand (**19**_**HTL**_) at 0, 52, and 90 min timepoints; (Scale bar, 5 μm.) (*E*) Box-and-whisker plot of intensity of sfGFP–BRD4 signal within HP1a–HaloTag domains after incubation with JF_635_–HaloTag ligand (6_HTL_) or (*S*)-JQ1–JF_635_–HaloTag ligand (**19**_**HTL**_) at 90 min timepoint; whiskers indicate min–max; *n* ≥ 9 nuclei. (*F*–*H*) Representative LLSM maximum intensity projections of Neuro2A cells expressing HP1a–HaloTag, sfGFP–BRD4, and histone H3.3–SNAP-tag upon incubation with **19**_**HTL**_ and JFX_554_-SNAP-tag ligand (**21**_**STL**_) at 24 h timepoint; (Scale bar, 5 μm.) (*I*) Box-and-whisker plot of intensity of histone H3.3 signal within HP1a–HaloTag domains after incubation with JF_635_–HaloTag ligand (**6**_**HTL**_) or (*S*)-JQ1–JF_635_–HaloTag ligand (19_HTL_) and JFX_554_–SNAP-tag ligand (**21_STL_**) at 24 h timepoint; whiskers indicate min–max; *n* = 10 nuclei. For panels *E* and *I*, two-tailed Welch’s unpaired t-tests were used for significance testing following confirmation of data normality; *P < 0.033 and **P < 0.002.

Given its lower fluorescence background and more efficacious recruitment of BRD4, we focused on (*S*)-JQ1–JF_635_–HaloTag ligand (**19_HTL_**) for subsequent experiments. To test the generality of **19_HTL_** in recruiting BRD4, we imaged cells expressing heterochromatin protein 1a (HP1a) fused to HaloTag (Fig. 7*A*). HP1a is localized within constitutive heterochromatin as its N-terminal chromodomain binds to methylated histone H3 (65). Consistent with the experiments in cells expressing coilin–HaloTag, adding **19_HTL_** to cells with HP1a–HaloTag altered the uniform euchromatic distribution of BRD4 and localized it rapidly to HP1a (Fig. 7 *B*–*D* and *SI Appendix*, Fig. S12). Neither JF_635_–HaloTag ligand (**6_HTL_**), which lacks JQ1, nor (*R*)-JQ1–JF_635_–HaloTag ligand (**20_HTL_**; *SI Appendix*, Scheme S5), which contains the inactive enantiomer (*R*)-JQ1, elicited BRD4 translocation (Fig. 7*E* and *SI Appendix*, Figs. S13 and S14). Incubation with excess free JQ1 also inhibited BRD4 recruitment (*SI Appendix*, Fig. S15).

Finally, we assessed whether the recruitment of BRD4 to the transcriptionally repressed constitutive heterochromatin domain had functional consequences. Cells expressing sfGFP–BRD4, HP1a–HaloTag, and histone H3.3–SNAP-tag were incubated with **19_HTL_** and JFX_554_–SNAP-tag ligand (31) (**21_STL_**). As before, we observed translocation of BRD4 to HP1a–HaloTag domains, and we also saw depletion of histone-H3.3 from euchromatin and accumulation of H3.3 at the HaloTag fusion sites after 24 h (Fig. 7 *F*–*H*); this effect required the JQ1 functionality on the ligand (Fig. 7*I* and *SI Appendix*, Fig. S16). Histone H3.3 is usually absent in constitutive heterochromatin and accumulates in transcriptionally active nucleosomes (66–68). This accumulation of histone-H3.3 in heterochromatin is consistent with previous work using a JQ1-polyamide to translocate BRD4 to a specific gene loci, which resulted in increased transcription by releasing the paused RNA polymerase Pol II (69). Overall, this result demonstrates that targeting JQ1 to HP1a using **19_HTL_** can substantially alter chromatin in living cells.

## Conclusion

The ability to label specific proteins with synthetic small molecules in living cells is a powerful technique for biology (1–3). The generality of these systems has led to the development of many ligands to probe, perturb, or purify cellular components (Fig. 1). Nevertheless, designing cell-permeable ligands remains challenging, especially those with multiple functionalities. We adopted a medicinal chemistry approach by first measuring the log*D*_7.4_ values of free rhodamine dyes and their HaloTag ligands; we compared these against *K*_L–Z_ and found an inverse correlation. This suggested that dyes with low *K*_L–Z_ values, and therefore high distribution coefficients, could be useful scaffolds for creating cell-permeable multifunctional ligands (Fig. 2). We tested this idea by synthesizing biotin-containing HaloTag ligands based on four different dyes with decreasing *K*_L–Z_ values: JF_549_, JF_608_, JF_646_, and JF_635_ (**13_HTL_**–**16_HTL_**; Fig. 3). Ligand **13_HTL_** based on the high *K*_L–Z_/low log*D*_7.4_ dye JF_549_ showed poor intracellular labeling, whereas ligands **14_HTL_**–**16_HTL_**, based on the low *K*_L–Z_/high log*D*_7.4_ dyes JF_608_, JF_646_, and JF_635_, showed more efficient labeling in various subcellular locations (Fig. 4). Ligands **15_HTL_** and **16_HTL_** permitted efficient affinity capture of mitochondria in contrast to the ineffectiveness of the commercial biotin–HaloTag ligand **17_HTL_** (Fig. 5). We extended this strategy to prepare fluorogenic JQ1-containing ligands **18_HTL_** and **19_HTL_**, which efficiently translocated BRD4 from euchromatin to subnuclear regions containing coilin (Fig. 6) or the constitutive heterochromatin marker HP1a. The recruitment of BRD4 to HP1a appeared to increase the transcriptional activity of heterochromatin, as evidenced by a corresponding translocation of histone H3.3 (Fig. 7).

Looking forward, we expect the use of low *K*_L–Z_/high log*D*_7.4_ dyes as scaffolds will generate a palette of useful multifunctional ligands for biology. Although incorporating rhodamine into a complex molecular tool is not without cost, advances in fluorophore chemistry over the past two decades, (34–36) have simplified the synthesis of rhodamines. We have demonstrated that this additional effort is warranted—adding the Si-rhodamine moieties in biotin-containing ligands **15_HTL_** and **16_HTL_** was critical in allowing efficient capture of mitochondria, whereas the commercial ligand **17_HTL_**was too short to give appreciable affinity capture after incubation with living cells. The inclusion of a fluorophore also allowed facile quantification of the labeling in living cells through fluorescence microscopy and capture efficiency using SDS–PAGE. For JQ1-containing ligands **18_HTL_** and **19_HTL_**, the fluorogenic dye was necessary to observe both the labeling of HaloTag fusion proteins and the translocation of BRD4 inside living cells without intermediate washing steps. Overall, designing other multifunctional ligands by leveraging the structure–activity relationships of rhodamines (26, 28–30, 36, 70–72) will yield a valuable toolbox of cell-permeable probes, where what you see is what you get.

## Materials and Methods

Detailed descriptions of the materials and methods are provided in the supporting information. Multifunctional rhodamine–HaloTag ligands (**13_HTL_**–**16_HTL_** and **18_HTL_**–**20_HTL_**) were synthesized using palladium-catalyzed cross-coupling reactions to install 3-carboxyazetidine moieties onto rhodamine scaffolds, followed by amide bond formation with biotin–PEG_2_–NH_2_, (*S*)-JQ1–PEG_2_–NH_2_, or (*R*)-JQ1–PEG_2_–NH_2_. Spectroscopic properties (*λ*_abs_, *λ*_em_, *ε*, and *Φ*_f_) were determined in 10 mM HEPES, pH 7.3. The lactone–zwitterion equilibrium constants (*K*_L–Z_) were measured in 1:1 (v/v) dioxane–water mixtures. Octanol–water distribution coefficients (log*D*_7.4_) were determined by miniaturized shake-flask partitioning experiments using PBS (pH 7.4) and 1-octanol. Cell culture experiments employed HEK293T, U2OS, and Neuro2a cells transfected with HaloTag fusion constructs and labeled with ligands (100 nM to 1 μM, 0.5 to 2 h at 37 °C), followed by fluorescence microscopy using confocal or lattice light-sheet microscopy. Affinity purification studies utilized streptavidin magnetic beads to capture biotin-labeled HaloTag conjugates, with analysis by SDS-PAGE and in-gel fluorescence detection.

## Supplementary Material

Appendix 01 (PDF)

## Data Availability

The authors welcome reasonable requests for pre- and noncommercial materials. All data are included in the manuscript and *SI Appendix* or can be requested from the authors.
