# Supplementary material for: Optimizing multifunctional fluorescent ligands for intracellular labeling
Source: Proc Natl Acad Sci U S A. 2025 Oct 27;122(44):e2510046122. doi: 10.1073/pnas.2510046122 (PMC12595484; doi:10.1073/pnas.2510046122)
Supplement: Supplementary file 1 — Appendix 01 (PDF) [file pnas.2510046122.sapp.pdf]

SI APPENDIX

Optimizing multifunctional fluorescent ligands for intracellular labeling

Pratik Kumar,[a] Jason D. Vevea,[b,c,d] Ariana N. Tkachuk,[a] Kirby R. Campbell,[b] Emma T. Watson,[c,d] Anthony X. Ayala,[a] Jonathan B. Grimm,[a] Edwin R. Chapman,[c,d] David J. Solecki,[b] and Luke D. Lavis[a,*]

[a]Janelia Research Campus, Howard Hughes Medical Institute, 19700 Helix Drive, Ashburn, VA, 20147

[b]Neuronal Cell Biology Division, Department of Developmental Neurobiology, St. Jude Children's Research Hospital, 262 Danny Thomas Place, Memphis, TN, 38104

[c]Department of Neuroscience, University of Wisconsin–Madison, Madison, WI, 53705

[d]Howard Hughes Medical Institute, University of Wisconsin–Madison, Madison, WI, 53705

*Corresponding author email: lavisl@janelia.hhmi.org

Supplementary Figures and Schemes S2
Spectroscopy and Cell Biology Methods S19
Synthetic Organic Chemistry Methods S27
Experimentals and Characterization for All New Compounds S28
NMR Spectra and HPLC Traces S41
References S60

Supplementary Figures and Schemes

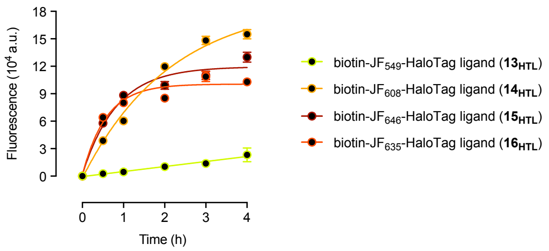

Figure S1. Loading curves for biotin-JF-HaloTag ligands (13$_{HTL}$–16$_{HTL}$). Live U2OS cells stably expressing nuclear-localized HaloTag–histone H2B fusion were incubated with 200 nM of the ligand: biotin-JF$_{549}$-HaloTag ligand (**13**$_{HTL}$); biotin-JF$_{608}$-HaloTag ligand (**14**$_{HTL}$); biotin-JF$_{646}$-HaloTag ligand (**15**$_{HTL}$); biotin-JF$_{635}$-HaloTag ligand (**16**$_{HTL}$); *n* = 3; error bars indicate mean ± SEM.

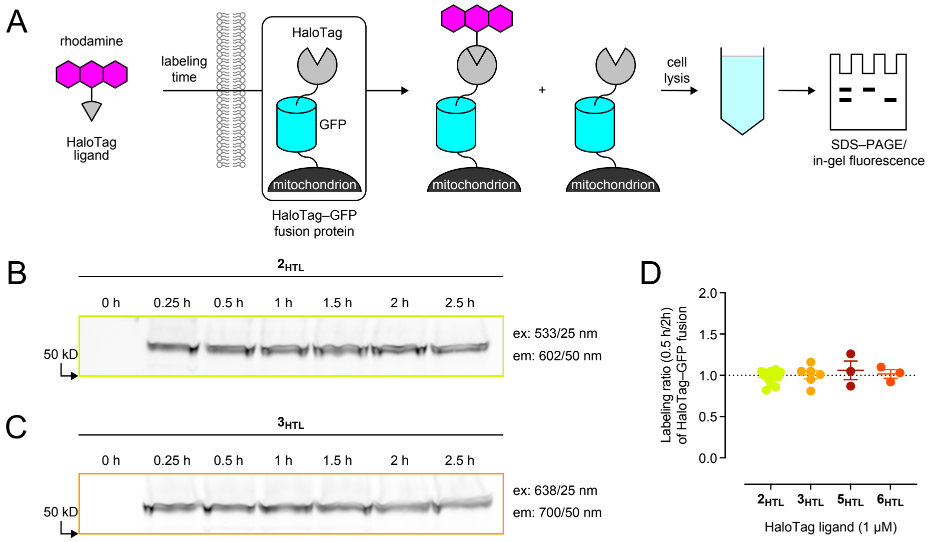

Figure S2. Evaluation of intracellular labeling using JF-HaloTag ligands. (*A*) Schematic of the experimental workflow to evaluate time-dependent labeling after incubation of live HEK293T cells expressing msGFP–HaloTag fusion localized to the mitochondrial outer membrane with JF$_{549}$-HaloTag ligand (**2**$_{HTL}$), JF$_{608}$-HaloTag ligand (**3**$_{HTL}$), JF$_{646}$-HaloTag ligand (**5**$_{HTL}$), and JF$_{635}$-HaloTag ligand (**6**$_{HTL}$). (*B,C*) Representative SDS-PAGE/in-gel fluorescence images for **2**$_{HTL}$ (*B*) or **3**$_{HTL}$ (*C*) for different times. (*D*) Fluorescence ratio of labeling at 0.5 h and 2 h indicating complete labeling of HaloTag fusion in living cells after 0.5 h of incubation with 1 µM JF-HaloTag ligand; *n* ≥ 3; error bars indicate mean ± SEM.

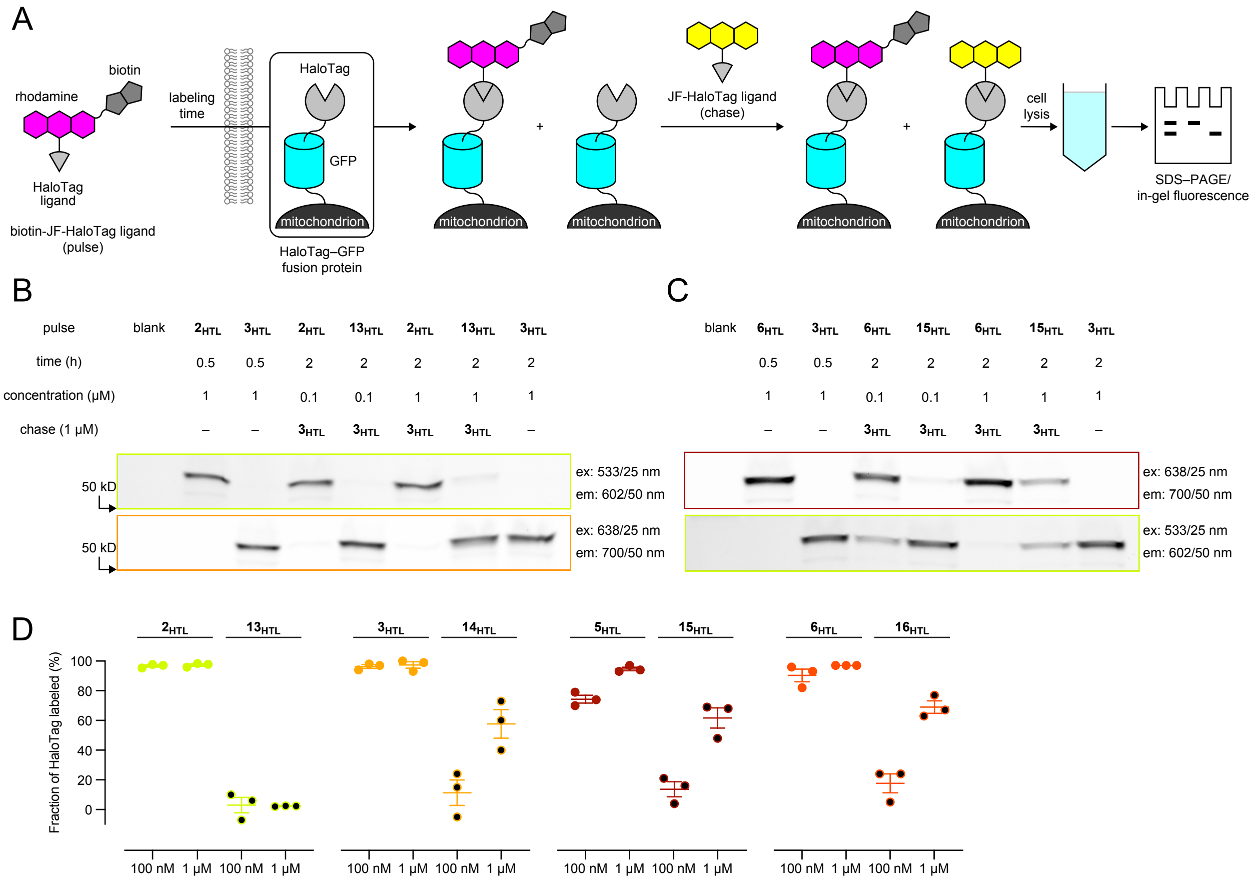

Figure S3. Evaluation of intracellular labeling using biotin-JF-HaloTag ligands. (*A*) Schematic of the experimental workflow to evaluate time-dependent labeling after incubation of live HEK293T cells expressing msGFP–HaloTag fusion localized to the mitochondrial outer membrane with biotin-JF-HaloTag ligands (**13**$_{\mathbf{HTL}}$–**16**$_{\mathbf{HTL}}$; pulse) and JF-HaloTag ligands (**2**$_{\mathbf{HTL}}$ or **3**$_{\mathbf{HTL}}$; chase). (*B,C*) Representative SDS-PAGE/in-gel fluorescence images for biotin-JF$_{549}$-HaloTag ligand (**13**$_{\mathbf{HTL}}$; *B*) and biotin-JF$_{646}$-HaloTag ligand (**15**$_{\mathbf{HTL}}$; *C*). (*D*) Quantification of fraction of HaloTag–msGFP labeled by 100 nM or 1 μM pulse ligands **13**$_{\mathbf{HTL}}$–**16**$_{\mathbf{HTL}}$ after 2 h incubation; *n* = 3; error bars indicate mean ± SEM.

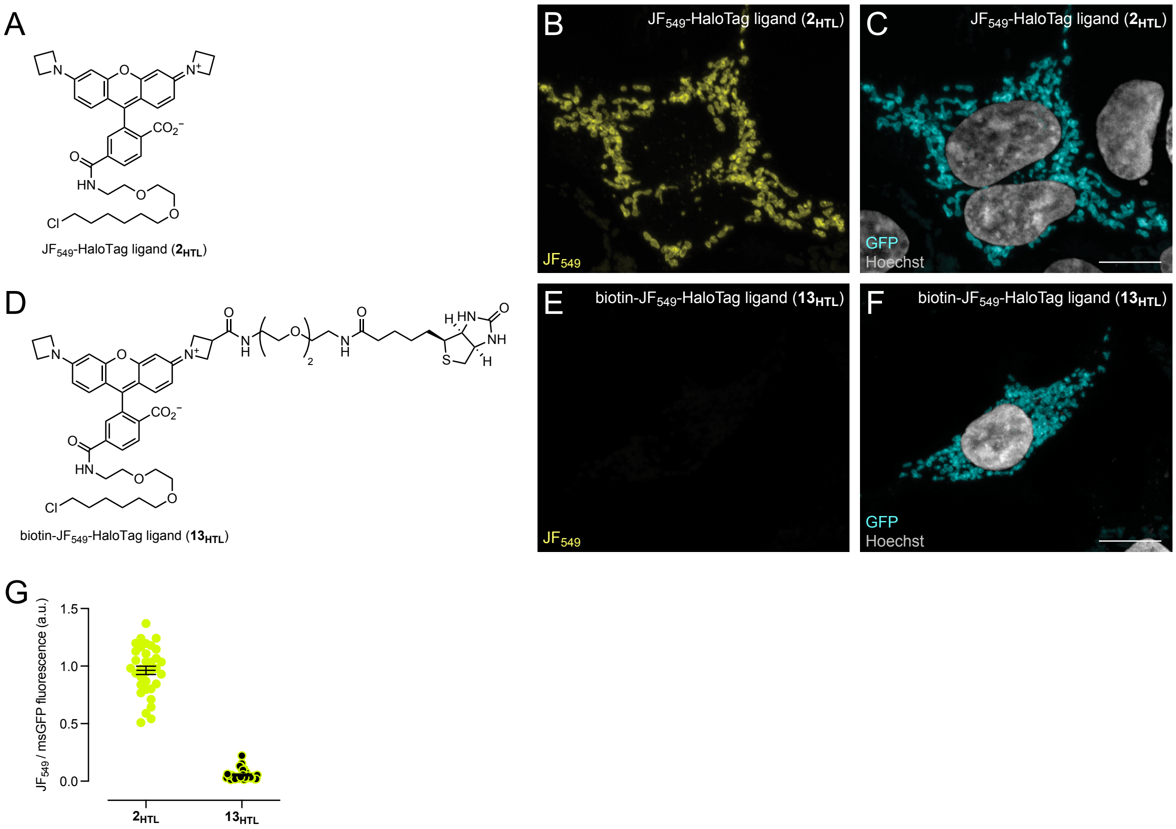

Figure S4. Evaluation of intracellular labeling using biotin-JF549-HaloTag ligand (13HTL) via microscopy. (*A*) Chemical structure of JF549-HaloTag ligand (**2HTL**). (*B,C*) Representative Airyscan fluorescence microscopy images of live HEK293T cells expressing msGFP–HaloTag localized to the mitochondrial outer membrane after incubation with **2HTL** and counterstaining with Hoechst 33342. (*D)* Chemical structure **13HTL**. *(E,F*) Representative Airyscan fluorescence microscopy images of live HEK293T cells expressing msGFP–HaloTag localized to the mitochondrial outer membrane after incubation with **13HTL** and counterstaining with Hoechst 33342. Cells were fixed before imaging; images in *B*/*E* and *C/F* used the same microscope settings; scale bars: 10 µm. (*G*) Quantification of intracellular fluorescence intensity ratio from the live-cell imaging experiments of HaloTag fusion labeled with **2HTL** or **13HTL**; *n* = 34 (**2HTL**) and *n* = 36 (**13HTL**) taken from two independent experiments per ligand with each *n* representing a delineated cell; error bars indicate mean ± SEM. Quantification of intracellular fluorescence intensity ratio from the live-cell imaging experiments of msGFP–HaloTag labeled with 100 nM **2HTL** or **13HTL**; *n* ≥ 3; error bars indicate mean ± SEM.

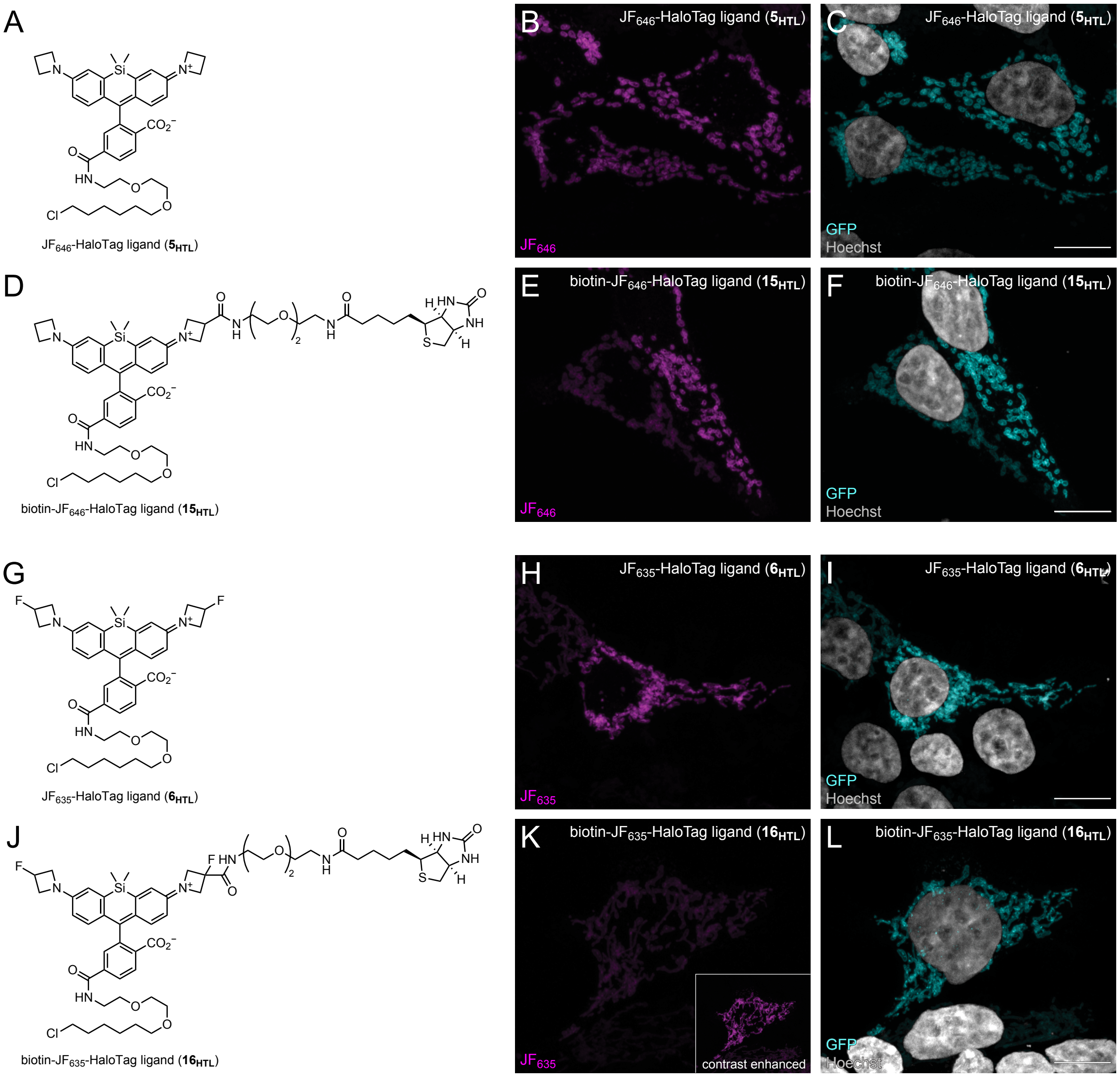

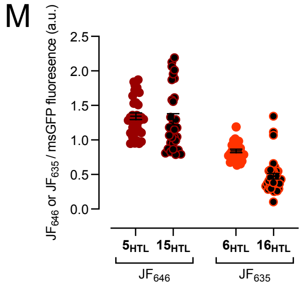

N

Ligand	Pearson's correlation coefficient
JF_{646}-HaloTag ligand (**5_{HTL}**)	0.93
biotin-JF_{646}-HaloTag ligand (**15_{HTL}**)	0.89
JF_{635}-HaloTag ligand (**6_{HTL}**)	0.92
biotin-JF_{635}-HaloTag ligand (**16_{HTL}**)	0.87

Figure S5. Evaluation of intracellular labeling using biotin-JF_{646}-HaloTag ligand (15_{HTL}**) and biotin-JF_{635}-HaloTag ligand (**16_{HTL}**) via microscopy.** (*A*) Chemical structure of JF_{646}-HaloTag ligand (**5_{HTL}**). (*B*,*C*) Representative Airyscan fluorescence microscopy images of live HEK293T cells expressing msGFP–HaloTag localized to the mitochondrial outer membrane after incubation with **5_{HTL}** and counterstaining with Hoechst 33342. (*D*) Chemical structure of **15_{HTL}**. (*E*,*F*) Representative Airyscan fluorescence microscopy images of live HEK293T cells expressing msGFP–HaloTag localized to the mitochondrial outer membrane

after incubation with **15**$_{\mathbf{HTL}}$ and counterstaining with Hoechst 33342. (*G*) Chemical structure of JF$_{635}$-HaloTag ligand (**6**$_{\mathbf{HTL}}$). (*H*,*I*) Representative Airyscan fluorescence microscopy images of live HEK293T cells expressing msGFP–HaloTag localized to the mitochondrial outer membrane after incubation with **6**$_{\mathbf{HTL}}$ and counterstaining with Hoechst 33342. (*J*) Chemical structure of **16**$_{\mathbf{HTL}}$. (*K*,*L*) Representative Airyscan fluorescence microscopy images of live HEK293T cells expressing msGFP–HaloTag localized to the mitochondrial outer membrane after incubation with **16**$_{\mathbf{HTL}}$ and counterstaining with Hoechst 33342. Cells were fixed before imaging; image sets *B/C/E/F* and *H/I/K/L* each used the same microscope settings; the JF$_{635}$ ligands have lower intensity than the JF$_{646}$ ligands under equivalent imaging conditions, which is consistent with the *in vitro* spectral characterization of the HaloTag conjugates; scale bars: 10 μm. (*M*) Quantification of intracellular fluorescence intensity ratio from the live-cell imaging experiments of HaloTag fusion labeled with **5**$_{\mathbf{HTL}}$, **15**$_{\mathbf{HTL}}$, **6**$_{\mathbf{HTL}}$, or **16**$_{\mathbf{HTL}}$; n = 36 (**5**$_{\mathbf{HTL}}$), n = 31 (**15**$_{\mathbf{HTL}}$), n = 30 (**6**$_{\mathbf{HTL}}$), and n = 36 (**16**$_{\mathbf{HTL}}$) taken from two independent experiments per ligand with each n representing a delineated cell; error bars indicate mean ± SEM. *(N)* Pearson's correlation coefficient values between fluorescence signals from rhodamine and GFP in cells labeled with **5**$_{\mathbf{HTL}}$, **15**$_{\mathbf{HTL}}$, **6**$_{\mathbf{HTL}}$, or **16**$_{\mathbf{HTL}}$. Values were obtained using the BIOP JACoP plugin (github.com/BIOP/ijp-jacop-b) with automatic thresholding for image analyses.

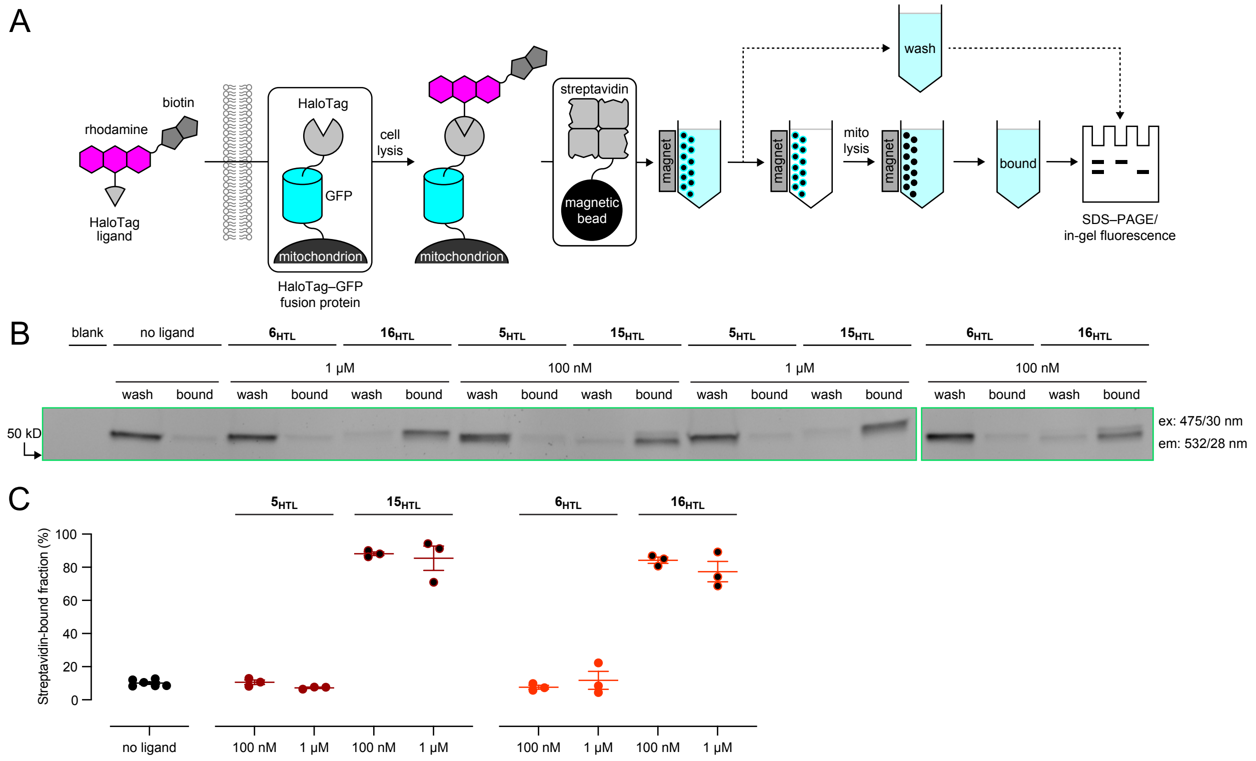

Figure S6. Evaluation of the affinity capture efficiency using biotin-JF-HaloTag ligands. (*A*) Schematic of the experimental workflow to evaluate time-dependent labeling after incubation of live HEK293T cells expressing msGFP–HaloTag fusion localized to the mitochondrial outer membrane with biotin-JF-HaloTag ligands (**15**$_{\mathbf{HTL}}$ and **16**$_{\mathbf{HTL}}$) followed by affinity purification of biotin–HaloTag conjugates. (*B*,*C*) Representative SDS-PAGE/in-gel fluorescence images (*B*) and quantification (*C*) showing the amount of msGFP–HaloTag fusion protein bound to streptavidin after labeling without any ligand, biotin-free parent ligands (**5**$_{\mathbf{HTL}}$ and **6**$_{\mathbf{HTL}}$), and biotin-containing ligands **15**$_{\mathbf{HTL}}$ and **16**$_{\mathbf{HTL}}$; $n \geq 3$; error bars indicate mean ± SEM.

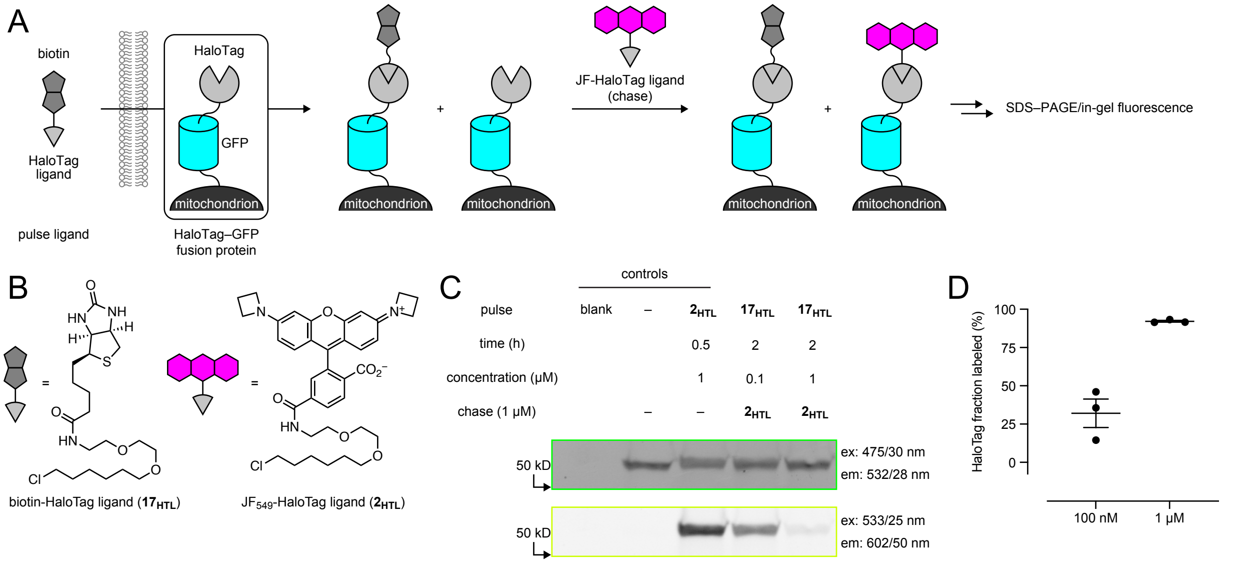

Figure S7. Evaluation of the intracellular labeling efficiency using commercial biotin-HaloTag ligand (17_{HTL}). (*A*) Schematic of the experimental workflow to evaluate time-dependent labeling after incubation of live HEK293T cells expressing msGFP–HaloTag fusion localized to the mitochondrial outer membrane with biotin-HaloTag ligand (**17_{HTL}**; pulse) and JF549-HaloTag ligand (**2_{HTL}**; chase). (*B*) Chemical structures of **17_{HTL}** and **2_{HTL}**. (*C*) Representative SDS-PAGE/in-gel fluorescence images. (*D*) Fraction of HaloTag–msGFP labeled by 100 nM or 1 μM pulse ligand after 2 h incubation; *n* = 3; error bars indicate mean ± SEM.

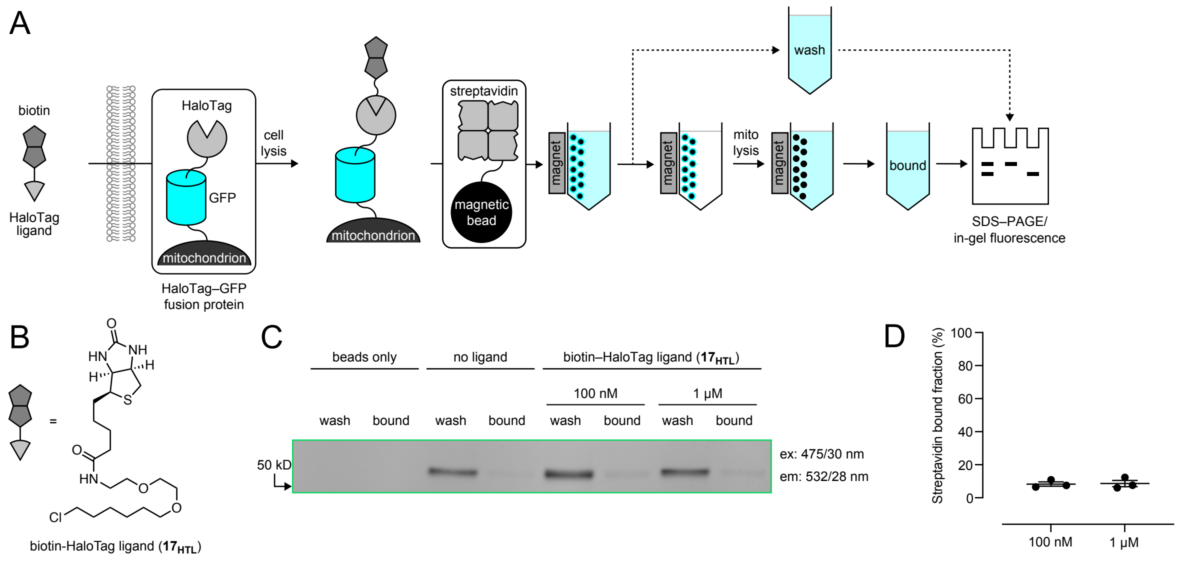

Figure S8. Evaluation of the affinity capture efficiency using biotin-HaloTag ligand (17_{HTL}). (*A*) Schematic of the experimental workflow to evaluate time-dependent labeling after incubation of live HEK293T cells expressing msGFP–HaloTag fusion localized to the mitochondrial outer membrane with biotin-HaloTag ligand (**17_{HTL}**) followed by affinity purification of biotin–HaloTag conjugates. (*B*) Chemical structure of **17_{HTL}**. (*C*) Representative SDS-PAGE/in-gel fluorescence images. (*D*) Fraction of msGFP–HaloTag fusion protein bound to streptavidin after labeling with 100 nM or 1 μM of **17_{HTL}**; *n* = 3; error bars indicate mean ± SEM.

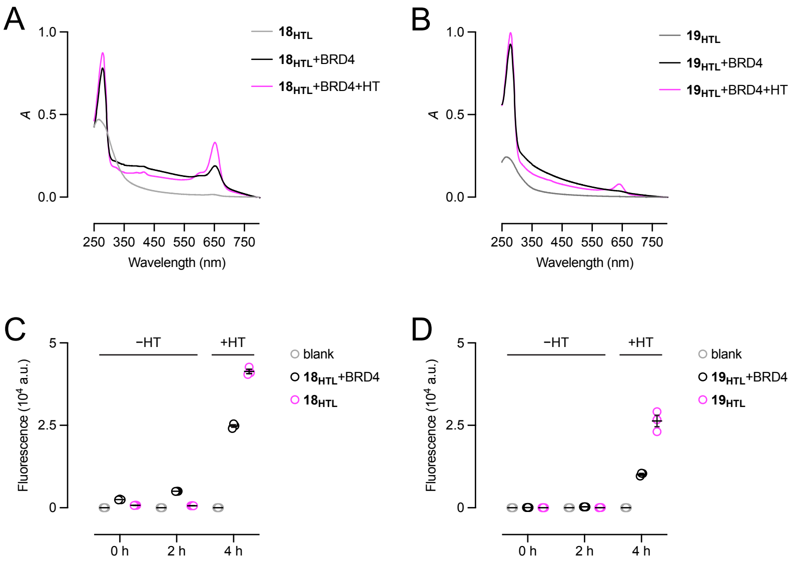

Figure S9. Spectroscopy of (*S*)-JQ1-JF$_{646}$-HaloTag ligand (18$_{HTL}$) and (*S*)-JQ1-JF$_{635}$-HaloTag ligand (19$_{HTL}$) incubated with HaloTag and BRD4. (*A,B*) Absorbance spectra of 5 µM **18$_{HTL}$** (*A*) and **19$_{HTL}$** (*B*) without any protein addition, with 10 µM BRD4, or with 10 µM BRD4 and 10 µM HaloTag. (*C,D*) End-point fluorescence measurements of **18$_{HTL}$** (*C*) and **19$_{HTL}$** (*D*) at 0, 2, and 4 h, without any protein addition, with 10 µM BRD4, or with 10 µM BRD4 and 10 µM HaloTag.; *n* = 3; error bars indicate mean ± SEM.

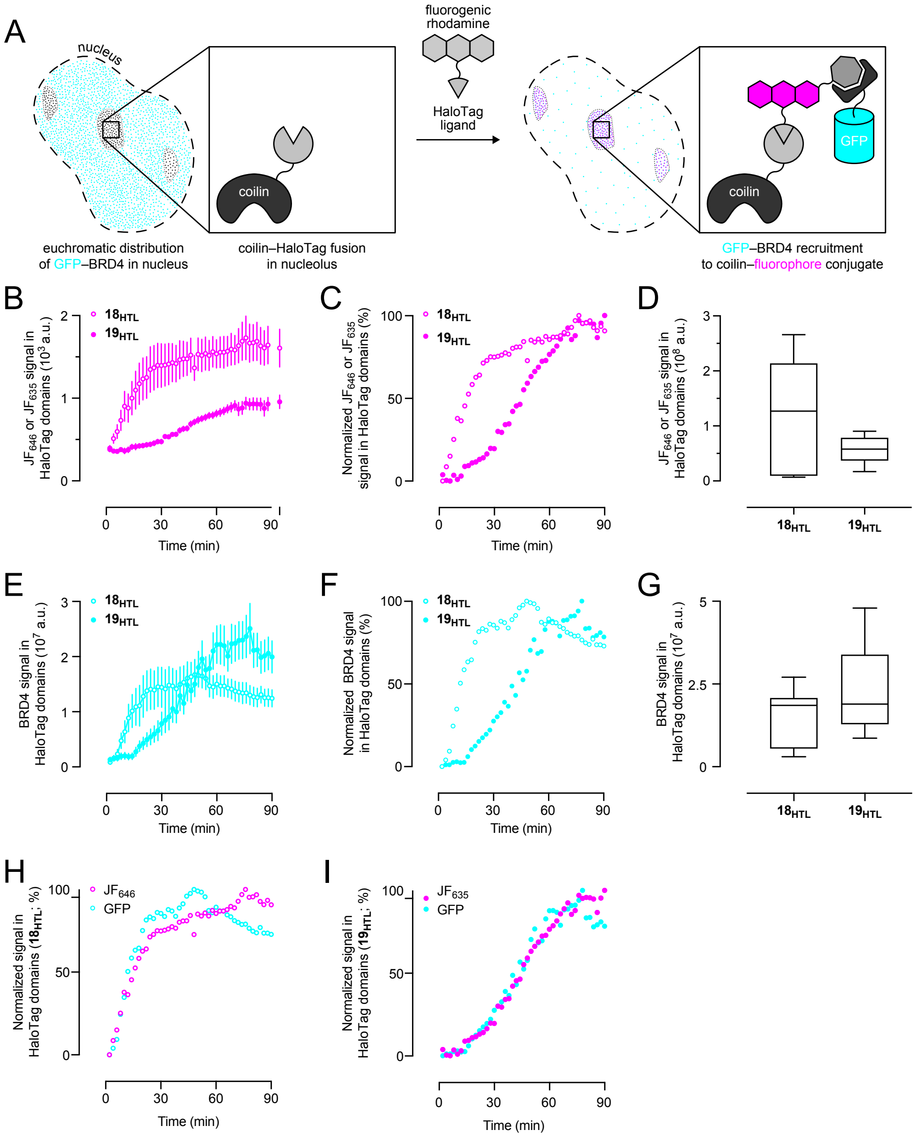

Figure S10. Translocation of BRD4 using (*S*)-JQ1-JF$_{646}$-HaloTag ligand (18$_{HTL}$) and (*S*)-JQ1-JF$_{635}$-HaloTag ligand (19$_{HTL}$). (*A*) Schematic illustrating BRD4 translocation in Neuro2A cells expressing coilin–HaloTag and sfGFP–BRD4 measured by LLSM. (*B,C*) Rhodamine fluorescence signal *vs.* time in HaloTag domains upon addition of **18**$_{HTL}$ (*n* = 13 nuclei; mean ± SEM) or **19**$_{HTL}$ (*n* = 8) as absolute (*B*) or normalized (*C*) plots. (*D*) Box-and-whisker plot of endpoint rhodamine fluorescence (90 min) in HaloTag domains upon addition of **18**$_{HTL}$ or **19**$_{HTL}$; whiskers indicate min–max. (*E,F*) BRD4 fluorescence signal *vs.* time in HaloTag domains upon addition of **18**$_{HTL}$ (*n* = 13) or **19**$_{HTL}$ (*n* = 8) as absolute (*E*) or normalized (*F*) plots. (*G*) Box-and-whisker plot of endpoint BRD4 fluorescence (90 min) in HaloTag domains upon addition of **18**$_{HTL}$ or **19**$_{HTL}$; whiskers indicate min–max. (*H,I*) Replot of data in *C* and *F* showing normalized rhodamine and BRD4 fluorescence *vs.* time upon addition of **18**$_{HTL}$ (*H; n = 13*) or **19**$_{HTL}$ (*I*; *n* = 8). Only mean values are shown in panels *C*, *F*, *H*, and *I* for clarity.

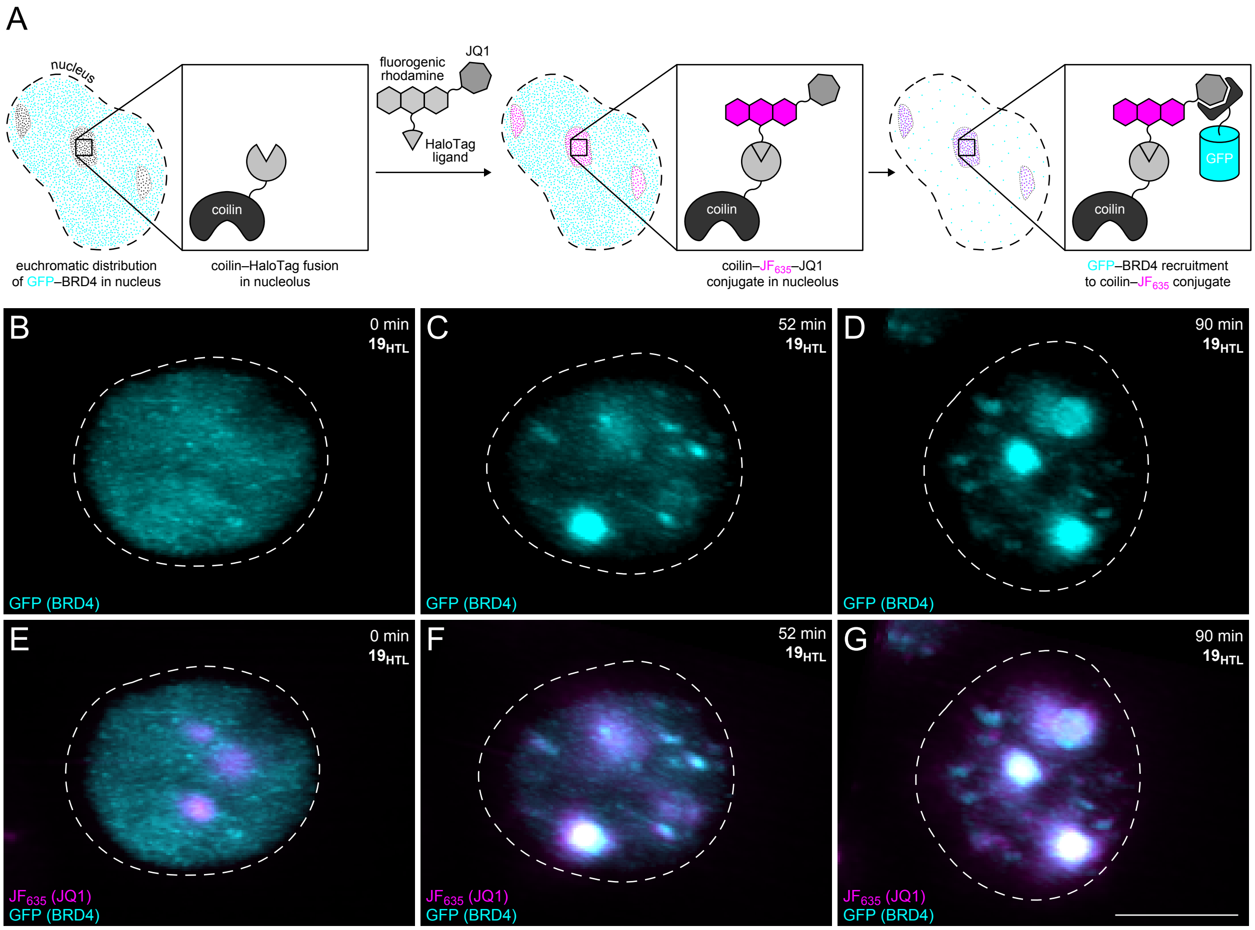

Figure S11. BRD4 recruitment to coilin–HaloTag using (*S*)-JQ1-JF$_{635}$-HaloTag ligand (19$_{HTL}$**).** (*A*) Schematic illustrating BRD4 translocation in Neuro2A cells expressing coilin–HaloTag and sfGFP–BRD4 measured by LLSM. (*B*–*G*) Representative LLSM maximum intensity projections of live Neuro2a cells expressing coilin–HaloTag and sfGFP–BRD4 at 0 min, 52 min, and 90 min after addition of **19$_{HTL}$**. Extracted fluorescence signal from BRD4 alone (*B*–*D*); fluorescence signal from BRD4 and JF$_{635}$ (*E*–*G*); dashed lines represent the nuclear boundary determined through histone H2B–mCherry expression; scale bar: 5 μm.

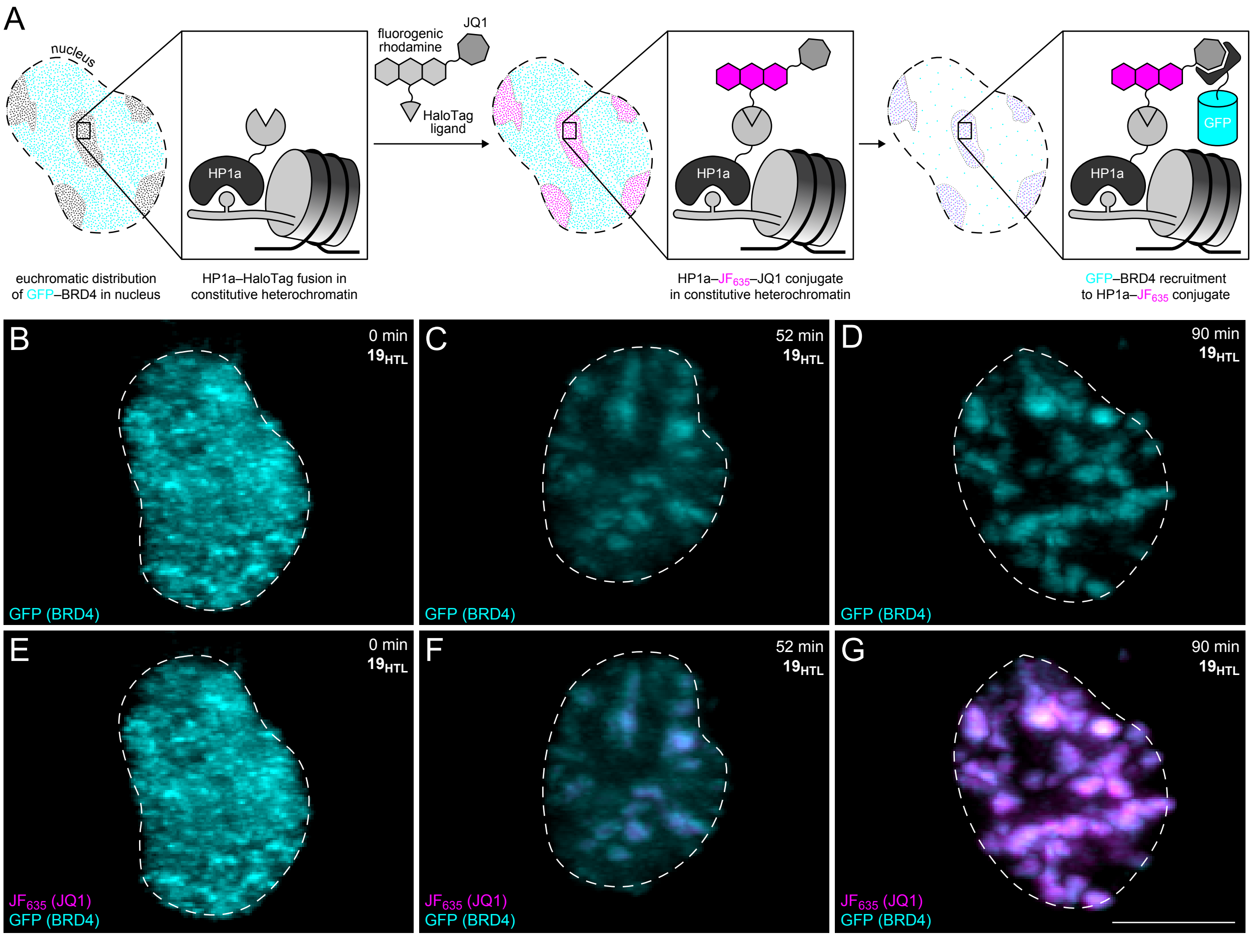

Figure S12. BRD4 recruitment to HP1a–HaloTag using (*S*)-JQ1-JF$_{635}$-HaloTag ligand (19$_{HTL}$). (*A*) Schematic illustrating BRD4 translocation in Neuro2A cells expressing HP1a–HaloTag and sfGFP–BRD4 measured by LLSM. (*B*–*G*) LLSM maximum intensity projections of live Neuro2a cells expressing HP1a–HaloTag and sfGFP–BRD4 at 0 min, 52 min, and 90 min after addition of **19$_{HTL}$**. Extracted fluorescence signal from BRD4 alone (*B*–*D*); fluorescence signal from BRD4 and JF$_{635}$ (*E*–*G*); dashed lines represent the nuclear boundary determined through histone H2B–mCherry expression; scale bar: 5 µm.

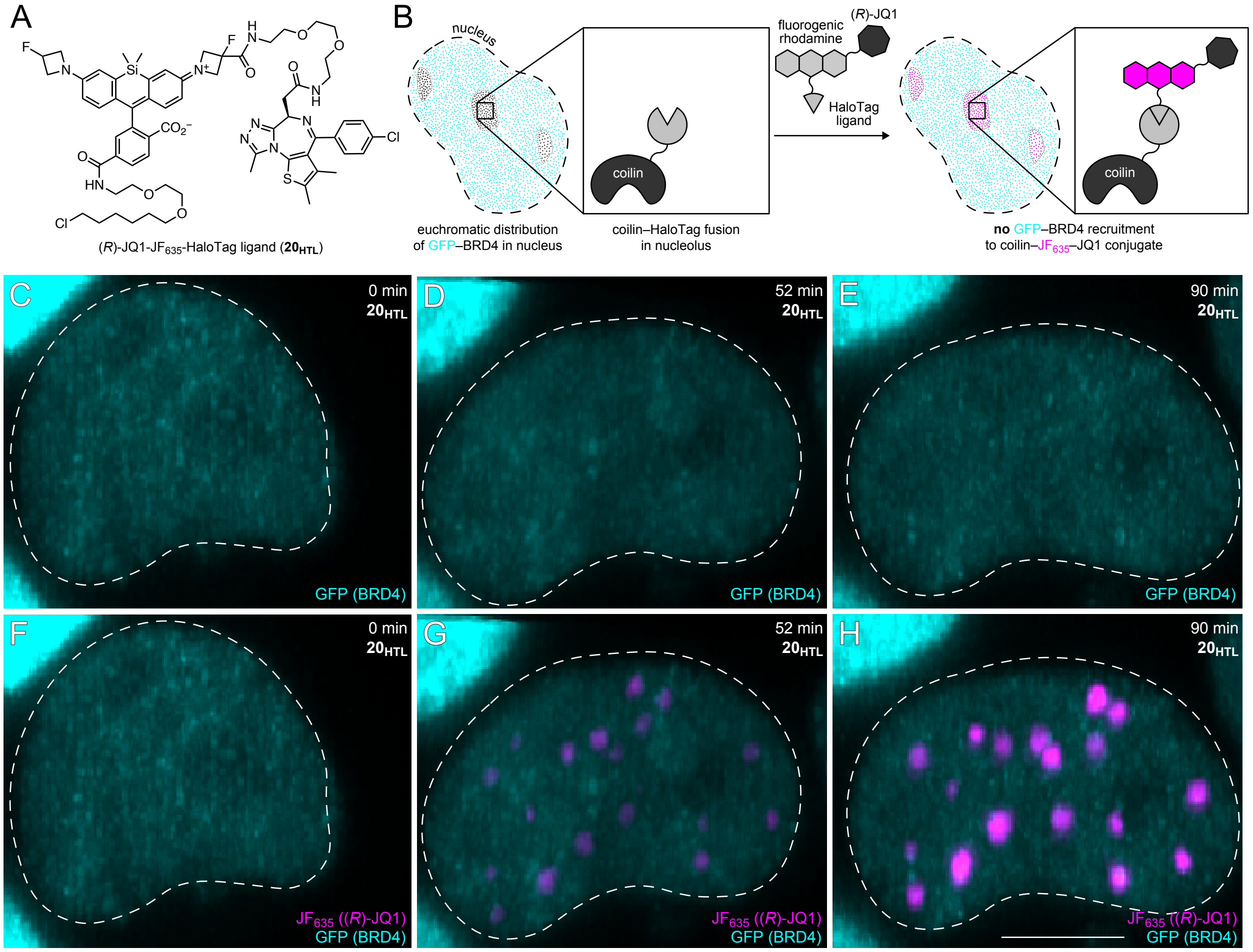

Figure S13. No BRD4 recruitment to coilin–HaloTag using (*R*)-JQ1-JF$_{635}$-HaloTag ligand (20$_{HTL}$). (*A*) Chemical structure **20$_{HTL}$**. (*B*) Schematic illustrating BRD4 translocation in Neuro2A cells expressing coilin–HaloTag and sfGFP–BRD4 measured by LLSM. (*C–H*) LLSM maximum intensity projections of live Neuro2a cells expressing coilin–HaloTag and sfGFP–BRD4 at 0 min, 52 min, and 90 min after addition of **20$_{HTL}$**. Extracted fluorescence signal from BRD4 alone (*C–E*); fluorescence signal from BRD4 and JF$_{635}$ (*F–H*); dashed lines represent the nuclear boundary determined through histone H2B–mCherry expression; scale bar: 5 μm.

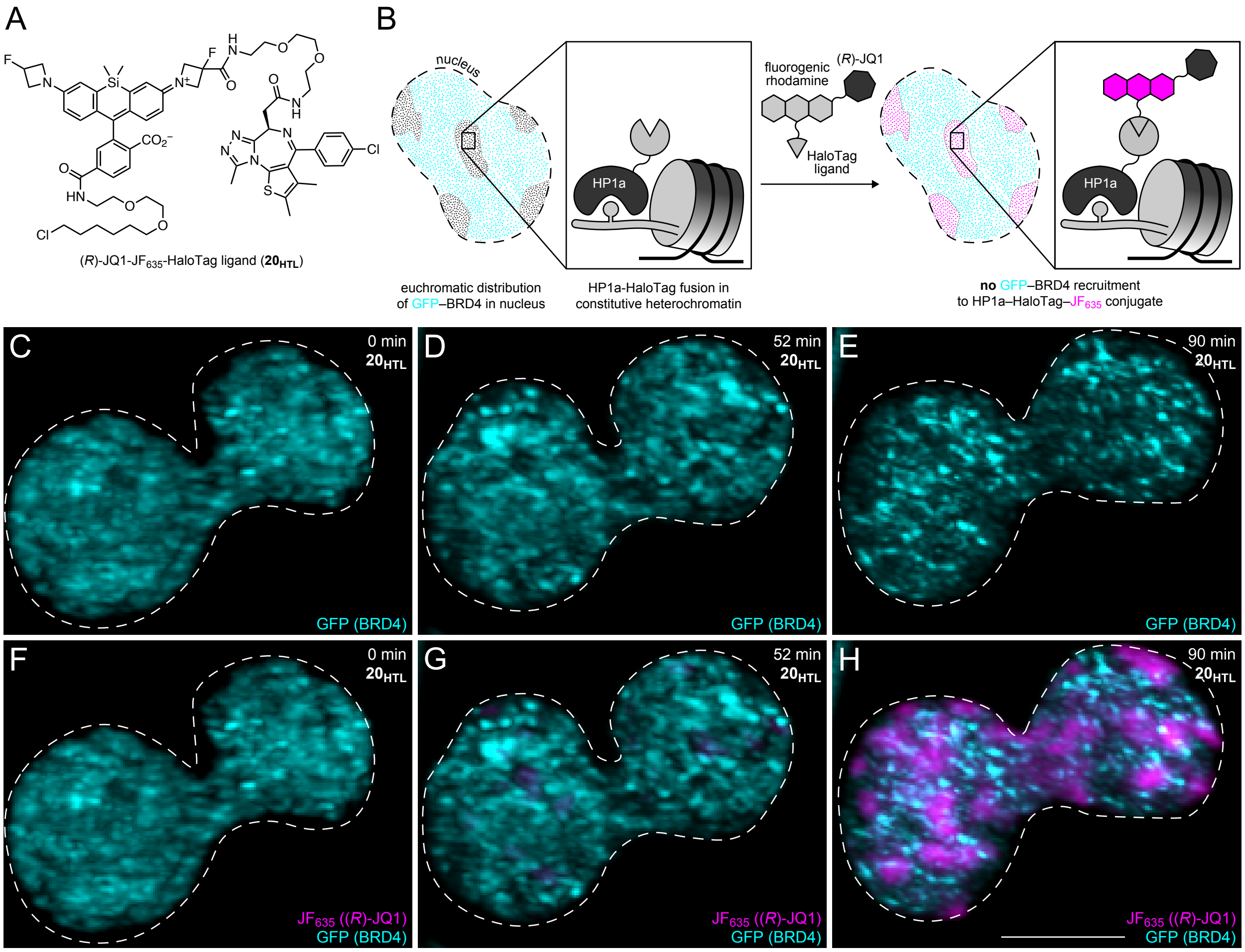

Figure S14. No BRD4 recruitment to HP1a–HaloTag using (*R*)-JQ1-JF$_{635}$-HaloTag ligand (20_{HTL}**).** (*A*) Chemical structure **20_{HTL}**. (*B*) Schematic illustrating BRD4 translocation in Neuro2A cells expressing HP1a–HaloTag and sfGFP–BRD4 measured by LLSM. (*C–H*) LLSM maximum intensity projections of live Neuro2a cells expressing HP1a–HaloTag and sfGFP–BRD4 at 0 min, 52 min, and 90 min after addition of **20_{HTL}**. Extracted fluorescence signal from BRD4 alone (*C–E*); fluorescence signal from BRD4 and JF$_{635}$ (*F–H*); dashed lines represent the nuclear boundary determined through histone H2B–mCherry expression; scale bar: 5 μm.

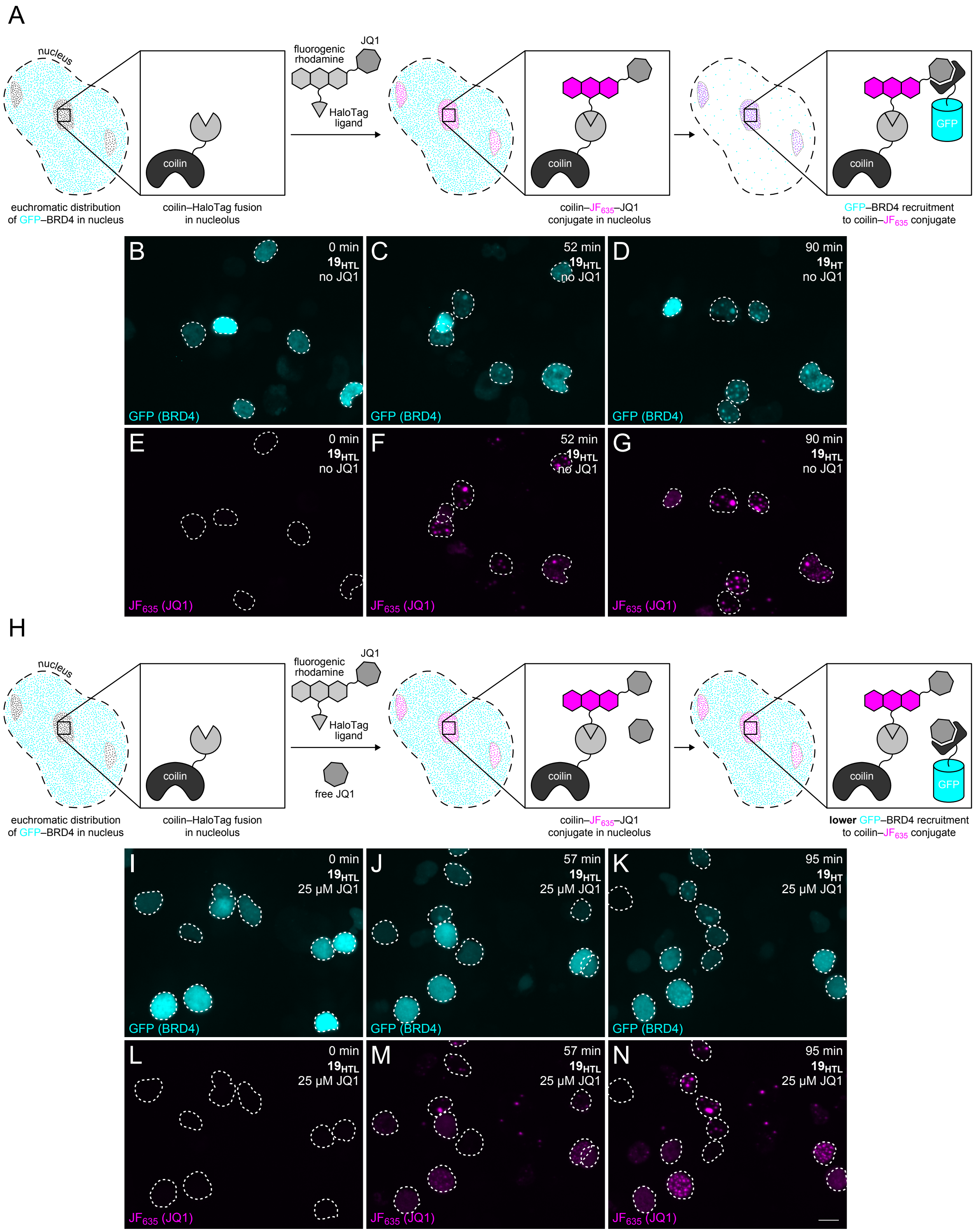

Figure S15. Diminished BRD4 recruitment to coilin–HaloTag using (*S*)-JQ1-JF_{635}-HaloTag ligand (19_{HTL}) and excess (*S*)-JQ1. (*A*) Schematic illustrating BRD4 translocation in Neuro2A cells expressing coilin–HaloTag and sfGFP–BRD4 measured by confocal microscopy. (*B*–*G*) Confocal fluorescence

microscopy maximum intensity projections of live Neuro2a cells expressing coilin–HaloTag and sfGFP–BRD4 at 0 min, 52 min, and 90 min after addition of **19**$_{\mathbf{HTL}}$. Extracted fluorescence signal from GFP (*B*–*D*); fluorescence signal from JF_{635} (*E*–*G*). (*H*) Schematic illustrating BRD4 translocation in Neuro2A cells expressing coilin–HaloTag and sfGFP–BRD4 in the presence of excess (*S*)-JQ1 measured by confocal microscopy. (*I*–*N*) Confocal fluorescence microscopy maximum intensity projections of live Neuro2a cells expressing coilin–HaloTag and sfGFP–BRD4 at 0 min, 57 min, and 95 min after addition of **19**$_{\mathbf{HTL}}$ and (*S*)-JQ1. Extracted fluorescence signal from GFP (*I*–*K*); fluorescence signal from JF_{635} (*L*–*N*). For all images dashed lines represent the nuclear boundary determined through histone H2B–mCherry expression; scale bar: 10 μm.

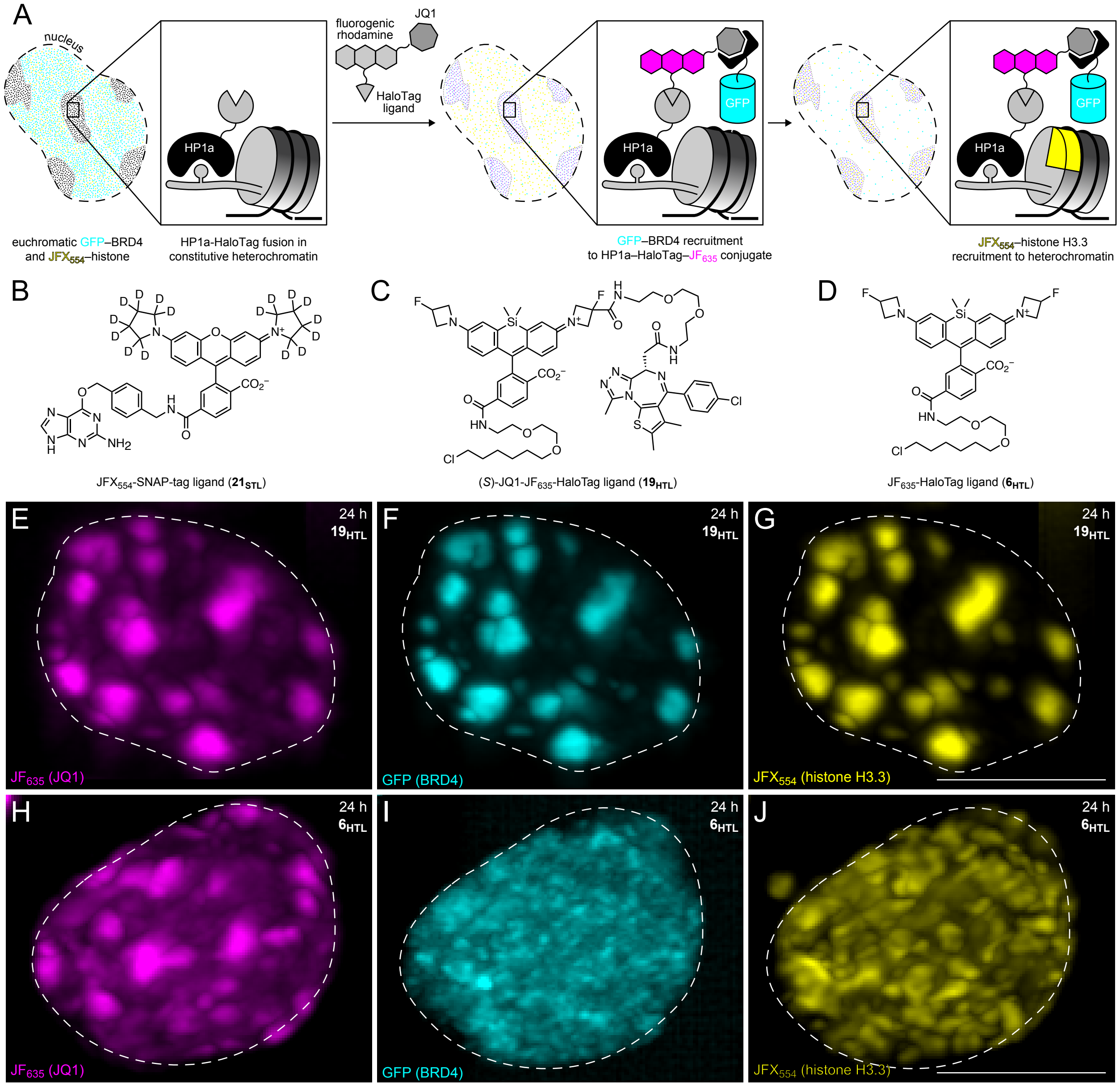

Fig. S16. BRD4 and histone 3.3 recruitment to HP1a–HaloTag using (*S*)-JQ1-JF$_{635}$-HaloTag ligand (19$_{HTL}$). (*A*) Schematic illustrating BRD4 and histone 3.3 translocation in Neuro2A cells expressing HP1a–HaloTag, sfGFP–BRD4, and SNAP-tag–histone 3.3 measured by LLSM. (*B*–*D*) Chemical structures of JFX$_{554}$-SNAP-tag ligand (**21$_{STL}$**; *B*), **19$_{HTL}$** (*C*), and **6$_{HTL}$** (*D*). (*E*–*G*) Representative LLSM maximum intensity projections of live Neuro2a cells expressing HP1a–HaloTag and sfGFP–BRD4 at 90 min after addition of **19$_{HTL}$** ; extracted fluorescence signal from JF$_{635}$ (*E*), BRD4 (*F*), and JFX$_{554}$ (*G*). (*H*–*J*) Representative LLSM maximum intensity projections of live Neuro2a cells expressing HP1a–HaloTag and sfGFP–BRD4 at 90 min after addition of **6$_{HTL}$**; extracted fluorescence signal from JF$_{635}$ (*E*), BRD4 (*F*), and JFX$_{554}$ (*G*). For all images dashed lines represent the nuclear boundary determined through JFX$_{554}$–SNAP-tag–histone 3.3 expression; scale bars: 5 µm.

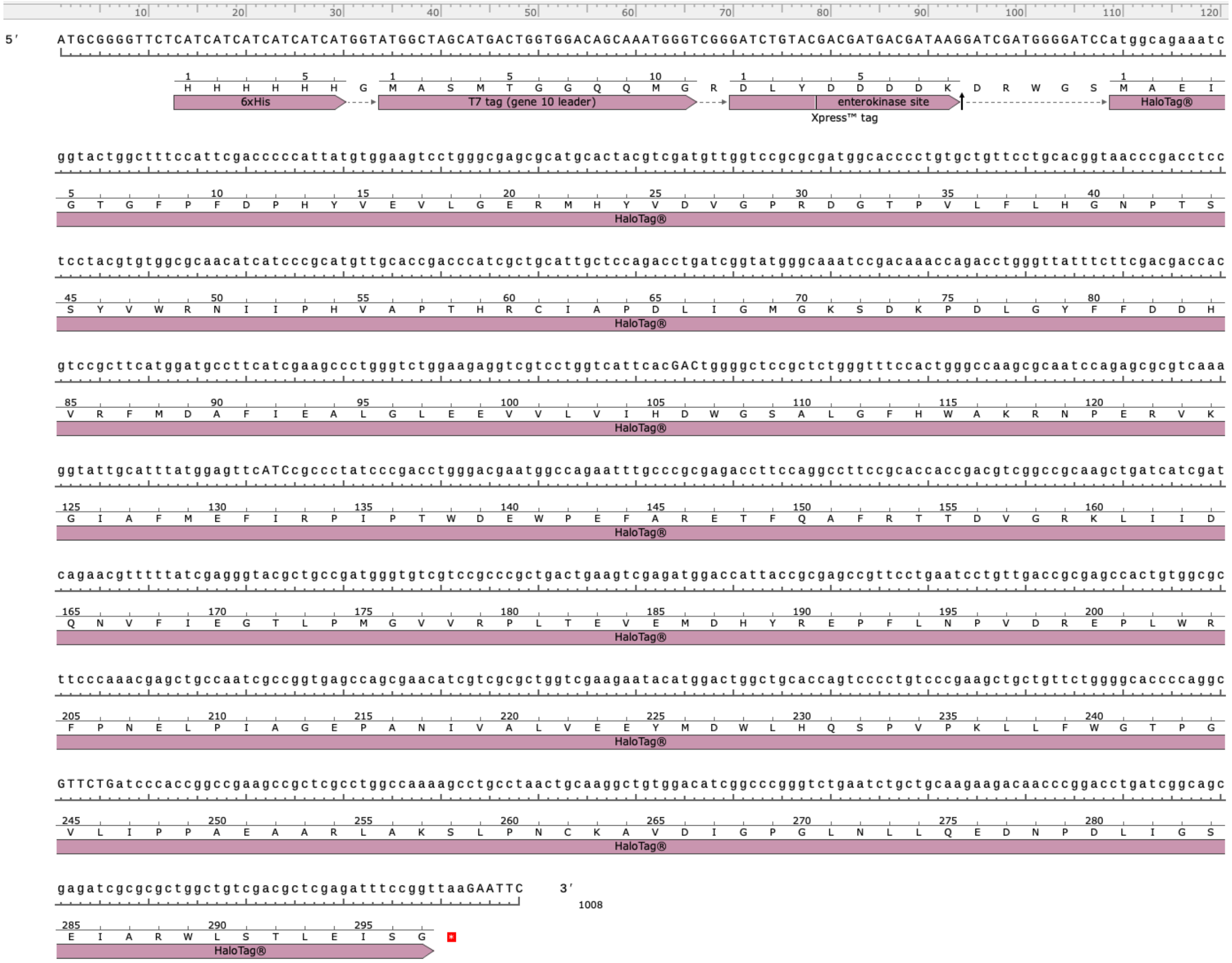

Figure S17. DNA and amino acid sequences of HaloTag used for *in vitro* experiments.

Scheme S1

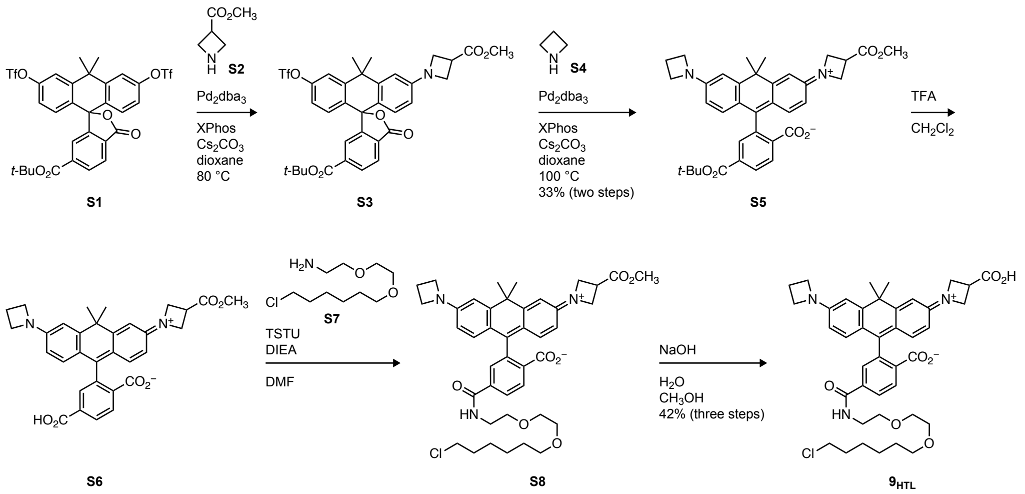

Scheme S2

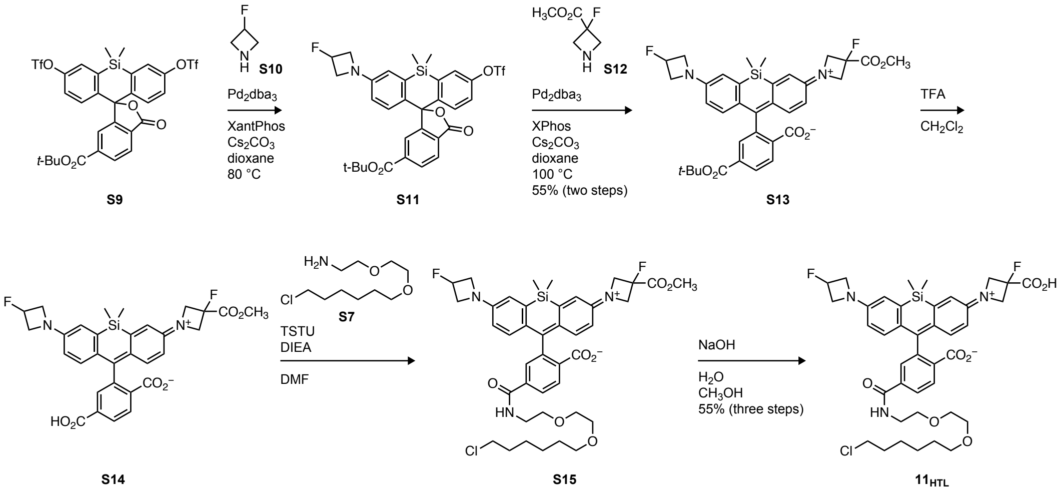

Scheme S3

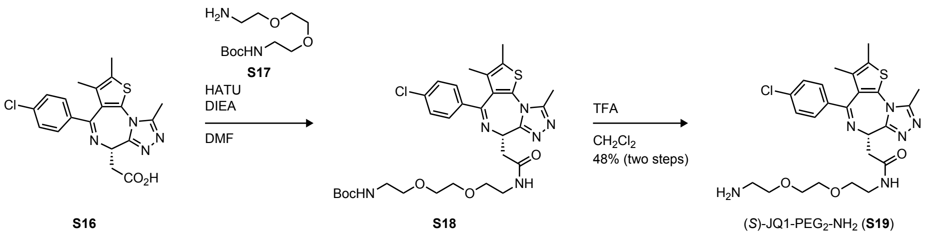

Scheme S4

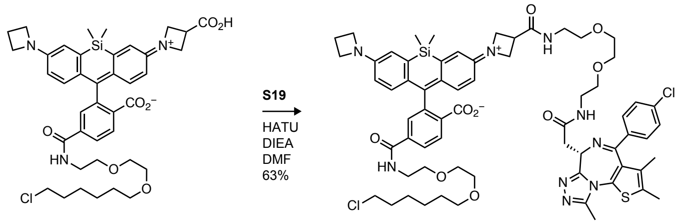

$\mathbf{10_{HTL}}$ (*S*)-JQ1-JF$_{635}$-HaloTag ligand ($\mathbf{18_{HTL}}$)

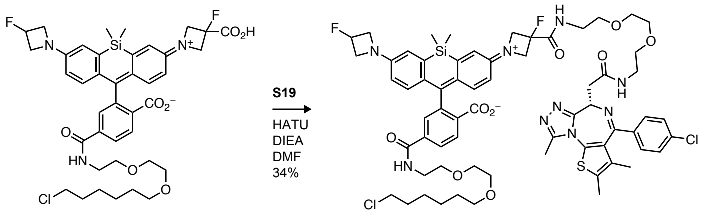

$\mathbf{11_{HTL}}$ (*S*)-JQ1-JF$_{635}$-HaloTag ligand ($\mathbf{19_{HTL}}$)

Scheme S5

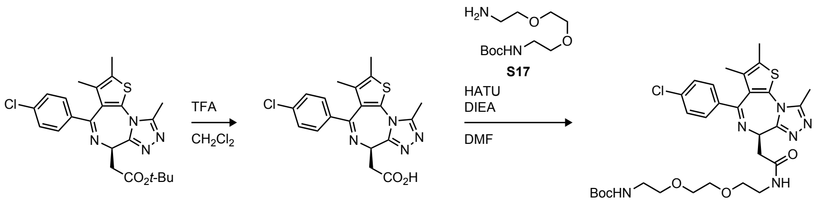

S20 **S21** **S22**

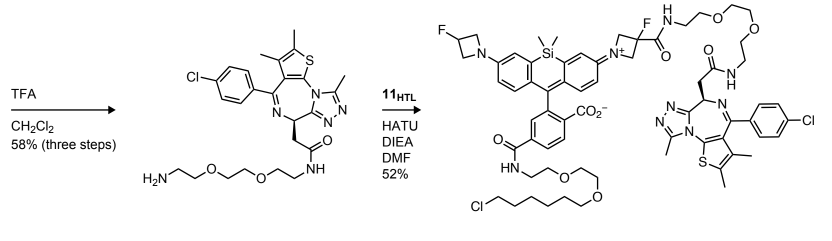

(*R*)-JQ1-PEG$_2$-NH$_2$ (**S23**) (*R*)-JQ1-JF$_{635}$-HaloTag ligand ($\mathbf{20_{HTL}}$)

SPECTROSCOPY AND CELL BIOLOGY METHODS

HaloTag protein purification. The bacterial expression vector pRSET-A (Invitrogen) was used to recombinantly express HaloTag (HT7; Promega).[1] The soluble 6×His-Tagged HaloTag protein was affinity purified by immobilized metal affinity chromatography (IMAC) on a 5-mL Fast Flow HiTrap Sepharose 6 column (Cytiva) with a 0–200 mM imidazole elution gradient using an Avant Protein Purification System (ÄKTA). A_{280} peak fractions were pooled, concentrated by a spin concentrator, and dialyzed 3× into tris-buffered saline (TBS). The amino acid sequence of HaloTag (HT7) expressed from pRSET-A is noted below; the annotated DNA and amino acid sequences are shown in **Figure S17**.

MRGSHHHHHHGMASMTGGQQMGRDLYDDDDKDRWGSMAEIGTGFPFDPHYVEVLG
ERMHYVDVGPRDGTPVLFLHGNPTSSYVWRNIIPHVAPTHRCIAPDLIGMGKSDKPDLG
YFFDDHVRFMDAFIEALGLEEVVLVIHDWGSALGFHWAKRNPERVKGIAFMEFIRPIPT
WDEWPEFARETFQAFRTTDVGRKLIIDQNVFIEGTLPMGVVRPLTEVEMDHYREPFLNP
VDREPLWRFPNELPIAGEPANIVALVEEYMDWLHQSPVPKLLFWGTPGVLIPPAEAARL
AKSLPNCKAVDIGPGLNLLQEDNPDLIGSEIARWLSTLEISG

Spectroscopy of HaloTag ligands and HaloTag conjugates. Compounds **13_{HTL}–16_{HTL}**, **18_{HTL}**, and **19_{HTL}** were prepared as stock solutions in DMSO and diluted such that the final DMSO concentration did not exceed 1% (v/v). All measurements were taken at ambient temperature (22 ± 2 °C). Absorption spectra were recorded on a Cary Model 100 spectrometer (Agilent) using 1-cm path length 1.0-mL quartz microcuvettes from Starna Cells. Fluorescence spectra were recorded on a Cary Eclipse fluorometer (Varian) using 1-cm path length 3.5-mL quartz cuvettes (Starna Cells). As before,[2-4] HaloTag protein was used as a 100 μM solution in 1× TBS. HaloTag ligands **13_{HTL}–16_{HTL}**, **18_{HTL}**, and **19_{HTL}** (5 μM) were dissolved in 10 mM HEPES, pH 7.3, containing 0.1 mg/mL CHAPS. An aliquot of HaloTag protein (1.5 equiv) was added, and the resulting mixture was incubated until a consistent absorbance signal was observed (60–120 min). To measure the fold-increase of absorbance upon HaloTag binding, a "no HaloTag" control experiment was performed where an equivalent volume of TBS blank was added in place of the protein. Reported values for extinction coefficient (ε) are averages of at least two measurements.

Spectroscopy of JQ1-rhodamine-HaloTag ligands and bromodomain-containing protein 4 (BRD4). Compounds **18_{HTL}** and **19_{HTL}** were prepared as stock solutions in DMSO and diluted such that the final DMSO concentration did not exceed 1% (v/v). All measurements were taken at ambient temperature (22 ± 2 °C). Absorption spectra were recorded on a Cary Model 100 spectrometer (Agilent) using 1-cm path length 1.0-mL quartz microcuvettes from Starna Cells. Fluorescence spectra were recorded on a Cary Eclipse fluorometer (Varian) using 1-cm path length 3.5-mL quartz cuvettes (Starna Cells). Commercially obtained recombinant BRD4 (#31880, Active Motif), containing the catalytic core amino acids 44–168, was used as a 100 μM solution.

HaloTag ligands **18**$_{\mathbf{HTL}}$ and **19** $_{\mathbf{HTL}}$ (5 μM) were dissolved in 10 mM HEPES, pH 7.3, containing 0.1 mg/mL CHAPS. An aliquot of BRD4 protein (1.5 equiv) was added, and the absorbance signal of the resulting mixture was recorded at different time points (60–120 min). A "no BRD4" control experiment was also performed where an equivalent volume of TBS blank was added in place of the protein. Reported values for extinction coefficient (ε) are averages of at least two measurements.

Determination of $\boldsymbol{K}_{\mathbf{L-Z}}$. The lactone−zwitterion equilibrium constant ($K_{\mathrm{L-Z}}$) was calculated as described previously[2-4] using equation 1:

$$K_{\mathrm{L-Z}} = \frac{\frac{\varepsilon_{\mathrm{dw}}}{\varepsilon_{\mathrm{max}}}}{\left(1-\frac{\varepsilon_{\mathrm{dw}}}{\varepsilon_{\mathrm{max}}}\right)} \tag{1}$$

where $\varepsilon_{\mathrm{dw}}$ is the extinction coefficient of the dyes in a 1:1 (v/v) dioxane:water solvent mixture containing 0.01% (v/v) triethylamine; this dioxane−water mixture was chosen to give a large range of $K_{\mathrm{L-Z}}$ values,[3] and the triethylamine additive ensures the rhodamines are in the net neutral form. The $\varepsilon_{\mathrm{max}}$ is the maximal extinction coefficient, measured in 0.1% (v/v) trifluoroacetic acid in 2,2,2-trifluoroethanol (TFE) or ethanol.

Quantum yield determination. All reported absolute fluorescence quantum yield values (Φ_{f}) were measured in our laboratory under identical conditions using a Quantaurus-QY spectrometer (model C11374, Hamamatsu). This instrument uses an integrating sphere to determine photons absorbed and emitted by a sample. Measurements were performed using dilute samples (absorbance < 0.1), and self-absorption corrections were performed using the instrument software.[5] Reported values are averages of at least two measurements.

Determination of log$\boldsymbol{D}_{\mathbf{7.4}}$. The log of distribution coefficients at pH 7.4 was determined in octanol–phosphate-buffered saline (PBS) using a miniaturized shake flask setup described previously.[6, 7] Briefly, 150 mL each of octanol and PBS pH 7.4 were stirred vigorously for 12–16 h. The layers were allowed to stand for at least 24 h for phase separation. The separated layers were collected and served as PBS saturated with octanol (PBS*) and octanol saturated with PBS (octanol*). Stock solutions (1 mM or 5 mM) of ligands were prepared in DMSO and diluted in PBS* to obtain 1 μM or 10 μM (3–5 mL) of the standard (std). This standard was used to prepare appropriate dilutions using octanol*, in triplicate, in 2-mL glass vials. The leftover standard was used to determine the standard concentration (C_{std}). The vials were vortexed for 1 min and shaken horizontally at 700 rpm for 2 h. The vials were then shaken upright at 150 rpm for 2 h to allow the phases to separate. The octanol* layer was pipetted out, and the PBS* layer was used for further analyses. The concentrations of ligands in the standard and PBS* ($C_{\mathrm{PBS*}}$) were determined via fluorescence measurements of 200 μL of PBS* or std in 96-well plates on the Cytation 5 Multimode Plate Reader (BioTek). For Si-rhodamine containing ligands, 2–20 μL of

trifluoroacetic acid was added to each well of the 96-well plate prior to the fluorescence measurements. Reported values are averages of at least two measurements. The $\log D_{7.4}$ was obtained as described previously using equation 2:

$$\log D_{7.4} = \log\left(\left(\frac{C_{\mathrm{std}}}{C_{\mathrm{PBS*}}} \times r - 1\right)\frac{V_{\mathrm{PBS*}}}{V_{\mathrm{octanol*}}}\right) \quad (2)$$

where C_{std} is the standard concentration in PBS, $C_{\mathrm{PBS*}}$ is the concentration in PBS after partitioning, r is the dilution factor of the standard solution, $V_{\mathrm{PBS*}}$ is the volume of PBS used in the partitioning, and $V_{\mathrm{octanol*}}$ is the volume of octanol used in the partitioning.

Plasmid construction for mitochondria imaging and affinity purification. HaloTag, amplified from the pHTC HaloTag CMV-neo Vector (Promega; G7711) and monomeric-superfolder green fluorescent protein (msGFP; lab stock), was fused to the mitochondrial targeting signal from OMP25,[8] interspacing flexible $GS(GSS)_4$ linkers between each domain by PCR Splicing by Overlap Extension (SOE). This construct was subcloned into hSynapsin promoter bearing a copy of FUGW.[9] FUGW was a gift from David Baltimore (Addgene plasmid # 14883; RRID: Addgene_14883).[10] This construct was designated as pF(UG) hSyn HaloTag-TEV-4×GS-msGFP-mito.

HEK293T cell culture. HEK293T cells (ATCC) were passaged before 80% confluency by trypsinization (Corning; 25-053-CI) and trituration. The cell suspension was then plated on glass coverslips (Warner instruments; 64-0734, CS-18R17) coated with poly-D-lysine (Thermofisher; ICN10269491) and cultured in Dulbecco's modified Eagle medium (DMEM; ThermoFisher; 11965-118) supplemented with 10% (v/v) fetal bovine serum (FBS, Atlanta Biological) and penicillin/streptomycin (pen/strep; ThermoFisher; MT-30-001-CI) at 37 °C in a humidified 5% (v/v) CO_2 environment following ATCC guidelines. These HEK293T cells were tested for mycoplasma contamination using the Universal Mycoplasma Detection Kit (ATCC; 30-1012K) and validated using Short Tandem Repeat profiling by ATCC (ATCC; 135-XV) within the previous year or were from newly purchased stock from ATCC. HEK293T cells were transfected with pF(UG) hSyn HaloTag-TEV-4×GS-msGFP-mito using a standard calcium phosphate protocol[11] or using lipofectamine LTX reagent (ThermoFisher; 15338100). We note that this plasmid is optimized for lentivirus-mediated expression, but robust expression in HEK293T cells was observed.

HEK293T labeling and fluorescence microscopy. One day after HEK293T cell transfection, the GFP fluorescence signal was confirmed, and HaloTag ligand labeling experiments were conducted. For pulse–chase experiments, HEK293T cells were incubated with biotin-HaloTag ligand ($\mathbf{17}_{\mathbf{HTL}}$; #G8281, Promega) at either 100 nM or 10 μM for 1 hour at 37 °C and chased with 100 nM of JF_{549}-HaloTag ligand ($\mathbf{2}_{\mathbf{HTL}}$). We also evaluated a commercial biotin-HaloTag ligand

with a longer PEG linker (**18_{HTL}**, #G8591, Promega) and confirmed this is not cell-permeant, as indicated in the product information[12] and from a previous publication.[13] For labeling with biotin-JF-HaloTag ligands **13_{HTL}–16_{HTL}**, cells were incubated with 100 nM or 1 μM ligand for 0.5–2 h at 37 °C. For imaging, the coverslips containing HEK293T cells were fixed with 4% (w/v) paraformaldehyde in PBS at 37 °C for 10 min, washed with PBS (3×), and mounted on glass slides with ProLong Glass Antifade Mountant with NucBlue™ Stain (ThermoFisher; P36981). These cells were imaged using a Zeiss LSM 880 with Airyscan and a plan-apochromatic 63×/1.40 oil objective in Fast Airyscan mode. The same image acquisition settings were used for each trial and condition. Images were then processed with automatic Airyscan deconvolution settings. For quantitation of labeling intensity, ROIs were drawn surrounding individual HEK cells, and the average fluorescence ratio (JF/msGFP) from multiple cells in each condition was measured; no background subtraction was necessary.

U2OS cell culture, labeling, and fluorescence microscopy. U2OS cells (ATCC) were cultured in Dulbecco's modified Eagle medium (DMEM, phenol red-free; Life Technologies) supplemented with 10% (v/v) fetal bovine serum (FBS, Life Technologies), 1 mM GlutaMAX (Life Technologies) and maintained at 37 °C in a humidified 5% (v/v) CO_2 environment. These cell lines undergo regular mycoplasma testing by the Janelia Cell Culture Facility. U2OS cells stably expressing an integrated HaloTag–histone H2B fusion protein (U2OS.H2B.HaloTag) were used for nuclear imaging and imaged 18–24 h post-plating. For other subcellular targets, U2OS cells were transiently transfected using nucleofection (Lonza) with plasmids constitutively expressing the following fusion proteins: a C-terminal transmembrane anchoring domain from platelet-derived growth factor receptor (PDGFR) fused to the HaloTag protein (HaloTag–PDGFR; for extracellular display); a HaloTag–TOMM20 fusion protein (outer mitochondrial membrane; Addgene plasmid # 123284; RRID: Addgene_123284); or HaloTag–Sec61β fusion protein (endoplasmic reticulum membrane; Addgene plasmid # 123285; RRID: Addgene_123285). The transiently transfected cells were imaged 18–24 h post-transfection. The stable and transiently transfected U2OS cells were incubated with 100 nM biotin-JF_{549}-HaloTag ligand (**13_{HTL}**), biotin-JF_{608}-HaloTag ligand (**14_{HTL}**), biotin-JF_{646}-HaloTag ligand (**15_{HTL}**), or biotin-JF_{635}-HaloTag ligand (**16_{HTL}**) for 1 h at 37 °C, washed 3× with dye-free media, then fixed with 4% paraformaldehyde in 0.1 M phosphate buffer for 15 min at 37 °C. Fixed cells were then washed 3× in 1× PBS and incubated with Hoechst 33342 (5μg/mL) for 15 min at 22 °C as a nuclear counterstain. Airyscan imaging was performed on a Zeiss LSM 980 with Airyscan 2 confocal microscope using a Plan APO 63×/1.4 oil DIC M27 objective. The same acquisition settings were used for all constructs labeled with **13_{HTL}–16_{HTL}**. These single plane images were bulk processed in ZEN Blue (Zeiss) with automatic Airyscan settings.

Dye loading kinetics. Live U2OS.H2B.HaloTag stable cells were labeled over a time course of 0–4 h with 200 nM of biotin-JF_{549}-HaloTag ligand (**13_{HTL}**), biotin-JF_{608}-HaloTag ligand (**14_{HTL}**), biotin-JF_{646}-HaloTag ligand (**15_{HTL}**), or biotin-JF_{635}-HaloTag ligand (**16_{HTL}**) at 37 °C. Cells were

then washed 3× with dye-free media, fixed with 4% paraformaldehyde in 0.1 M phosphate buffer, pH 7.4 for 15 min at 37 °C. Confocal imaging was performed on a Leica SP8 with an HC PL APO CS2 20×/0.75 immersion objective using the tunable white light laser (WLL) to excite dyes at their λ_{abs} in constant power mode. Fluorescence was quantified as the average integrated density of background corrected nuclear signals from confocal image stack projections analyzed in Fiji;[14] $n = 100$ nuclear signals per compound.

Affinity capture and in-gel fluorescence quantification. Affinity capture pulldown isolation experiments of biotin-labeled HaloTag fusion proteins were performed on ice-cold isolation buffer (IB) consisting of KCl (0.18 M), ethylene glycol-bis(β-aminoethyl ether)-*N*,*N*,*N*′,*N*′-tetraacetic acid (EGTA; 1 mM), 3-(*N*-morpholino)propanesulfonic acid (MOPS; 5 mM); the pH was adjusted to pH = 7.35 using KOH(aq).[15] StrepTactin Microbeads (IBA Lifesciences, 6-5510-050) were used as capture reagents as these beads had lowest amount of background observed in our hands. These beads were kept at 4 °C and blocked in IB buffer with PMSF and 1% (v/v) bovine serum albumin (BSA; Jackson ImmunoResearch, 001-000-162) with rotation at 4 °C for 30 min. HEK293T cells were scraped with 200 µL IB and homogenized using 12 strokes with a 27G syringe. The resulting homogenate was spun at 4 °C at 800 g for 5 min to pellet large cellular debris. 50 µL of freshly blocked StrepTactin microbeads were added to the supernatant and incubated for 30 min at 4 °C with slow rotation. After incubation, tubes were placed in DynaMag2 (ThermoFisher, 12321D) magnetic racks and washed twice with IB+PMSF. All wash steps were kept at 4 °C (cold room). The post-bead supernatant and wash volumes were pooled and spun at 15,000 g for 12 min. This last step pelleted the unbound mitochondria for final analysis of capture efficiency. The bead fraction and the pooled/pelleted fraction were then solubilized using the lysis buffer containing 1× PBS, 2% (v/v) SDS, 1% (v/v) Triton x-100, 10 mM EDTA, and protease inhibitors (2 mM PMSF, aprotinin, leupeptin, and pepstatin A). Samples were then incubated at 50 °C for 3–4 min after the addition of Laemmli sample buffer (SB; Bio-Rad, 1610747) with β-mercaptoethanol (Bio-Rad, 1610710). For protein detection, 10–20 µL of the sample was subjected to SDS-PAGE, using 10% Tris-Glycine gels (TGX) (Bio-Rad, 5671034) with Tris-Glycine buffer (Bio-Rad, 1610732). The affinity capture pulldown efficiency was monitored using in-gel fluorescence. The msGFP signal from HaloTag-TEV-4×GS-msGFP-mito after cell lysis and SDS-PAGE was analyzed, in-gel, using a Bio-Rad Chemidoc MP imager (Bio-Rad) through GFP fluorescence excitation and emission filters. Data were quantified by densitometry using Fiji.[14] Capture efficiency was calculated comparing the total GFP signal recovered from StrepTactin beads vs the signal recovered from the high speed spin of the bead supernatant and pooled wash volumes.

Quantification of HaloTag labeling efficiency using in-gel fluorescence. HEK293T cells were cultured, incubated and transfected with pF(UG) hSyn HaloTag-TEV-4×GS-msGFP-mito using lipofectamine LTX reagent as described above. To examine saturation of HaloTag, we screened for fluorescence plateau using 1 µM of JF_{479}-HaloTag ligand,[4] JF_{549}-HaloTag ligand ($\mathbf{2_{HTL}}$) and JF_{608}-HaloTag ligand ($\mathbf{3_{HTL}}$) at 15, 30, 60, 90, 120, and 150 minutes. Only JF_{549}-HaloTag ligand

(**2**$_{\mathbf{HTL}}$) and JF_{608}-HaloTag ligand (**3**$_{\mathbf{HTL}}$) reached a fluorescence plateau, and they were chosen as saturating counter stains or "chase" dyes for examining the labeling efficiency of biotin-JF-HaloTag ligands (**13**$_{\mathbf{HTL}}$–**16**$_{\mathbf{HTL}}$). Next, we developed an in-gel assay to independently quantitate labeling efficiencies of **13**$_{\mathbf{HTL}}$–**16**$_{\mathbf{HTL}}$. We used JF_{549}-HaloTag ligand (**2**$_{\mathbf{HTL}}$) as chase for evaluating biotin-JF_{608}-HaloTag ligand (**14**$_{\mathbf{HTL}}$), biotin-JF_{646}-HaloTag ligand (**15**$_{\mathbf{HTL}}$), and biotin-JF_{635}-HaloTag ligand (**16**$_{\mathbf{HTL}}$). We used JF_{608}-HaloTag ligand (**3**$_{\mathbf{HTL}}$) as chase for evaluating biotin-JF_{549}-HaloTag ligand (**13**$_{\mathbf{HTL}}$). Each experiment consisted of testing the biotin variants **13**$_{\mathbf{HTL}}$–**16**$_{\mathbf{HTL}}$ at 100 nM and 1 μM with an incubation time of 2 h and a chase labeling incubation of 1 μM for 0.5 h. The 0.5 h chase labeling period was included with the 2 h total incubation. Each experiment consisted of the following conditions: untransfected cells; (A) transduced, but no dye; (B) non-biotinylated variant of primary label at 1 μM for 0.5 h; (C) the nonbiotinylated version of the chase JF dye at 1 μM for 0.5 h; (D) 100 nM of the nonbiotinylated version of the primary label for 2 h; (E) 100 nM of the biotinylated version of primary label for 2 h; (F) 1 μM of the nonbiotinylated version of the primary label for 2 h; (G) 1 μM of the biotinylated version of primary label for 2 h; and (H) 1 μM of the non-biotinylated version of the chase JF dye at 1 μM for 2 h. This last condition H was the same as condition C but with a longer incubation (2 h *vs.* 0.5 h) to allow independent verification of the chase saturation using 0.5 h incubation. Samples D–G were incubated with a nonbiotinylated chase dye at 1 μM for 0.5 h. Cells were washed and protein lysates were harvested using Bio-Rad SDS-PAGE products as described above. Specifically, cells were lysed using protein lysis buffer as described above, and adding XT Sample Buffer (Bio-Rad, 1610791) and XT Reducing Agent (Bio-Rad, 1610792). Samples were heated at 72 ºC for 5 min. Samples were then loaded onto a 4–12% Criterion XT Bis-Tris gel (Bio-Rad, 3450124) and ran at 150 V for approximately 1 h. Gels were then imaged on a Chemidoc MP imager (Bio-Rad) using orange, red, and far-red filters. Bands representing HaloTag ligand were quantified by densitometry using Fiji.[14] These data were collected and analyzed in Microsoft Excel and GraphPad Prism. For labeling efficiency calculations, bands representing saturating chase dye fluorescence (*e.g.*, JF_{549}) were compared to saturating primary dye fluorescence allowing estimation of the amount of HaloTag that did not react to a primary dye during their initial incubation using equation 3:

$$F = 1 - \frac{C_e}{C_s} \tag{3}$$

Where F is the fraction of HaloTag protein labeled, C_e is the experimental chase dye fluorescence signal after incubation with pulse biotin-JF-HaloTag ligand, and C_s is the saturating chase dye fluorescence without incubation with a pulse ligand.

Neuro2a cell culture, labeling, lattice light sheet microscopy (LLSM), and confocal microscopy. Neuro2a cells (ATCC, CCL-131) were cultured in Eagle's minimum essential medium (MEM, phenol red-free; Life Technologies) supplemented with 10% (v/v) fetal bovine serum (FBS), and maintained at 37 °C in a humidified 5% (v/v) CO_2 environment. Cells were transiently transfected using nucleofection (Lonza) with plasmids constitutively expressing

Histone2B–mCherry, BRD4–sfGFP, and either coilin–HaloTag or heterochromatin protein 1a (HP1a)–HaloTag. All inserts cDNAs were synthesized by Genscript and subcloned into the EcoRI and NotI sites of the pCIG2 expression vector. 18–24 h post-transfection, the transiently transfected cells seeded on 5-mm coverslips were mounted in custom-fabricated sample holders and loaded on an LLSM built by Intelligent Imaging Innovations. Prior to imaging, the medium in the LLSM sample chamber was equilibrated with 8 nM (*i.e.*, 1/10 of the IC_{50} of free (*S*)-JQ1) or 20 nM (*i.e.*, 1/4 of the IC_{50} of free (*S*)-JQ1) of $\mathbf{18_{HTL}}$, $\mathbf{19_{HTL}}$, or $\mathbf{20_{HTL}}$. Full 3D volumes at multiple stage positions were collected at 2 minute intervals for 90 minutes total. To assess the locations of transcriptionally active chromatin, H2B–mCherry was replaced by histone H3.3–SNAP-tag, which was labeled by incubation with 1 μM JFX_{554}-SNAP-tag ligand ($\mathbf{21_{STL}}$) added at the same time as the transfection reagents; these cells were washed before imaging. The recruitment of histone H3.3 was assessed by imaging after 24 h incubation with $\mathbf{19_{HTL}}$. To assess the effect of free (*S*)-JQ1 on protein translocation, 25 μM of (*S*)-JQ1 was added with 20 nM $\mathbf{19_{HTL}}$ and 30 μm z-stacks were collected at 2 minute intervals for 90 minutes total using a Marianas Spinning Disk confocal microscope; identical timing to the LLSM experiments.

LLSM imaging processing. Raw image data volumes were deskewed and rotated, then empirically deconvolved using a Richardson-Lucy maximum likelihood algorithm using experimentally measured PSFs for each color channel for 30 iterations. Channel alignment corrections were performed using Advanced Normalization Tools R (ANTsR) registration run in "translation" mode using 2σ Gaussian-blurred versions of the images, and then transforms were applied to the deconvolved images. Next, whole nuclei were segmented using thresholds greater than 2–3× the noise floor. These segmentation masks were then used to isolate each nucleus for further quantitative analyses. For instance, HaloTag labeled domains were next defined by taking the signal intensity greater than 2× the median of the total signal intensity of HP1a within each nucleus. The amount of BRD4 signal was then assessed in- versus outside HaloTag labeled domains throughout the 90-min time-lapse acquisition. For visualization improvements of the time-lapse volumes, we used the photobleach correction ImageJ plugin run in histogram matching mode to correct photobleaching of the H2B–mCherry channel. In addition, we used ANTsR to register all subsequent timepoints of each nuclei to its initial timepoint position.

GENERAL SYNTHETIC ORGANIC CHEMISTRY METHODS

Commercial reagents were the highest quality available and used as received. Solvents for reactions were of anhydrous grade, purchased in septum-sealed bottles, and stored under an inert atmosphere. Reactions were conducted in round-bottomed flasks or septum-sealed crimp-top microwave reaction vials (Biotage) containing Teflon-coated magnetic stir bars. All reactions were conducted under an inert atmosphere of Ar(g) and protected from light using Al foil unless otherwise noted. Heating of reaction mixtures was achieved through aluminum blocks on top of a stirring hotplate equipped with an electronic contact thermometer. Reactions were monitored either by thin layer chromatography (TLC) on precoated TLC glass plates (silica gel 60 F254, 250 µm thickness) or by tandem liquid chromatography–mass spectrometry (LC–MS; Shimadzu LCMS 2020, Phenomenex Kinetex 30 × 2.1 mm 2.6 µm C18 column, 1–10 µL injection, 5–98% CH_3CN/H_2O linear gradient with constant 0.1% v/v HCO_2H, 6 min run, 1 mL/min flowrate, ESI, positive ion mode). TLC plates were visualized either by UV illumination or by developing the TLC with ceric ammonium molybdate or $KMnO_4$.

Reaction products were purified either by flash chromatography on Biotage Isolera automated purification system using prepacked silica gel columns and/or by preparative high-pressure liquid chromatography (HPLC; Agilent 1200, Phenomenex Gemini–NX 150 × 30 mm 10 µm C18 110 Å column, 42 mL/min flowrate) under the indicated solvent gradient conditions. Analytical HPLC analyses were performed on an LC-MS system (Agilent 1200, Phenomenex Gemini–NX 150 × 4.6 mm 5 µm C18 110 Å column, 1 mL/min flowrate) or an analytical HPLC (Shimadzu UFLC, Phenomenex Gemini–NX 150 × 4.6 mm 5 µm C18 110 Å column, 1 mL/min flowrate) under the indicated conditions. All the HPLC systems are fitted with a diode array detector. High-resolution mass spectrometry (HMRS) was obtained by the High Resolution Mass Spectrometry Facility at the University of Iowa. NMR spectra were recorded on Bruker Avance 400 MHz spectrometer and processed through MestReNova. Deuterated solvents were used as purchased. ^{1}H and ^{13}C chemical shifts (δ) were referenced to TMS or residual solvent peaks. ^{19}F chemical shifts were referenced to $CFCl_3$. Data for ^{1}H NMR spectra are reported as follows: chemical shift (δ ppm), multiplicity (s = singlet, d = doublet, t = triplet, q = quartet, p = pentet (quintet), dd = doublet of doublets, dt = doublet of triplets, m = multiplet, br = broad signal), coupling constant (Hz), and integration. Data for ^{13}C NMR spectra are reported by chemical shift (δ ppm) with hydrogen multiplicity (C, CH, CH_2, CH_3) information obtained from DEPT spectra.

EXPERIMENTALS AND CHARACTERIZATION FOR ALL NEW COMPOUNDS

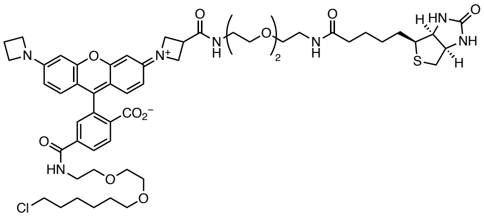

Biotin-JF$_{549}$-HaloTag ligand (13$_{HTL}$). The TFA salt of 3″-carboxy-JF$_{549}$-HaloTag ligand[16] (**8$_{HTL}$**; 10.0 mg, 8.5 μmol, 1 equiv) was dissolved in anhydrous DMF (2 mL). To this solution were added Et_3N (12.0 μL, 85 μmol, 10 equiv), DSC (5.5 mg, 21 μmol, 2.5 equiv), and a catalytic amount of DMAP (~0.05 mg). The reaction mixture was stirred for 90 min at ambient temperature, after which biotin-PEG$_2$-NH$_2$ (**12**, 32.0 mg, 85 μmol, 10 equiv) was added. The reaction mixture was further stirred for 16 h at ambient temperature. The solvent was removed under reduced pressure, and the product was purified by preparative HPLC using a 5–95% CH_3CN/H_2O linear gradient with constant 0.1% v/v trifluoroacetic acid (TFA). Product-containing fractions were combined and lyophilized to obtain **13$_{HTL}$** as a red solid (TFA salt, 4.5 mg, 51%). ^{1}H NMR (CD_3OD, 400 MHz) δ 8.39 (d, *J* = 8.2 Hz, 1H), 8.21 (dd, *J* = 8.2, 1.8 Hz, 1H), 7.81 (d, *J* = 1.8 Hz, 1H), 7.08 (d, *J* = 9.1 Hz, 2H), 6.63 (ddd, *J* = 9.2, 5.3, 2.1 Hz, 2H), 6.59 (d, *J* = 2.1 Hz, 1H), 6.53 (d, *J* = 2.1 Hz, 1H), 4.48 – 4.39 (m, 3H), 4.38 – 4.23 (m, 7H), 3.74 – 3.51 (m, 19H), 3.48 – 3.41 (m, 4H), 3.36 (t, *J* = 5.5 Hz, 2H), 3.22 – 3.14 (m, 1H), 2.89 (dd, *J* = 12.8, 5.0 Hz, 1H), 2.66 (dd, *J* = 12.8, 3.0 Hz, 1H), 2.56 (p, *J* = 7.4 Hz, 2H), 2.21 (t, *J* = 7.4 Hz, 2H), 1.77 – 1.55 (m, 6H), 1.53–1.47 (m, 2H), 1.45 – 1.30 (m, 6H). ^{13}C NMR (CD_3OD, 101 MHz) δ 176.12 (C), 173.82 (C), 167.92 (C), 167.34 (C), 166.03 (C), 160.59 (C), 158.93 (C), 158.63 (C), 158.18 (C), 157.68 (C), 139.40 (C), 135.58 (C), 134.75 (C), 132.84 (CH), 132.44 (CH), 132.33 (CH), 130.37 (CH), 130.05 (CH), 115.15 (C), 114.96 (C), 113.94 (CH), 113.49 (CH), 95.61 (CH), 95.18 (CH), 72.14 (CH_2), 71.31 (CH_2), 71.24 (CH_2), 71.14 (CH_2), 70.61 (CH_2), 70.46 (CH_2), 70.35 (CH_2), 63.33 (CH), 61.61 (CH), 56.97 (CH), 55.32 (CH_2), 52.98 (CH_2), 45.75 (CH_2), 41.21 (CH_2), 41.06 (CH_2), 40.63 (CH_2), 40.28 (CH_2), 36.74 (CH_2), 34.62 (CH), 33.72 (CH_2), 30.46 (CH_2), 29.76 (CH_2), 29.50 (CH_2), 27.69 (CH_2), 26.84 (CH_2), 26.44 (CH_2), 16.81 (CH_2). ^{19}F NMR (CD_3OD, 376 MHz) δ −75.35. Analytical HPLC: t_R= 11.2 min, 98.0% purity (10–95% MeCN/H_2O linear gradient over 20 min with constant 0.1% v/v TFA, 1 mL/min flow rate, detection at 550 nm). HRMS (ESI) calculated for $C_{54}H_{71}N_7O_{11}SCl$ $[M+H]^+$ = 1060.4615, found 1060.4623.

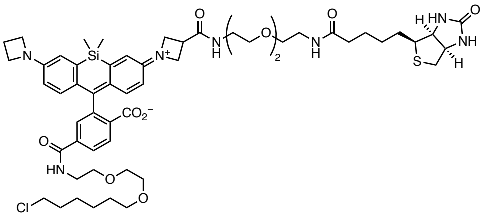

Biotin-JF$_{646}$-HaloTag ligand (15$_{HTL}$). The TFA salt of 3″-carboxy-JF$_{646}$ -HaloTag ligand[16] (**10$_{HTL}$**; 57.0 mg, 66 μmol, 1 equiv) was dissolved in DMF (2 mL). To this solution were added Et_3N (96.0 μL, 661 μmol, 10 equiv), DSC (42.3 mg, 165.2 μmol, 2.5 equiv), and a catalytic amount of DMAP (~0.05 mg). The reaction mixture was stirred for 90 min at ambient temperature, after which biotin-PEG$_2$-NH$_2$ (**12**, 99.0 mg, 264 μmol, 4 equiv) was added. The reaction mixture was stirred overnight at ambient temperature. The solvent was removed under reduced pressure, and the product was purified by preparative HPLC using a 30–50% CH_3CN/H_2O linear gradient with a constant 0.1% v/v TFA. Product-containing fractions were combined and lyophilized to obtain **15$_{HTL}$** as a blue solid (TFA salt, 43 mg, 58.1%). ^{1}H NMR (CD_3OD, 400 MHz) δ 8.24 (d, J = 8.2 Hz, 1H), 8.10 (dd, J = 8.2, 1.7 Hz, 1H), 7.69 (d, J = 1.6 Hz, 1H), 6.92 (d, J = 2.5 Hz, 2H), 6.88 (dd, J = 9.2, 7.9 Hz, 2H), 6.35 (dd, J = 9.2, 2.6 Hz, 2H), 4.47 – 4.42 (m, 1H), 4.41 – 4.18 (m, 9H), 3.67 – 3.49 (m, 19H), 3.45 – 3.40 (m, 4H), 3.35 (t, J = 5.5 Hz, 2H), 3.20 – 3.12 (m, 1H), 2.88 (dd, J = 12.8, 5.0 Hz, 1H), 2.66 (d, J = 12.7 Hz, 1H), 2.51 (p, J = 7.6 Hz, 2H), 2.20 (t, J = 7.4 Hz, 2H), 1.76 – 1.55 (m, 6H), 1.53 – 1.47 (m, 2H), 1.46 – 1.29 (m, 6H), 0.60 (s, 3H), 0.55 (s, 3H). ^{13}C NMR (CD_3OD, 400 MHz) δ 176.11 (C), 174.04 (C), 168.44 (C), 168.22 (C), 166.04 (C), 154.06 (C), 153.57 (C), 139.38 (C), 133.58 (C), 131.20 (CH), 130.41 (CH), 130.25 (CH), 129.00 (CH), 119.57 (CH), 118.98 (CH), 113.39 (CH), 113.08 (CH), 72.12 (CH_2), 71.30 (CH_2), 71.20 (CH_2), 71.13 (CH_2), 70.61 (CH_2), 70.47 (CH_2), 70.36 (CH_2), 63.34 (CH), 61.60 (CH), 56.97 (CH), 55.39 (CH_2), 53.32 (CH_2), 45.74 (CH_2), 41.14 (CH_2), 41.05 (CH_2), 40.59 (CH_2), 40.27 (CH_2), 36.74 (CH_2), 34.90 (CH), 33.71 (CH_2), 30.44 (CH_2), 29.75 (CH_2), 29.49 (CH_2), 27.68 (CH_2), 26.83 (CH_2), 26.42 (CH_2), 17.04 (CH_2), −0.74 (CH_3), −1.66 (CH_3); five aromatic quaternary carbons were not observed. ^{19}F NMR (CD_3OD, 376 MHz) δ −75.35. Analytical HPLC: t_R= 11.9 min, 97.0% purity (10–95% MeCN/H_2O linear gradient over 20 min with constant 0.1% v/v TFA, 1 mL/min flow rate, detection at 254 nm). HRMS (ESI) calculated for $C_{56}H_{76}N_7O_{10}SClSiNa$ $[M+Na]^+$ = 1124.4724**,** found 1124.4730.

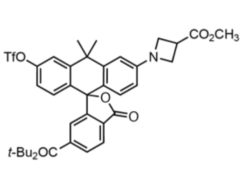

methyl 1-(6'-(*tert*-butoxycarbonyl)-10,10-dimethyl-3'-oxo-3-(((trifluoromethyl)sulfonyl)oxy) -3'*H*,10*H*-spiro[anthracene-9,1'-isobenzofuran]-6-yl)azetidine-3-carboxylate (S3). An oven-dried microwave reaction vial was charged with 6-*tert*-butoxycarbonyl carbofluorescein ditriflate[2] (**S1**, 722.6 mg, 1 mmol, 1 equiv), Pd_2dba_3 (46 mg, 50 μmol, 0.05 equiv), XPhos (72 mg, 150 μmol, 0.15 equiv), methyl azetidine-3-carboxylate hydrochloride (**S2**, 182 mg, 1.2 mmol, 1.2 equiv), and Cs_2CO_3 (912 mg, 2.8 mmol, 2.8 equiv). The vial was sealed and backfilled with Ar(g) (3×), after which anhydrous dioxane (10 mL) was added. The resulting mixture was stirred at 80 °C for 3 h. The reaction was cooled and filtered through a pad of celite using EtOAc. The filtrate was

concentrated under reduced pressure. Purification by SiO_2 gel chromatography (50 g SiO_2 column, 0–50% EtOAc/hexanes, linear gradient) provided partially purified monoazetidine compound **S3** as a light green foamy solid (220 mg) and unreacted starting material (168 mg). LC–MS (ESI): calculated for $C_{34}H_{33}F_3NO_9S$ $[M+H]^+$ = 688.18, found 688.18. This material was used immediately in the subsequent synthetic step.

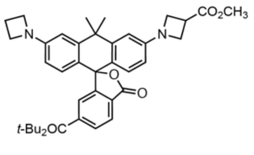

methyl 1-(3-(azetidin-1-yl)-6'-(*tert*-butoxycarbonyl)-10,10-dimethyl-3'-oxo-3'*H*,10*H*-spiro [anthracene-9,1'-isobenzofuran]-6-yl)azetidine-3-carboxylate (*i.e.*, 3″-methoxycarbonyl-6-*tert*-butoxycarbonyl-JF$_{608}$; S5). An oven-dried microwave reaction vial was charged with **S3** (220 mg, 320 µmol, 1 equiv), Pd_2dba_3 (29 mg, 32 µmol, 0.1 equiv), XPhos (46 mg, 96 µmol, 0.3 equiv), azetidine (**S4**, 108 µL, 91 mg, 1.6 mmol, 5 equiv), and Cs_2CO_3 (292 mg, 896 µmol, 2.8 equiv). The vial was sealed and backfilled with Ar(g) (3×), after which anhydrous dioxane (10 mL) was added. The resulting mixture was stirred at 100 °C for 4 h, then cooled to ambient temperature and filtered through a pad of celite using EtOAc. The filtrate was concentrated under reduced pressure. Purification by SiO_2 gel chromatography (50 g SiO_2 column, 0–100% EtOAc/hexanes, linear gradient) provided **S5** as a green-blue solid (153 mg, 33.4% over two steps). ^{1}H NMR ($CDCl_3$, 400 MHz) δ 8.15 (dd, *J* = 8.0, 1.3 Hz, 1H), 8.01 (d, *J* = 8.0 Hz, 1H), 7.61 (t, *J* = 1.0 Hz, 1H), 6.63 – 6.51 (m, 4H), 6.23 (ddd, *J* = 8.8, 5.1, 2.3 Hz, 2H), 4.15 – 4.03 (m, 4H), 3.92 (t, *J* = 7.2 Hz, 4H), 3.75 (s, 3H), 3.58 (tt, *J* = 8.6, 6.1 Hz, 1H), 2.38 (p, *J* = 7.2 Hz, 2H), 1.83 (s, 3H), 1.73 (s, 3H), 1.53 (s, 9H). ^{13}C NMR (101 MHz, $CDCl_3$) δ 173.21 (C), 170.08 (C), 164.51 (C), 155.51 (C), 152.37 (C), 151.63 (C), 146.92 (C), 146.67 (C), 137.85 (C), 130.17 (CH), 130.13 (C), 128.99 (CH), 128.91 (CH), 125.05 (CH), 124.87 (CH), 120.85 (C), 119.77 (C), 110.77 (CH), 110.62 (CH), 108.43 (CH), 108.05 (CH), 82.36 (C), 54.55 (CH_2), 54.52 (CH_2), 52.38 (CH_3), 52.36 (CH_2), 38.49 (C), 35.43 (CH_3), 33.54 (CH_3), 32.81 (CH), 28.16 (CH_3), 16.94 (CH_2). HRMS (ESI) calculated for $C_{36}H_{39}N_2O_6$ $[M+H]^+$ = 595.2803, found 595.2804.

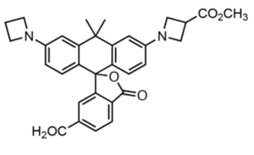

6-carboxy-3″-methoxycarbonyl-JF$_{608}$ (S6**).** Compound **S5** (32 mg, 54 µmol) was dissolved in CH_2Cl_2 (2 mL). To this solution was added TFA (0.4 mL), and the resulting blue solution was stirred at ambient temperature for 7 h. The reaction was concentrated under reduced pressure,

yielding **S6** as a blue solid. HRMS (ESI): calculated for $C_{32}H_{31}N_2O_6$ $[M+H]^+$ = 539.2177, found 539.2182. This material was used immediately in the subsequent synthetic step.

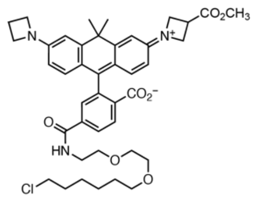

3″-methoxycarbonyl-JF$_{608}$ -HaloTag ligand (S8). Compound **S6** was dissolved in DMF (2 mL). To this solution were added DIEA (96 µL, 538 µmol, 10 equiv) and TSTU (24 mg, 81 µmol, 1.5 equiv). The reaction mixture was stirred for 10 min at ambient temperature, after which HaloTag(O_2)-NH_2 (**S7**, 21 mg, 81 µmol, 1.5 equiv) was added. The reaction mixture was stirred for 16 h at ambient temperature. The solvent was removed under reduced pressure to yield **S8** as a blue solid. LC–MS (ESI) calculated for $C_{42}H_{51}ClN_3O_7$ $[M+H]^+$ = 744.34, found 744.40. This material was used immediately in the subsequent synthetic step.

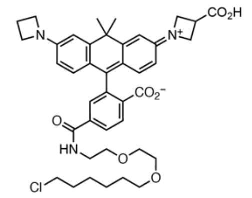

3″-carboxy-JF$_{608}$ -HaloTag ligand (9_{HTL}**).** NaOH (1 M, 108 µL) was added to a solution of **S8** in CH_3OH (4 mL). The resulting solution was stirred for 14 h. The reaction mixture was acidified to pH ~1 using 1M HCl. This crude product was purified by preparative HPLC using a 25–75% CH_3CN/H_2O linear gradient with a constant 0.1% v/v TFA. Product-containing fractions were combined and lyophilized to obtain **9_{HTL}** as a blue solid (TFA salt, 19 mg, 41.8% over three steps). ^{1}H NMR ($CDCl_3$, 400 MHz) δ 8.33 (d, *J* = 8.3 Hz, 1H), 8.15 (dd, *J* = 8.2, 1.8 Hz, 1H), 7.73 (d, *J* = 1.8 Hz, 1H), 6.94 (t, *J* = 9.0 Hz, 2H), 6.87 (d, *J* = 2.3 Hz, 1H), 6.84 (d, *J* = 2.2 Hz, 1H), 6.41 (t, *J* = 2.1 Hz, 1H), 6.39 (t, *J* = 2.1 Hz, 1H), 4.47 (t, *J* = 9.5 Hz, 2H), 4.41 – 4.30 (m, 6H), 3.72 – 3.67 (m, 1H), 3.66 – 3.59 (m, 5H), 3.58 – 3.55 (m, 3H), 3.52 (t, *J* = 6.6 Hz, 2H), 3.43 (t, *J* = 6.5 Hz, 2H), 2.55 (p, *J* = 7.6 Hz, 2H), 1.82 (s, 3H), 1.75 – 1.68 (m, 5H), 1.54 – 1.46 (m, 2H), 1.44 – 1.37 (m, 2H), 1.37 – 1.30 (m, 2H). ^{13}C NMR (101 MHz, $CDCl_3$) δ 175.37 (C), 168.10 (C), 167.62 (C), 158.20 (C), 157.25 (C), 156.98 (C), 156.31 (C), 140.23 (C), 139.03 (C), 137.85 (CH), 137.16 (CH), 134.71 (C), 132.24 (CH), 129.97 (CH), 129.35 (CH), 122.05 (C), 121.98 (C), 112.26 (CH), 111.82 (CH), 110.00 (CH), 109.79 (CH), 72.12 (CH_2), 71.20 (CH_2), 71.13 (CH_2), 70.36 (CH_2), 55.25 (CH_2), 53.06 (CH_2), 45.73 (CH_2), 42.70 (C), 41.15 (CH_2), 35.36 (CH_3), 33.91 (CH), 33.71 (CH_2),

32.27 (CH_3), 30.44 (CH_2), 27.68 (CH_2), 26.43 (CH_2), 16.83 (CH_2). HRMS (ESI) calculated for $C_{41}H_{49}ClN_3O_7$ $[M+H]^+$ = 730.3254, found 730.3255.

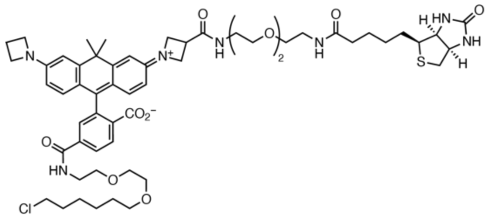

Biotin-JF$_{608}$-HaloTag ligand (14$_{HTL}$). The TFA salt of **9$_{HTL}$** (19 mg, 22.5 μmol, 1 equiv) was dissolved in DMF (3 mL). To this solution were added DIEA (40 μL, 225 μmol, 10 equiv), biotin-PEG$_2$-NH$_2$ (**12**, 9 mg, 33.7 μmol, 1.5 equiv), and HATU (12 mg, 33.8 μmol, 1.5 equiv). The reaction mixture was stirred for 18 h at ambient temperature. The solvent was removed under reduced pressure, and the product was purified by preparative HPLC using a 30–60% CH_3CN/H_2O linear gradient with a constant 0.1% v/v TFA. Product-containing fractions were combined and lyophilized to obtain **14$_{HTL}$** as a blue solid (TFA salt, 19.8 mg, 73.3%). ^{1}H NMR (CD_3OD, 400 MHz) δ 8.34 (d, *J* = 8.3 Hz, 1H), 8.15 (dd, *J* = 8.3, 1.8 Hz, 1H), 7.75 (d, *J* = 1.8 Hz, 1H), 6.97 (d, *J* = 6.8 Hz, 1H), 6.95 (d, *J* = 6.8 Hz, 1H), 6.87 (d, *J* = 2.2 Hz, 1H), 6.84 (d, *J* = 2.2 Hz, 1H), 6.42 (t, *J* = 2.4 Hz, 1H), 6.39 (t, *J* = 2.5 Hz, 1H), 4.50 – 4.31 (m, 9H), 4.27 (dd, *J* = 7.9, 4.4 Hz, 1H), 3.67 – 3.51 (m, 19H), 3.43 (t, *J* = 6.4 Hz, 4H), 3.36 (t, *J* = 5.5 Hz, 2H), 3.18 (dt, *J* = 8.9, 5.3 Hz, 1H), 2.89 (ddd, *J* = 12.8, 5.0, 1.0 Hz, 1H), 2.66 (dt, *J* = 12.8, 1.2 Hz, 1H), 2.56 (p, *J* = 7.6 Hz, 2H), 2.21 (t, *J* = 7.3 Hz, 2H), 1.82 (s, 3H), 1.73– 1.63 (m, 6H), 1.67 – 1.56 (m, 3H), 1.51 – 1.47 (m, 2H), 1.46 – 1.31 (m, 6H). ^{13}C NMR (CD_3OD, 101 MHz) δ 176.13 (C), 173.93 (C), 168.08 (C), 167.44 (C), 166.05 (C), 158.51 (C), 157.65 (C), 157.03 (C), 156.37 (C), 139.48 (C), 138.91 (C), 138.11 (CH), 137.51 (CH), 134.86 (C), 132.53 (CH), 130.26 (CH), 129.33 (CH), 122.03 (C), 121.99 (C), 112.21 (CH), 111.83 (CH), 109.98 (CH), 109.78 (CH), 72.12 (CH_2), 71.30 (CH_2), 71.20 (CH_2), 71.13 (CH_2), 70.60 (CH_2), 70.45 (CH_2), 70.36 (CH_2), 63.33 (CH), 61.60 (CH), 56.99 (CH), 55.24 (CH_2), 53.02 (CH_2), 45.75 (CH_2), 42.83 (C), 41.15 (CH_2), 41.06 (CH_2), 40.61 (CH_2), 40.27 (CH_2), 36.73 (CH_2), 35.40 (CH_3), 34.63 (CH_3), 33.71 (CH_2), 32.23 (CH), 30.45 (CH_2), 29.77 (CH_2), 29.50 (CH_2), 27.68 (CH_2), 26.84 (CH_2), 26.43 (CH_2), 16.80 (CH_2). ^{19}F NMR (CD_3OD, 376 MHz) δ −75.45. HRMS (ESI) calculated for $C_{57}H_{77}N_7O_{10}SCl$ $[M+H]^+$ = 1086.5136, found 1086.5147.

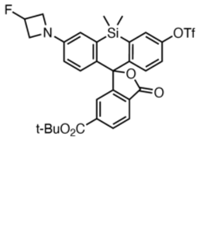

***tert*-butyl 3-(3-fluoroazetidin-1-yl)-5,5-dimethyl-3'-oxo-7-(((trifluoromethyl)sulfonyl)oxy)-3'*H*,5*H*-spiro[dibenzo[*b*,*e*]siline-10,1'-isobenzofuran]-6'-carboxylate** (S11**).** An oven-dried microwave reaction vial was charged with 6-*tert*-butoxycarbonyl-Si-fluorescein ditriflate[2] (**S9**, 370 mg, 500 µmol, 1 equiv), Pd_2dba_3 (46 mg, 50 µmol, 0.1 equiv), XantPhos (87 mg, 150 µmol, 0.3 equiv), 3-fluoroazetidine hydrochloride (**S10**, 62 mg, 550 µmol, 1.1 equiv), and Cs_2CO_3 (381 mg, 1.17 mmol, 2.4 equiv). The vial was sealed and backfilled with Ar(g) (3×), after which anhydrous dioxane (3 mL) was added. The resulting mixture was stirred at 80 °C for 2.5 h. The reaction was cooled and filtered through a pad of celite using CH_2Cl_2. The filtrate was concentrated under reduced pressure. Purification by SiO_2 gel chromatography (50 g SiO_2 column, 0–25% EtOAc/hexanes, linear gradient) provided monoazetidine compound **S11** as a light-green foamy solid (210 mg). LC–MS (ESI): calculated for $C_{31}H_{29}F_4NO_7SSi$ $[M+H]^+$ = 663.14, found 664.15. This material was used immediately in the subsequent synthetic step.

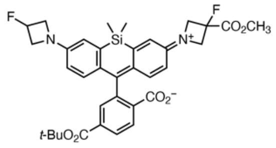

4-(*tert*-butoxycarbonyl)-2-(3-(3-fluoro-3-(methoxycarbonyl)azetidin-1-ium-1-ylidene)-7-(3-fluoroazetidin-1-yl)-5,5-dimethyl-3,5-dihydrodibenzo[*b*,*e*]silin-10-yl)benzoate (*i.e.*, 3″-methoxycarbonyl-6-*tert*-butoxycarbonyl-JF$_{635}$; S13**).** An oven-dried microwave reaction vial was charged with **S11** (210 mg, 316 µmol, 1 equiv), Pd_2dba_3 (29 mg, 32 µmol, 0.1 equiv), XPhos (55 mg, 95 µmol, 0.3 equiv), methyl 3-fluoroazetidine-3-carboxylate hydrochloride (**S12,** 161 mg, 949 µmol, 3 equiv), and Cs_2CO_3 (495 mg, 1.52 mmol, 4.8 equiv). The vial was sealed and backfilled with Ar(g) (3×), after which anhydrous dioxane (4 mL) was added. The resulting mixture was stirred at 100 °C for 4 h, then cooled to ambient temperature and filtered through a pad of celite using CH_2Cl_2. The filtrate was concentrated under reduced pressure. Purification by SiO_2 gel chromatography (50 g SiO_2 column, 0–40% EtOAc/hexanes, linear gradient) provided **S13** as a light green solid (177 mg, 55% over two steps). ^{1}H NMR ($CDCl_3$, 400 MHz) δ 8.13 (dd, J = 8.0, 1.3 Hz, 1H), 7.98 (dd, J = 8.0, 0.7 Hz, 1H), 7.83 (t, J = 1.0 Hz, 1H), 6.91 (dd, J = 8.7, 7.2 Hz, 2H), 6.72 (dd, J = 5.6, 2.7 Hz, 2H), 6.36 (ddd, J = 8.7, 5.0, 2.7 Hz, 2H), 5.46 – 5.46 (m, 0.5H), 5.36 – 5.31 (m, 0.5H), 4.42 – 4.34 (m, 2H), 4.24 – 4.13 (m, 4H), 4.04 – 4.00 (m, 1H), 3.98 – 3.94 (m, 1H), 3.87 (s, 3H), 1.55 (s, 9H), 0.68 (s, 3H), 0.60 (s, 3H). ^{13}C NMR (101 MHz, $CDCl_3$) δ 170.11 (C), 168.67 (d, $^2J_{CF}$ = 28.0 Hz, C), 164.32 (C), 155.11 (C), 149.90 (C), 149.05 (C), 137.37 (C), 136.40 (C), 136.16 (C), 133.99 (C), 133.37 (C), 130.07 (CH), 128.89 (C), 127.75 (CH), 127.73 (CH), 125.79 (CH), 125.00 (CH), 116.28 (CH), 116.23 (CH), 113.35 (CH), 113.22 (CH), 91.21 (C), 88.28 (d, $^1J_{CF}$ = 219.9 Hz, C), 82.76 (d, $^1J_{CF}$ = 204.9 Hz, CH), 82.43 (C), 61.22 (d, $^2J_{CF}$ = 25.1 Hz, CH_2), 59.55 (d, $^2J_{CF}$ = 23.8 Hz, CH_2), 53.25 (CH_3), 28.19 (CH_3), 0.11 (CH_3), −0.69 (CH_3). HRMS (ESI) calculated for $C_{35}H_{37}F_2N_2O_6Si$ $[M+H]^+$ = 647.2383, found 647.2376.

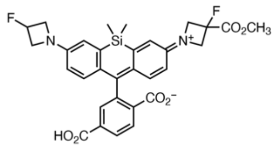

6-carboxy-3″-methoxycarbonyl-JF$_{635}$ (S14). Compound **S13** (16 mg, 25 µmol, 1 equiv) was dissolved in CH_2Cl_2 (2 mL). To this solution was added TFA (0.4 mL), and the resulting blue solution was stirred at ambient temperature for 4 h. The reaction was concentrated under reduced pressure, yielding **S14** as a blue solid. LC–MS (ESI): calculated for $C_{31}H_{29}F_2N_2O_6Si$ $[M]^+$ = 591.18, found 591.25. This material was used immediately in the subsequent synthetic step.

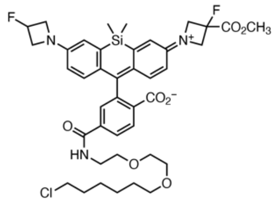

3″-methoxycarbonyl-JF$_{635}$-HaloTag ligand (S15). Compound **S14** was dissolved in DMF (2 mL). To this solution were added *N*,*N*-Diisopropylethylamine (DIEA; 43 µL, 247 µmol, 10 equiv) and *N*,*N*,*N′*,*N′*-tetramethyl-*O*-(N-succinimidyl)uronium tetrafluoroborate (TSTU; 12.3 mg, 41 µmol, 1.65 equiv). The reaction mixture was stirred for 10 min at ambient temperature, after which HaloTag(O_2)-NH_2 (**S7**, 8 mg, 31 µmol, 1.25 equiv) was added. The reaction mixture was stirred for 16 h at ambient temperature. The solvent was removed under reduced pressure to yield **S15** as a blue solid. LC–MS (ESI) calculated for $C_{41}H_{49}ClF_2N_3O_7Si$ $[M]^+$ = 796.30, found 796.20. This material was used immediately in the subsequent synthetic step.

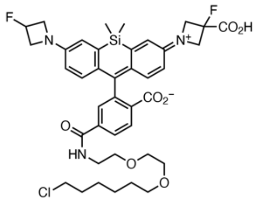

3″-carboxy-JF$_{635}$ -HaloTag ligand (11$_{HTL}$). Compound **S15** was dissolved in a mixture of THF:CH_3OH (1:1 v/v; 4 mL). An aqueous solution of NaOH (1 M, 266 µL) was added, and the resulting solution and stirred for 7 h. The reaction mixture was diluted with H_2O (2 mL) and acidified to pH ~1 using an aqueous solution of HCl (1 M). The mixture was extracted with EtOAc (4×2 mL), and the combined organics were concentrated under reduced pressure. This crude product was purified by preparative HPLC using a 5–95% CH_3CN/H_2O linear gradient with a

constant 0.1% v/v TFA. Product-containing fractions were combined and lyophilized to obtain **11** as a blue solid (TFA salt, 13.6 mg, 61% over three steps). ^{1}H NMR ($CDCl_3$, 400 MHz) δ 8.02–8.0 (m, 1H), 7.92 – 7.90 (m, 1H), 7.72 (s, 1H), 7.20 (t, J = 1.0 Hz, 1H), 6.80 (dd, J = 8.7, 1.4 Hz, 2H), 6.72 (dd, J = 12.6, 2.6 Hz, 2H), 6.62 (d, J = 2.6 Hz, 1H), 6.31 (dd, J = 8.8, 2.6 Hz, 1H), 6.26 (dd, J = 8.9, 2.6 Hz, 1H), 5.52 – 5.49 (m, 0.5H), 5.35–5.33 (m, 0.5H), 4.39 – 4.24 (m, 4H), 4.18 – 4.01 (m, 4H), 3.64 – 3.62 (m, 6H), 3.58 – 3.55 (m, 2H), 3.49 (t, J = 6.6 Hz, 2H), 3.42 (t, J = 6.7 Hz, 2H), 1.75–1.67 (m, 2H), 1.44 (p, J = 6.9 Hz, 2H), 1.43 – 1.35 (m, 2H), 1.33 – 1.27 (m, 2H), 0.53 (s, 3H), 0.46 (s, 3H). ^{13}C NMR (101 MHz, $CDCl_3$) δ 169.99 (d, $^{2}J_{CF}$ = 28.3 Hz, C), 169.44 (C), 167.06 (C), 152.36 (C), 150.32 (C), 149.73 (C), 139.34 (C), 138.98 (C), 138.85 (C), 132.95 (C), 132.62 (C), 130.27 (C), 129.77 (C), 127.77 (CH), 127.20 (CH), 124.83 (CH), 117.29 (CH), 116.92 (CH), 113.42 (CH), 113.03 (CH), 87.73 (d, $^{1}J_{CF}$ = 220.4 Hz, C), 82.49 (d, $^{1}J_{CF}$ = 205.0 Hz, CH), 71.40 (CH_2), 70.22 (CH_2), 69.99 (CH_2), 69.48 (CH_2), 61.29 (d, $^{2}J_{CF}$ = 25.2 Hz, CH_2), 59.74 (d, $^{2}J_{CF}$ = 24.9 Hz, CH_2), 45.14 (CH_2), 40.35 (CH_2), 32.57 (CH_2), 29.35 (CH_2), 26.72 (CH_2), 25.41 (CH_2), 0.13 (CH_3), −1.43 (CH_3); one aromatic quaternary carbon was not observed. HRMS (ESI) calculated for $C_{40}H_{47}ClF_2N_3O_7Si$ $[M+H]^+$ = 782.2834, found 782.2823.

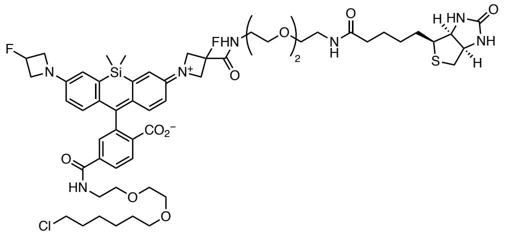

Biotin-JF$_{635}$-HaloTag ligand (16$_{HTL}$). The TFA salt of **11$_{HTL}$** (61.9 mg, 69 µmol, 1 equiv) was dissolved in DMF (2 mL). To this solution were added DIEA (121 µL, 690 µmol, 10 equiv), *N*-(3-dimethylaminopropyl)-*N'*-ethylcarbodiimide hydrochloride (EDC·HCl; 17.2 mg, 90 µmol, 1.3 equiv), and 1-[bis(dimethylamino)methylene]-1*H*-1,2,3-triazolo[4,5-*b*]pyridinium 3-oxid hexafluorophosphate (HATU; 34.2 mg, 90 µmol, 1.3 equiv). The reaction mixture was stirred at ambient temperature for 10 min, after which biotin-PEG$_2$-NH_2 (**12**, 27.4 mg, 104 µmol, 1.5 equiv) was added. The reaction mixture was stirred for a further 16 h at ambient temperature. The solvent was removed under reduced pressure, and the product was purified by preparative HPLC using a 40–55% CH_3CN/H_2O linear gradient with a constant 0.1% v/v TFA. Product-containing fractions were combined and lyophilized to obtain **16$_{HTL}$** as a blue solid (TFA salt, 42.4 mg, 48.9%). ^{1}H NMR (CD_3OD, 400 MHz) δ 7.97 (q, J = 8.0 Hz, 2H), 7.59 (d, J = 1.6 Hz, 1H), 6.78 (dd, J = 14.7, 2.6 Hz, 2H), 6.72 (dd, J = 8.9, 4.7 Hz, 2H), 6.30 (ddd, J = 11.9, 8.9, 2.6 Hz, 2H), 5.40 (tt, J = 6.1, 3.3 Hz, 0.5H), 5.26 (tt, J = 6.1, 3.3 Hz, 1H), 4.38 – 4.25 (m, 3H), 4.25 – 4.10 (m, 5H), 4.05 – 3.88 (m, 2H), 3.54 – 3.34 (m, 19H), 3.29 (t, J = 6.5 Hz, 2H), 3.25 (t, J = 5.5 Hz, 2H), 3.02 (td, J = 8.9, 4.1 Hz, 1H), 2.73 (dt, J = 12.8, 4.7 Hz, 1H), 2.54 (dd, J = 12.7, 1.9 Hz, 1H), 2.09 (t, J = 7.4 Hz, 2H), 1.64 – 1.41 (m, 6H), 1.40 – 1.35 (m, 2H), 1.33 – 1.15 (m, 6H), 0.55 (s, 3H), 0.46 (s, 3H). ^{13}C NMR (CD_3OD, 101 MHz, 320 K) δ 176.07 (C), 171.56 (C), 170.37 (d, $^{2}J_{CF}$ = 24.0 Hz, C),

168.66 (C), 165.98 (C), 152.01 (C), 151.24 (C), 141.72 (C), 138.96 (C), 134.26 (C), 133.81 (C), 129.76 (C), 129.36 (CH), 127.01 (CH), 124.76 (CH), 117.54 (CH), 114.38 (CH), 92.34 (d, $^1J_{CF}$ = 221.4 Hz, C), 84.29 (d, $^1J_{CF}$ = 202.7 Hz, CH), 72.15 (CH_2), 71.34 (CH_2), 71.32 (CH_2), 71.26 (CH_2), 71.14 (CH_2), 70.68 (CH_2), 70.36 (CH_2), 70.29 (CH_2), 63.36 (CH), 62.29 (d, $^2J_{CF}$ = 25.0 Hz, CH_2), 61.63 (CH), 60.59 (d, $^2J_{CF}$ = 24.1 Hz, CH_2), 56.90 (CH), 45.67 (CH_2), 41.17 (CH_2), 40.98 (CH_2), 40.35 (CH_2), 40.33 (CH_2), 36.77 (CH_2), 33.69 (CH_2), 30.41 (CH_2), 29.73 (CH_2), 29.48 (CH_2), 27.65 (CH_2), 26.77 (CH_2), 26.39 (CH_2), −0.14 (CH_3), −1.19 (CH_3); two aromatic quaternary carbons were not observed. ^{19}F NMR (CD_3OD, 376 MHz) δ −75.55, −161.83, −180.00. Analytical HPLC: t_R= 11.6 min, 99.1% purity (5–95% MeCN/H_2O linear gradient over 15 min with constant 0.1% v/v TFA, 1 mL/min flow rate, detection at 254 nm). HRMS (ESI) calculated for $C_{56}H_{75}N_7O_{10}F_2SClSiNa$ $[M+Na]^+$ = 1160.4536, found 1160.4545.

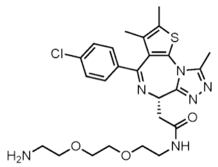

(*S*)-JQ1-PEG$_2$-amine (S19). (+)-JQ1-CO_2H (**S16**, 200 mg, 0.5 mmol, 1 equiv) was dissolved in DMF (4 mL). To this solution were added DIEA (448 µL, 1.25 mmol, 5 equiv), HATU (284 mg, 0.75 mmol, 1.5 equiv), and *tert*-butyl (2-(2-(2-aminoethoxy)ethoxy)ethyl)carbamate (**S17**, 186 mg, 0.75 mmol, 1.5 equiv). The reaction mixture was stirred at ambient temperature for 16 h. The solvent was removed under reduced pressure and resuspended in DCM (8 mL). TFA (2.5 mL) was added, and the reaction mixture was stirred at ambient temperature for 8 h. Volatiles were removed under reduced pressure and the product was purified by preparative HPLC using a 30–95% CH_3CN/H_2O linear gradient with a constant 0.1% v/v TFA. Product-containing fractions were combined and lyophilized to obtain **S19** as a yellow gum (5×TFA salt, 198.8 mg, 47.9% over two steps). The molar equivalents of TFA were determined via ^{19}F NMR by using fluorobenzene as an internal standard. ^{1}H NMR (CD_3OD, 400 MHz) δ 7.49 – 7.46 (m, 2H), 7.45 – 7.40 (m, 2H), 4.72 (dd, *J* = 8.3, 6.0 Hz, 1H), 3.74 – 3.70 (m, 2H), 3.68 (s, 4H), 3.62 (t, *J* = 5.6 Hz, 2H), 3.54 – 3.34 (m, 4H), 3.13 (t, *J* = 5.0 Hz, 2H), 2.76 (s, 3H), 2.46 (s, 3H), 1.70 (s, 3H). ^{13}C NMR (CD_3OD, 101 MHz) δ 172.77 (C), 166.59 (C), 156.89 (C), 152.43 (C), 138.26 (C), 137.80 (C), 133.69 (C), 133.47 (C), 132.16 (C), 131.99 (C), 131.44 (CH), 129.86 (CH), 71.38 (CH_2), 71.36 (CH_2), 70.65 (CH_2), 67.91 (CH_2), 54.99 (CH), 40.69 (CH_2), 40.40 (CH_2), 38.51 (CH_2), 14.39 (CH_3), 12.94 (CH_3), 11.56 (CH_3); TFA peaks: 161.02 (q, $^2J_{CF}$ = 38.1 Hz, C), 117.34 (q, $^1J_{CF}$ = 288.4 Hz, C). HRMS (ESI) calculated for $C_{25}H_{32}ClN_6O_3S$ $[M+H]^+$ = 531.1940, found 531.1939.

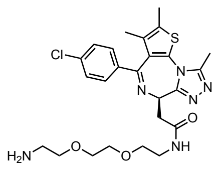

(*R*)-JQ1-PEG$_2$-amine (S23)**.** To a solution of (-)-JQ1-$CO_2{}^tBu$ (**S20**, 248.8 mg, 0.54 mmol, 1 equiv) in DCM (10 mL) was added TFA (1 mL). This light orange solution was stirred at ambient temperature for 24 h. The solvent was removed under reduced pressure and the crude was dissolved in DMF (3 mL). To this solution were successively added DIEA (976 µL, 5.44 mmol, 10 equiv), HATU (311 mg, 0.82 mmol, 1.5 equiv), and **S17** (203 mg, 0.82 mmol, 1.5 equiv). The reaction mixture was stirred at ambient temperature for 18 h. The solvent was removed under reduced pressure and the oil was resuspended in DCM (4 mL), and TFA (1 mL) was added. The reaction mixture was stirred at ambient temperature for 18 h. The solvent was removed under reduced pressure and the product was purified by preparative HPLC using a 5–95% CH_3CN/H_2O linear gradient with a constant 0.1% v/v TFA. Product-containing fractions were combined and lyophilized to obtain **S23** as a yellow gum (5×TFA salt, 350.7 mg, 58.5% over three steps). The molar equivalents of TFA were determined via ^{19}F NMR by using fluorobenzene as an internal standard. ^1H NMR (CD_3OD, 400 MHz) δ 7.49 – 7.44 (m, 2H), 7.43 – 7.42 (m, 2H), 4.71 (dd, J = 8.3, 6.0 Hz, 1H), 3.74 – 3.70 (m, 2H), 3.68 (s, 4H), 3.62 (t, J = 5.6 Hz, 2H), 3.53 – 3.34 (m, 4H), 3.13 (t, J = 5.0 Hz, 2H), 2.75 (s, 3H), 2.46 (s, 3H), 1.70 (s, 3H). ^{13}C NMR (CD_3OD, 101 MHz) δ 172.68 (C), 166.65 (C), 156.81 (C), 152.51 (C), 138.32 (C), 137.64 (C), 133.87 (C), 133.41 (C), 132.22 (C), 131.97 (C), 131.49 (CH), 129.86 (CH), 71.33 (CH_2), 70.62 (CH_2), 67.89 (CH_2), 54.90 (CH), 40.65 (CH_2), 40.39 (CH_2), 38.38 (CH_2), 14.40 (CH_3), 12.96 (CH_3), 11.55 (CH_3); TFA peaks: 161.41 (q, $^2J_{CF}$ = 38.3 Hz, C), 158.94 (q, $^2J_{CF}$ = 41.3 Hz, C), 117.34 (q, $^1J_{CF}$ = 289.0 Hz, C), 116.37 (q, $^1J_{CF}$ = 284.4 Hz, C). HRMS (ESI) calculated for $C_{25}H_{32}ClN_6O_3S$ $[M+H]^+$ = 531.1940, found 531.1945.

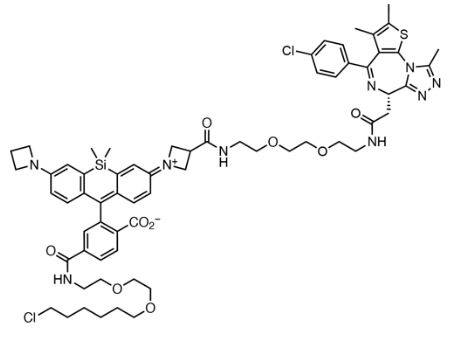

(*S*)-JQ1-JF$_{646}$-HaloTag ligand (18$_{HTL}$)**.** The TFA salt of **10** (23 mg, 27 µmol, 1 equiv) was dissolved in DMF (3 mL). To this solution were added DIEA (49 µL, 267 µmol, 10 equiv) and HATU (12.2 mg, 32 µmol, 1.2 equiv). The reaction mixture was stirred at ambient temperature for 5 min, after which **S19** (44.1 mg, 40 µmol, 1.5 equiv) was added. The reaction mixture was stirred

for a further 16 h at ambient temperature. The solvent was removed under reduced pressure, and the product was purified by preparative HPLC using a 40–60% CH_3CN/H_2O linear gradient with a constant 0.1% v/v TFA. Product-containing fractions were combined and lyophilized to obtain **18_{HTL}** as a blue solid (4×TFA salt, 29 mg, 63.2%). The molar equivalents of TFA were determined via ^{19}F NMR by using fluorobenzene as an internal standard. ^1H NMR (CD_3OD, 400 MHz; rotamers observed) δ 8.27 (dd, *J* = 8.2, 2.9 Hz, 1H), 8.11 (dt, *J* = 8.2, 2.0 Hz, 1H), 7.69 (dt, *J* = 7.3, 3.7 Hz, 1H), 7.41 (dd, *J* = 8.6, 2.9 Hz, 2H), 7.36 (d, *J* = 8.3 Hz, 2H), 6.93 – 6.76 (m, 4H), 6.34 (dd, *J* = 9.3, 2.6 Hz, 1H), 6.15 – 6.04 (m, 1H), 4.64 (dd, *J* = 8.8, 5.5 Hz, 1H), 4.36 – 4.18 (m, 8H), 3.66 – 3.55 (m, 16H), 3.53 – 3.48 (m, 3H), 3.46 – 3.40 (m, 7H), 3.36 – 3.33 (m, 1H), 2.61 (d, *J* = 8.9 Hz, 3H), 2.52 (p, *J* = 7.7 Hz, 2H), 2.43 (d, *J* = 2.9 Hz, 3H), 1.74 – 1.66 (m, 5H), 1.50 (p, *J* = 6.8 Hz, 2H), 1.38 (q, *J* = 7.6 Hz, 2H), 1.33 (dd, *J* = 9.0, 5.8 Hz, 2H), 0.56 (d, *J* = 8.3 Hz, 3H), 0.52 (d, *J* = 3.5 Hz, 3H). ^{13}C NMR (CD_3OD, 101 MHz; rotamers observed) δ 173.91 (C), 172.76 (C), 168.09 (C), 167.99 (C), 166.21 (C), 156.98 (C), 154.20 (C), 153.51 (C), 152.24 (C), 139.15 (C), 138.06 (C), 137.91 (C), 133.45 (C), 133.36 (C), 132.06 (C), 132.03 (C), 131.94 (C), 131.90 (C), 131.35 (CH), 129.81 (CH), 129.53 (CH), 128.97 (CH), 119.77 (CH), 119.15 (CH), 113.28 (CH), 112.74 (CH), 72.12 (CH_2), 71.40 (CH_2), 71.34 (CH_2), 71.21 (CH_2), 71.14 (CH_2), 70.61 (CH_2), 70.43 (CH_2), 70.38 (CH_2), 55.28 (CH_2), 55.09 (CH), 53.28 (CH_2), 45.74 (CH_2), 41.16 (CH_2), 40.66 (CH_2), 40.56 (CH_2), 38.71 (CH_2), 34.78 (CH), 33.71 (CH_2), 30.45 (CH_2), 27.68 (CH_2), 26.43 (CH_2), 16.97 (CH_2), 14.44 (CH_3), 12.98 (CH_3), 11.64 (CH_3), −0.81 (CH_3), −1.75 (CH_3); five aromatic quaternary carbons were not observed. Analytical HPLC: t_R= 13.8 min, 96.5% purity (10–95% CH_3CN/H_2O linear gradient over 20 min with constant 0.1% v/v TFA, 1 mL/min flow rate, detection at 650 nm). HRMS (ESI) calculated for $C_{65}H_{78}N_9O_9SCl_2Si$ $[M+H]^+$ = 1258.4784, found 1258.4795.

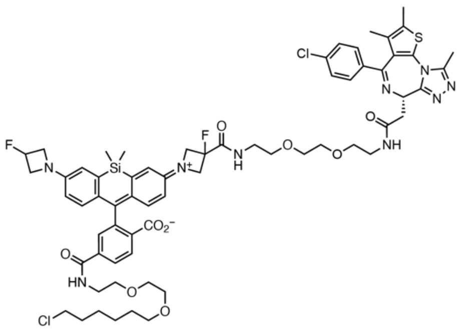

(*S*)-JQ1-JF$_{635}$-HaloTag ligand (19_{HTL}**).** The TFA salt of **11_{HTL}** (61.6 mg, 69 μmol, 1 equiv) was dissolved in DMF (3 mL). To this solution were added DIEA (121 μL, 109 μmol, 10 equiv), EDC·HCl (25.8 mg, 135 μmol, 2 equiv), HATU (51.3 mg, 135 μmol, 2 equiv). The reaction mixture was stirred at ambient temperature for 10 min, after which **S19** (55.1 mg, 50 μmol, 0.72 equiv) was added. The reaction mixture was stirred for a further 16 h at ambient temperature. The solvent was removed under reduced pressure, and the product was purified by preparative HPLC using a 40–65% CH_3CN/H_2O linear gradient with a constant 0.1% v/v TFA. Product-containing

fractions were combined and lyophilized to obtain **19**$_{\mathbf{HTL}}$ as a blue solid (4×TFA salt, 28.8 mg, 32.9%). The molar equivalents of TFA were determined via ^{19}F NMR by using fluorobenzene as an internal standard. ^{1}H NMR (CD_3OD, 400 MHz, 320 K) δ 8.07 (qd, J = 8.3, 3.6 Hz, 2H), 7.67 (d, J = 6.4 Hz, 1H), 7.43 (d, J = 8.3 Hz, 2H), 7.39 – 7.33 (m, 2H), 6.90 – 6.75 (m, 4H), 6.38 (dd, J = 8.9, 2.6 Hz, 1H), 6.30 (td, J = 8.7, 2.6 Hz, 1H), 5.53 – 5.46 (m, 0.5H), 5.39 – 5.32 (m, 0.5H), 4.70 – 4.58 (m, 1H; buried under the water peak), 4.48 – 4.27 (m, 4H), 4.26 – 4.14 (m, 2H), 4.14 – 4.00 (m, 2H), 3.65 – 3.56 (m, 12H), 3.55 – 3.50 (m, 4H), 3.50 – 3.33 (m, 10H), 2.66 (d, J = 8.3 Hz, 3H), 2.40 (d, J = 3.5 Hz, 3H), 1.73 – 1.63 (m, 5H), 1.47 (p, J = 6.8 Hz, 2H), 1.37 (p, J = 7.0 Hz, 2H), 1.29 (q, J = 8.3 Hz, 2H), 0.61 (d, J = 5.0 Hz, 3H), 0.53 (d, J = 4.9 Hz, 3H). ^{13}C NMR (CD_3OD, 101 MHz; rotamers observed) δ 172.69 (C), 170.72 (C), 170.20 (d, $^2J_{CF}$ = 21.3 Hz, C), 168.40 (C), 166.32 (C), 156.89 (C), 152.46 (C), 152.28 (C), 151.60 (C), 141.04 (C), 138.11 (C), 137.73 (C), 133.54 (C), 133.39 (C), 132.99 (C), 132.12 (C), 131.90 (C), 131.44 (CH), 129.80 (CH), 129.31 (CH), 128.04 (CH), 125.80 (CH), 118.87 (CH), 118.15 (CH), 114.27 (CH), 114.07 (CH), 92.03 (d, $^1J_{CF}$ = 221.9 Hz, C), 84.12 (d, $^1J_{CF}$ = 202.6 Hz, C), 72.11 (CH_2), 71.36 (CH_2), 71.26 (CH_2), 71.19 (CH_2), 71.11 (CH_2), 70.66 (CH_2), 70.33 (CH_2), 70.20 (CH_2), 62.30 (d, $^2J_{CF}$ = 25.1 Hz, CH_2), 60.62 (d, $^2J_{CF}$ = 24.5 Hz, CH_2), 54.99 (CH), 45.74 (CH_2), 41.14 (CH_2), 40.52 (CH_2), 40.28 (CH_2), 38.52 (CH_2), 33.68 (CH_2), 30.41 (CH_2), 27.66 (CH_2), 26.39 (CH_2), 14.46 (CH_3), 12.99 (CH_3), 11.64 (CH_3), −0.10 (CH_3), −1.30 (CH_3). TFA peaks: 161.33 (q, $^2J_{CF}$ = 37.5 Hz, C), 158.94 (q, $^2J_{CF}$ = 41.4 Hz, C), 116.04 (q, $^1J_{CF}$ = 285.1 Hz, C); five aromatic quaternary carbons were not observed. ^{19}F NMR (CD_3OD, 376 MHz) δ −75.14, -75.62, −161.97, −179.86. Analytical HPLC: t_R= 14.7 min, 98.4% purity (30–95% CH_3CN/H_2O linear gradient over 20 min with constant 0.1% v/v TFA, 1 mL/min flow rate, detection at 650 nm). HRMS (ESI) calculated for $C_{65}H_{76}N_9O_9F_2SCl_2Si$ $[M+H]^+$ = 1294.4596, found 1294.4609.

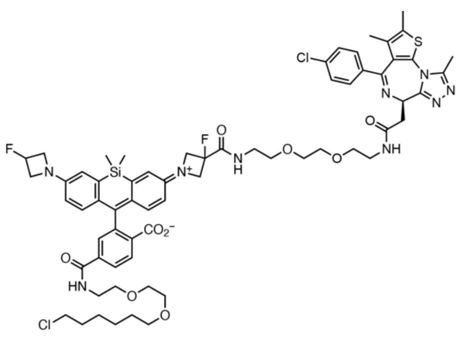

(*R*)-JQ1-JF$_{635}$-HaloTag ligand (20$_{\mathrm{HTL}}$**).** The TFA salt of **11**$_{\mathbf{HTL}}$ (8.5 mg, 11 μmol, 1 equiv) was dissolved in DMF (2 mL). To this solution were added DIEA (20 μL, 109 μmol, 10 equiv), **S23** (30 mg, 26 μmol, 1.7 equiv), EDC·HCl (6.1 mg, 32 μmol, 3 equiv), and HATU (12.2 mg, 32 μmol, 3 equiv). The reaction mixture was stirred at ambient temperature for 16 h. The solvent was removed under reduced pressure, and the product was purified by preparative HPLC using a 5–95% CH_3CN/H_2O linear gradient with a constant 0.1% v/v TFA. Product-containing fractions were combined and lyophilized to obtain **20**$_{\mathbf{HTL}}$ as a blue solid (TFA salt, 8.8 mg, 52.5%). ^{1}H NMR

(CD_3OD, 400 MHz) δ 8.36 (br s, 1 H), 8.07 – 8.03 (m, 2H), 7.69 – 7.66 (m, 1H), 7.42 – 7.35 (m, 4H), 6.84 – 6.79 (m, 3H), 6.74 (d, *J* = 8.8 Hz, 1H), 6.40 (ddd, *J* = 8.9, 2.7, 1.6 Hz, 1H), 6.29 – 6.23 (m, 1H), 5.52 – 5.48 (m, 0.5H), 5.39 – 5.34 (m, 0.5H), 4.66 – 4.61 (m, 1H), 4.45 – 3.93 (m, 8H), 3.67 – 3.41 (m, 22H), 3.39 (t, *J* = 6.5 Hz, 2H), 3.29 – 3.27 (m, 2H), 2.65 (2 × s, 3H), 2.41 (2 × s, 3H), 1.73 – 1.60 (m, 5H), 1.46 (p, *J* = 6.8 Hz, 2H), 1.40 – 1.33 (m, 2H), 1.31 – 1.27 (m, 2H), 0.62 (2 × s, 3H), 0.53 (2 × s, 3H). Analytical HPLC: t_R= 15.7 min, 97.7% purity (5–95% CH_3CN/H_2O linear gradient over 20 min with constant 0.1% v/v TFA, 1 mL/min flow rate, detection at 254 nm). HRMS (ESI) calculated for $C_{65}H_{76}N_9O_9F_2SCl_2Si$ $[M+H]^+$ = 1294.4596, found 1294.4597.

NMR Spectra and HPLC Traces

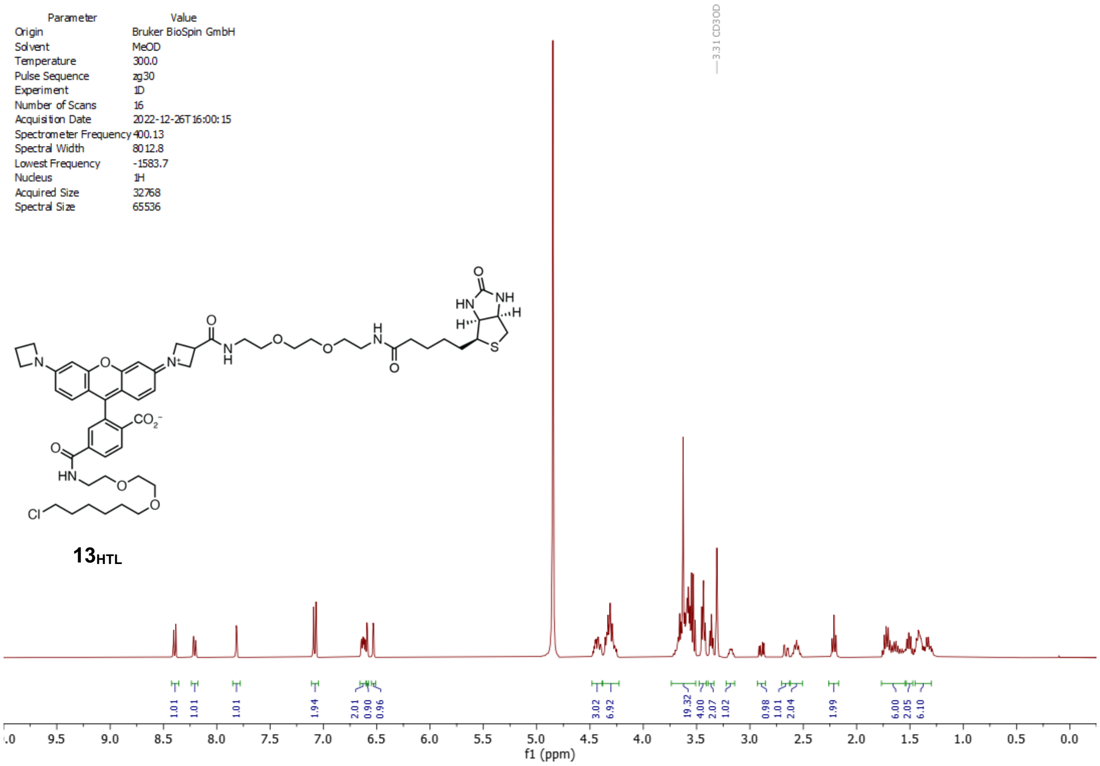

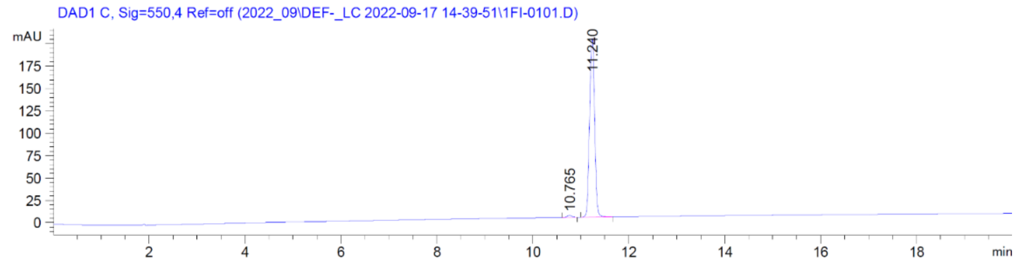

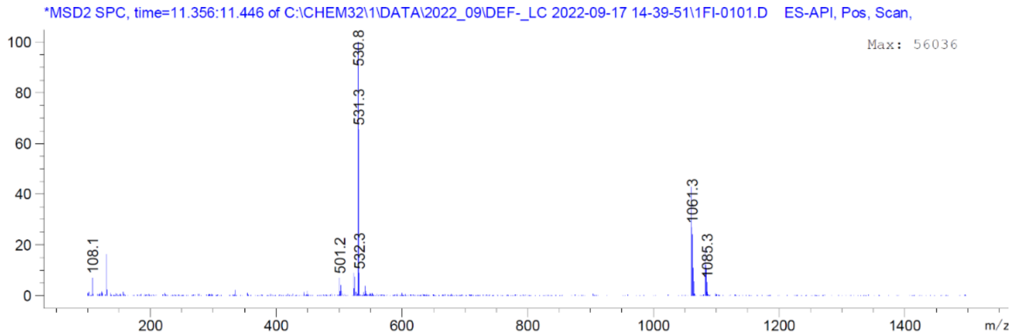

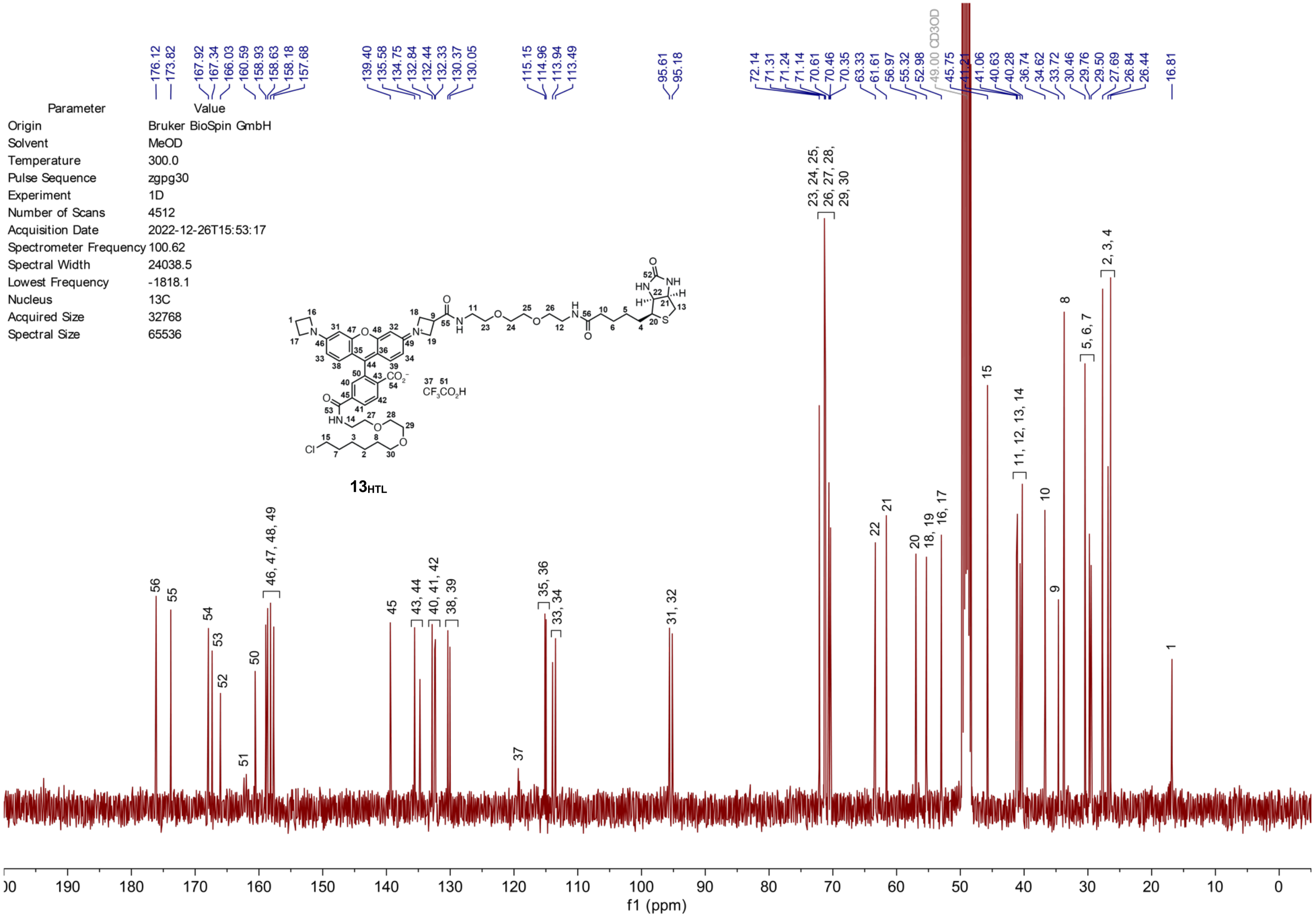
Parameter Value
Origin Bruker BioSpin GmbH
Solvent MeOD
Temperature 300.0
Pulse Sequence zgpg30
Experiment 1D
Number of Scans 4512
Acquisition Date 2022-12-26T15:53:17
Spectrometer Frequency 100.62
Spectral Width 24038.5
Lowest Frequency -1818.1
Nucleus 13C
Acquired Size 32768
Spectral Size 65536
13HTL
176.12
173.82
167.92
167.34
166.03
160.59
158.93
158.63
158.18
157.68
139.40
135.58
134.75
132.84
132.44
132.33
130.37
130.05
115.15
114.96
113.94
113.49
95.61
95.18
72.14
71.31
71.24
71.14
70.61
70.46
70.35
63.33
61.61
56.97
55.32
52.98
49.00 CD3OD
45.75
41.21
41.06
40.63
40.28
36.74
34.62
33.72
30.46
29.76
29.50
27.69
26.84
26.44
16.81
f1 (ppm)

Parameter	Value
Origin	Bruker BioSpin GmbH
Solvent	MeOD
Temperature	300.0
Pulse Sequence	zg30
Experiment	1D
Number of Scans	32
Acquisition Date	2022-12-25T09:33:54
Spectrometer Frequency	400.13
Spectral Width	8012.8
Lowest Frequency	-1583.5
Nucleus	1H
Acquired Size	32768
Spectral Size	65536

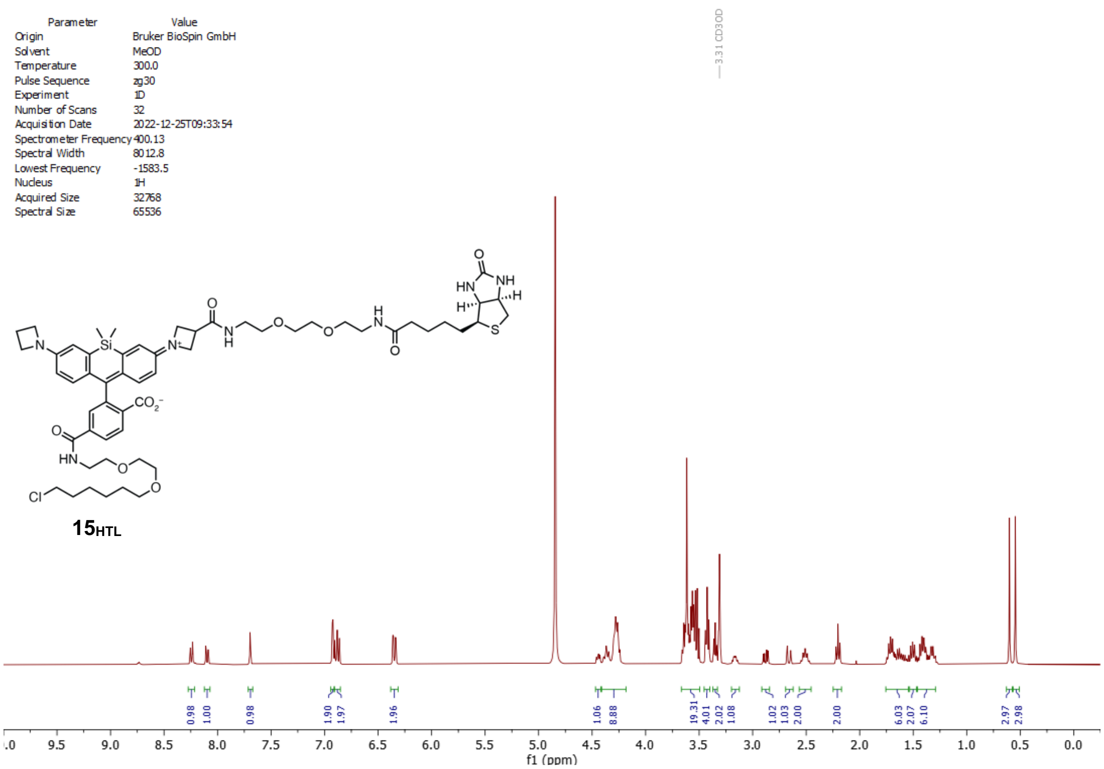

15HTL

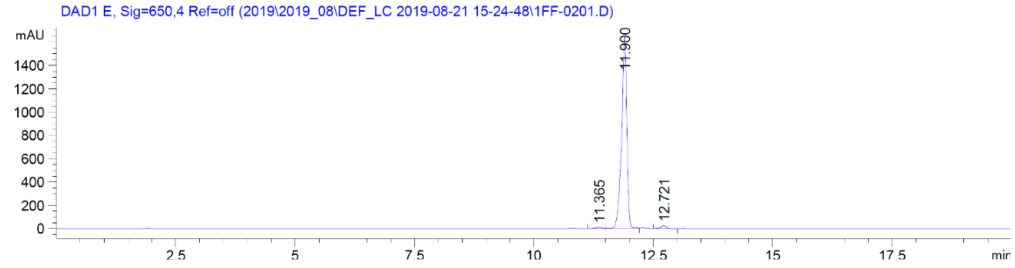

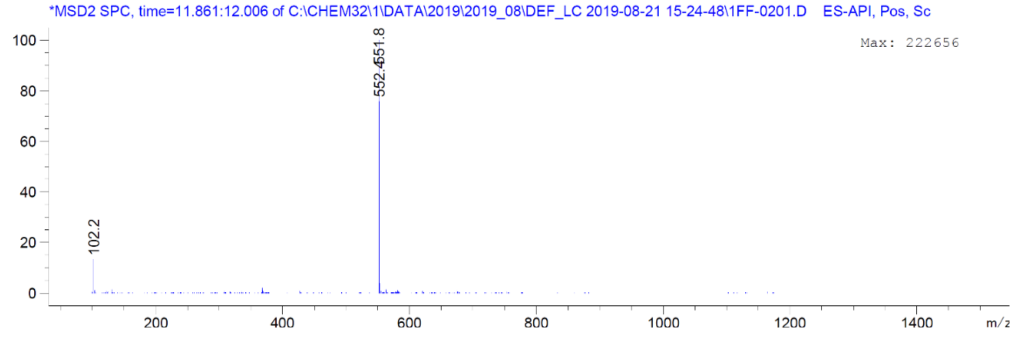

Parameter	Value
Origin	Bruker BioSpin GmbH
Solvent	MeOD
Temperature	300.0
Pulse Sequence	zgpg30
Experiment	1D
Number of Scans	19504
Acquisition Date	2022-12-26T10:50:14
Spectrometer Frequency	100.62
Spectral Width	24038.5
Lowest Frequency	-1818.4
Nucleus	13C
Acquired Size	32768
Spectral Size	65536

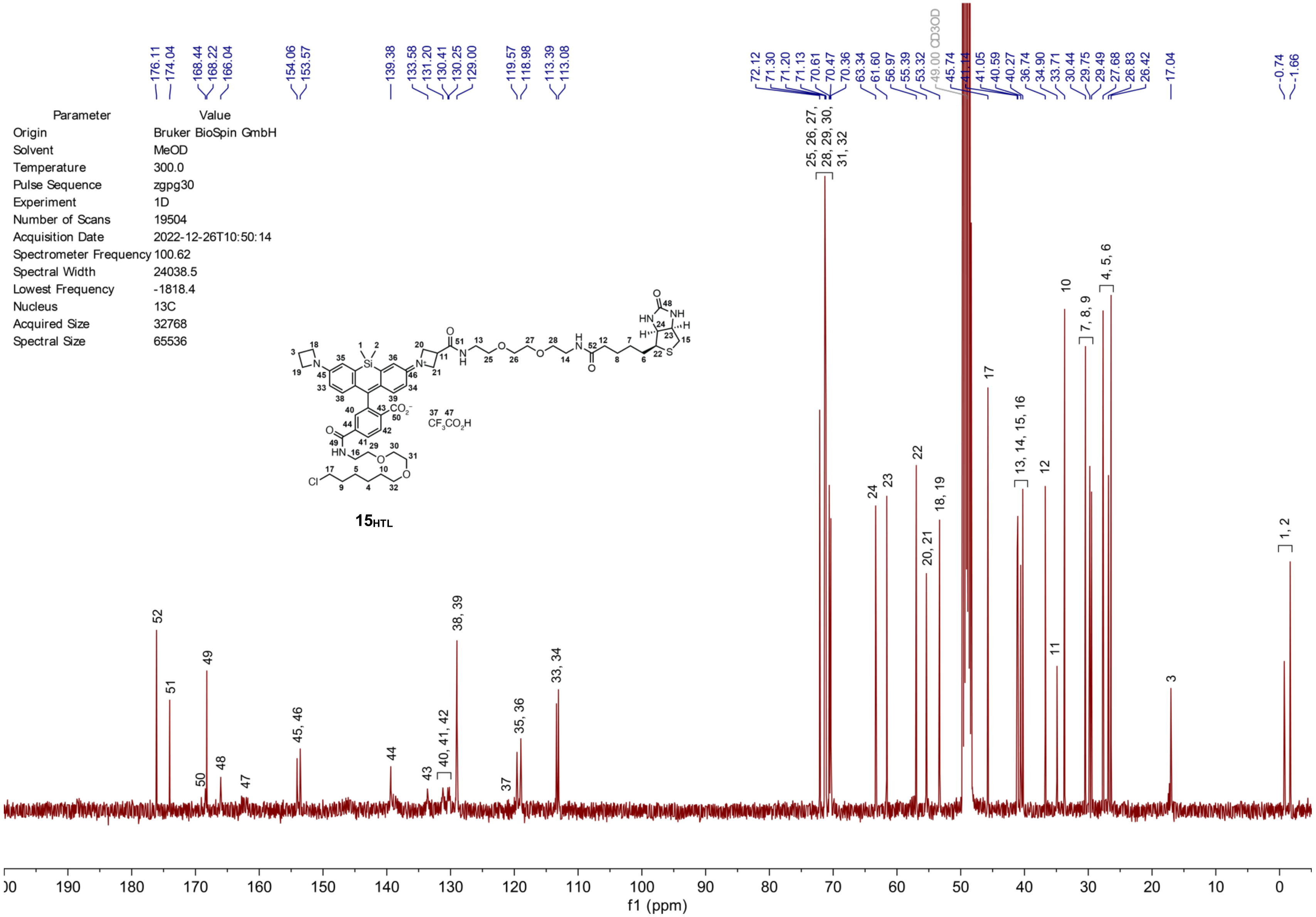

15_{HTL}

Parameter	Value
Solvent	CDCl3
Temperature	295.8
Pulse Sequence	zg30
Experiment	1D
Number of Scans	16
Acquisition Date	2023-06-29T09:52:15
Spectrometer Frequency	400.13
Spectral Width	8012.8
Lowest Frequency	-1545.6
Nucleus	1H
Acquired Size	32768
Spectral Size	65536

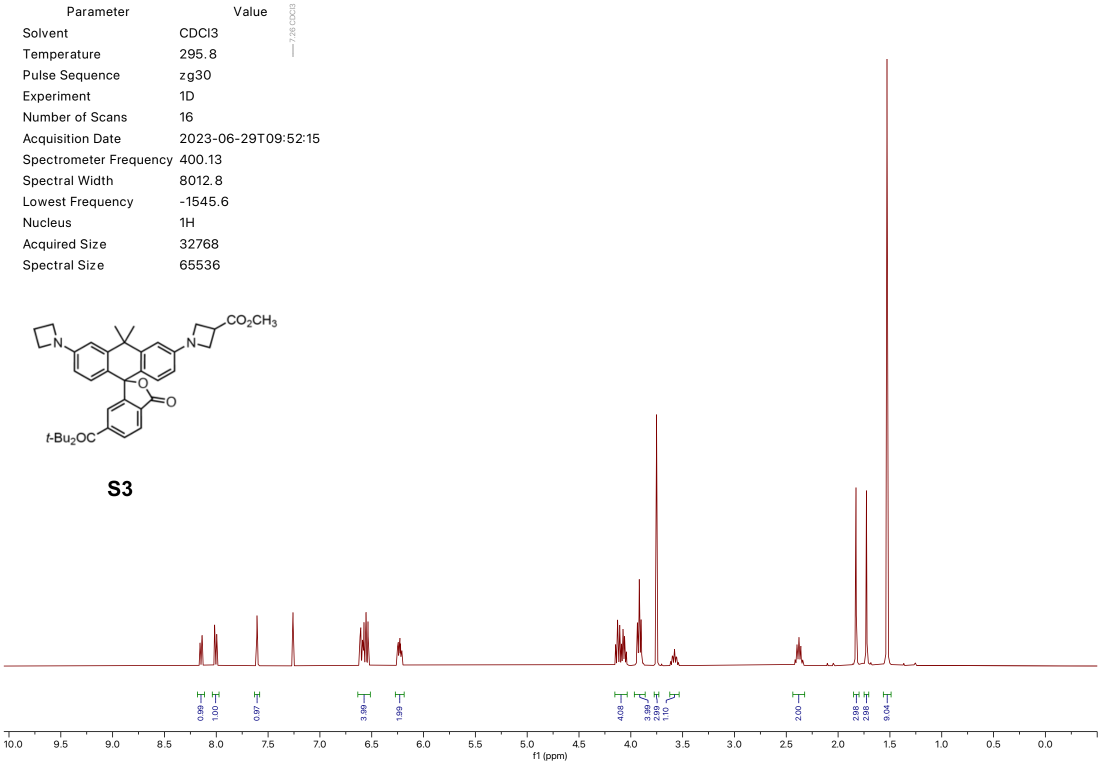

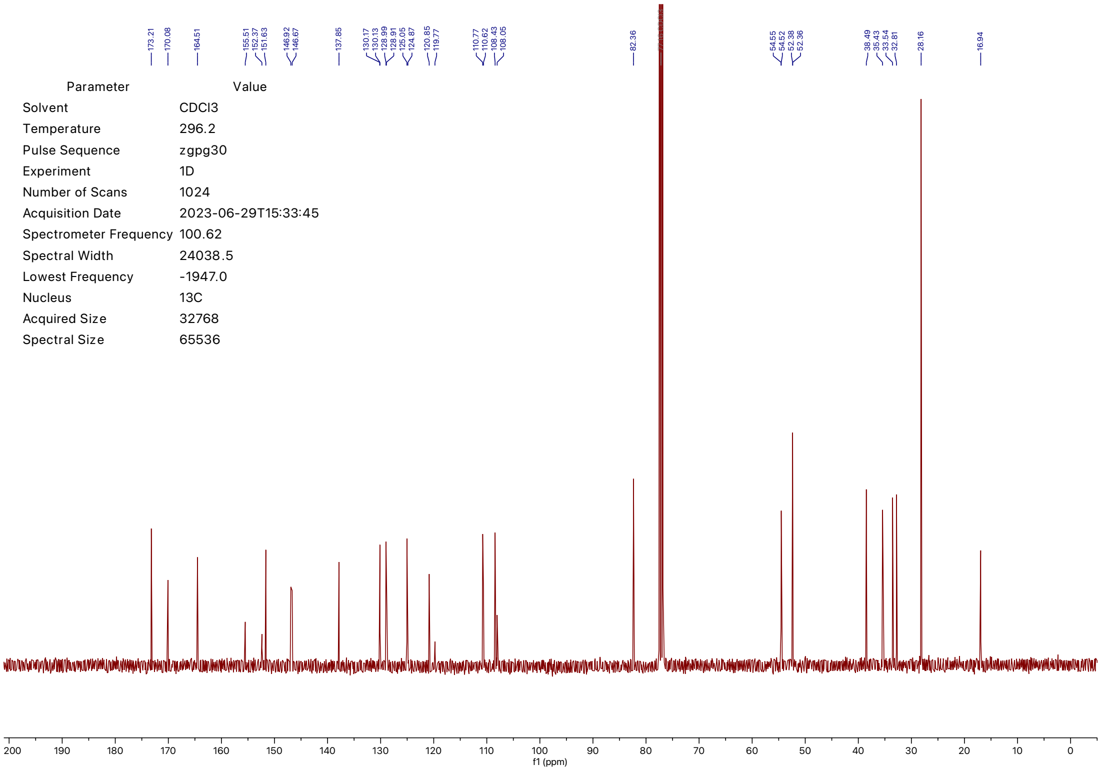

Parameter	Value
Solvent	CDCl3
Temperature	296.2
Pulse Sequence	zgpg30
Experiment	1D
Number of Scans	1024
Acquisition Date	2023-06-29T15:33:45
Spectrometer Frequency	100.62
Spectral Width	24038.5
Lowest Frequency	-1947.0
Nucleus	13C
Acquired Size	32768
Spectral Size	65536

Parameter	Value
Solvent	MeOD
Temperature	295.7
Pulse Sequence	zg30
Experiment	1D
Number of Scans	16
Acquisition Date	2023-07-17T08:33:56
Spectrometer Frequency	400.13
Spectral Width	8012.8
Lowest Frequency	-1543.3
Nucleus	1H
Acquired Size	32768
Spectral Size	65536

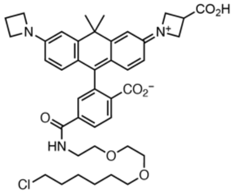

9

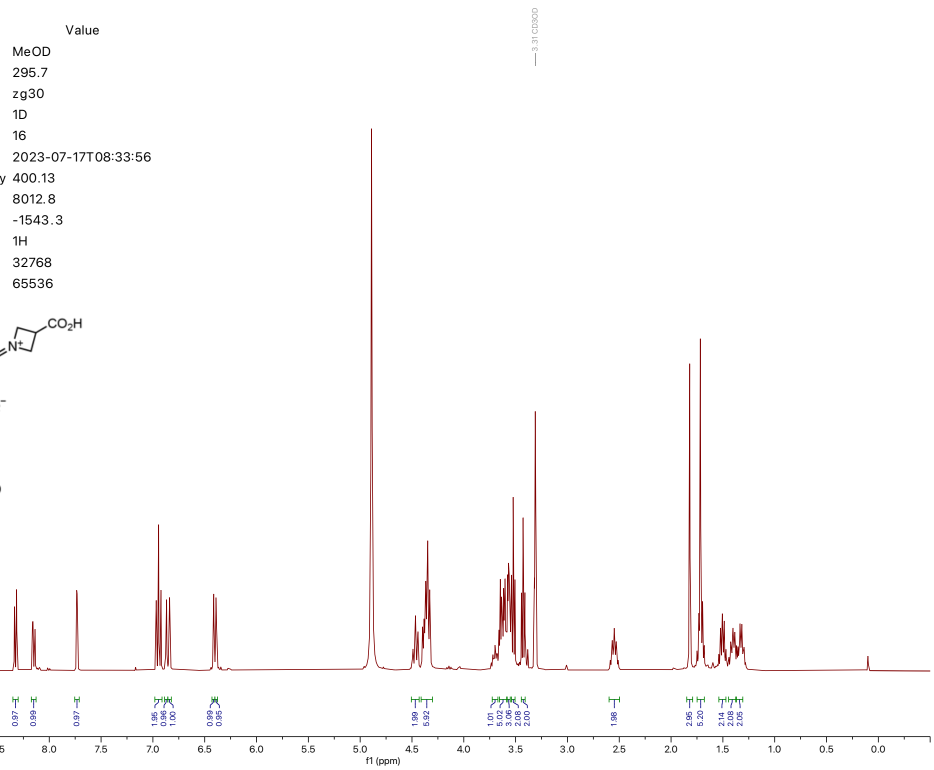

Parameter	Value
Solvent	MeOD
Temperature	296.0
Pulse Sequence	zgpg30
Experiment	1D
Number of Scans	9000
Acquisition Date	2023-07-17T04:35:34
Spectrometer Frequency	100.62
Spectral Width	24038.5
Lowest Frequency	-1818.0
Nucleus	13C
Acquired Size	32768
Spectral Size	65536

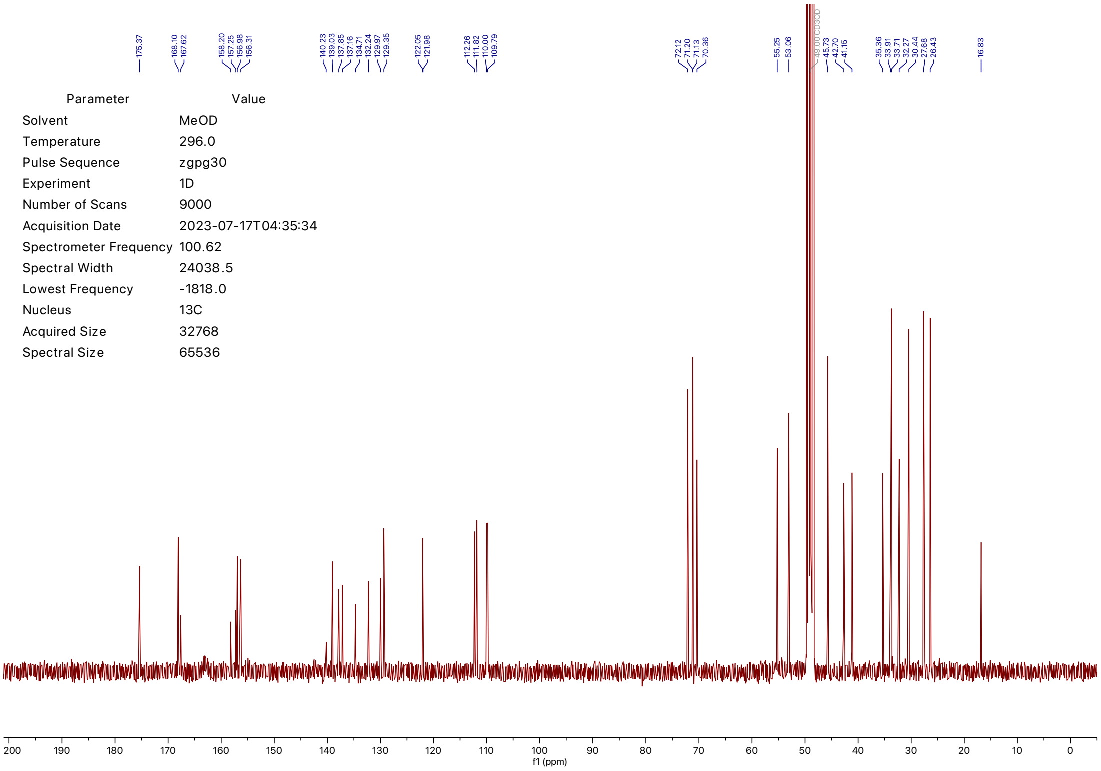

Parameter	Value
Solvent	MeOD
Temperature	295.8
Pulse Sequence	zg30
Experiment	1D
Number of Scans	16
Acquisition Date	2023-07-26T10:33:16
Spectrometer Frequency	400.13
Spectral Width	8012.8
Lowest Frequency	-1543.3
Nucleus	1H
Acquired Size	32768
Spectral Size	65536

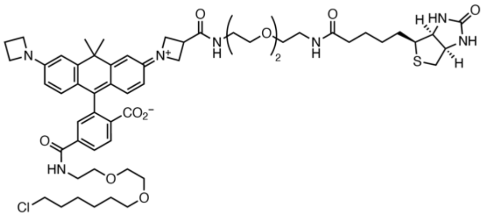

14$_{\mathbf{HTL}}$

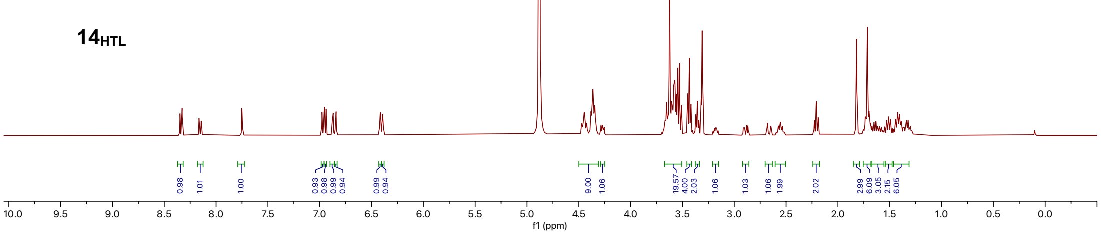

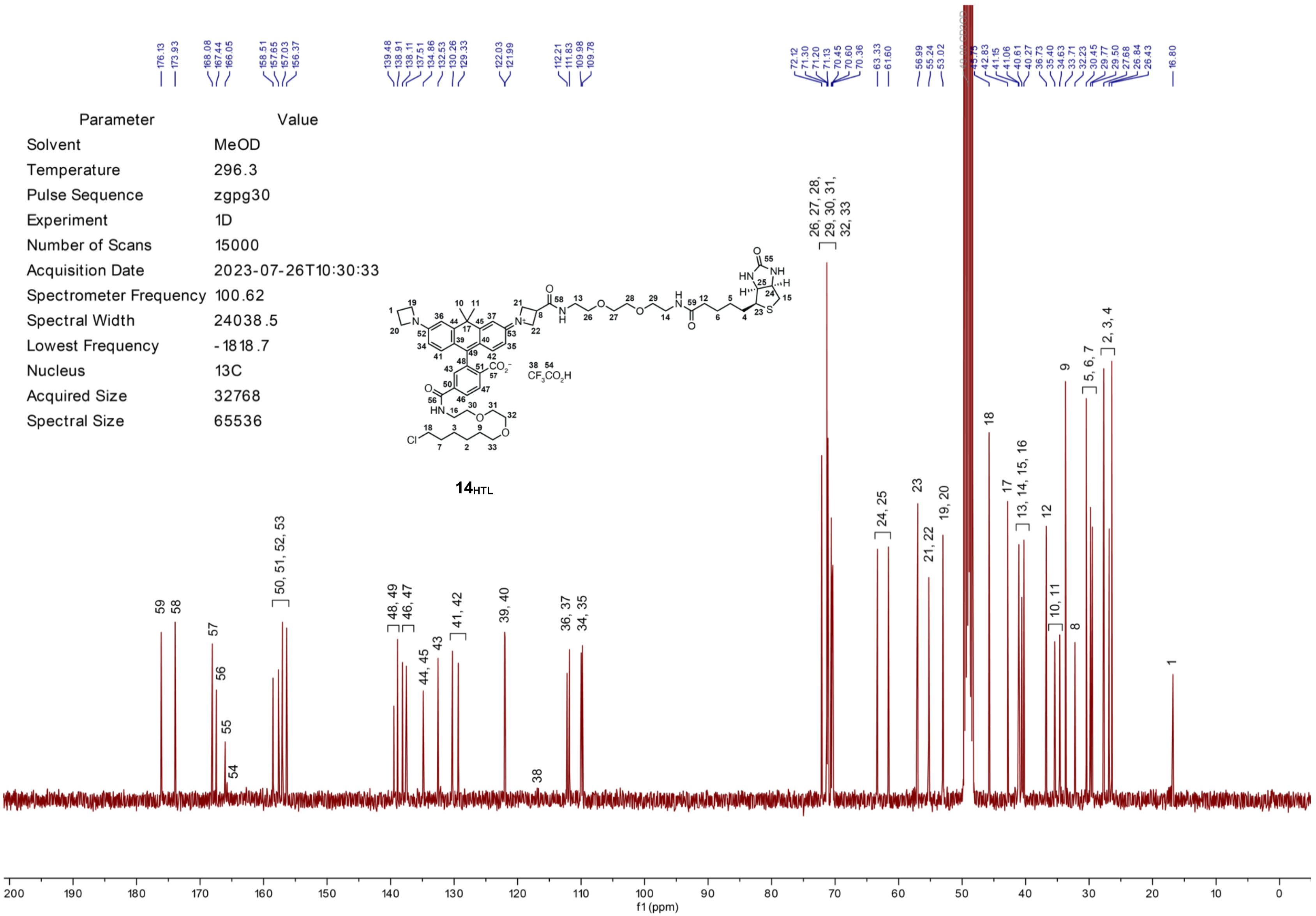

Parameter Value
Solvent MeOD
Temperature 296.3
Pulse Sequence zgpg30
Experiment 1D
Number of Scans 15000
Acquisition Date 2023-07-26T10:30:33
Spectrometer Frequency 100.62
Spectral Width 24038.5
Lowest Frequency -1818.7
Nucleus 13C
Acquired Size 32768
Spectral Size 65536
176.13
173.93
168.08
167.44
166.05
158.51
157.65
157.03
156.37
139.48
138.91
138.11
137.51
134.86
132.53
130.26
129.33
122.03
121.99
112.21
111.83
109.98
109.78
72.12
71.30
71.20
71.13
70.45
70.60
70.36
63.33
61.60
56.99
55.24
53.02
45.75
42.83
41.15
41.06
40.61
40.27
36.73
35.40
34.63
33.71
32.23
30.45
29.77
29.50
27.68
26.84
26.43
16.80
f1 (ppm)

14HTL

Parameter	Value
Origin	Bruker BioSpin GmbH
Solvent	CDCl3
Temperature	300.0
Pulse Sequence	zg30
Experiment	1D
Number of Scans	23
Acquisition Date	2019-09-06T08:55:00
Spectrometer Frequency	400.13
Spectral Width	8012.8
Lowest Frequency	-1545.4
Nucleus	1H
Acquired Size	32768
Spectral Size	65536

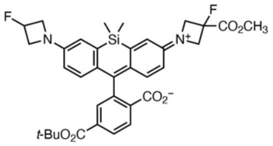

S13

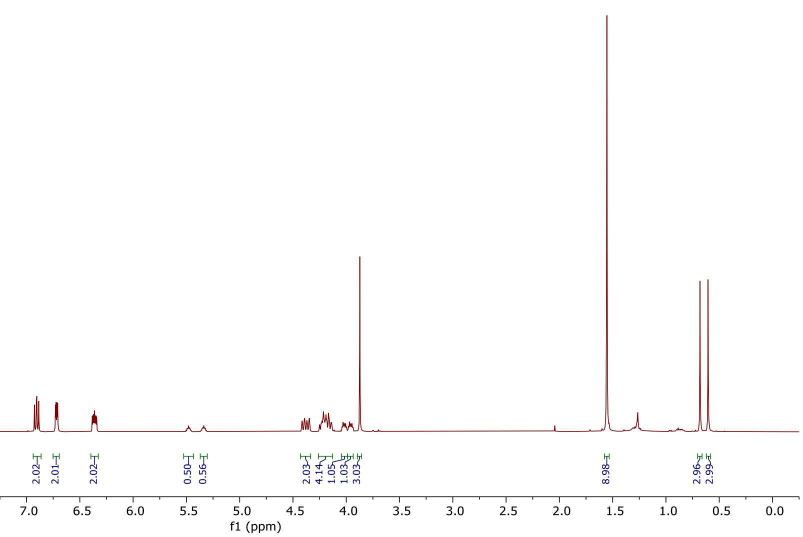

Parameter	Value
Origin	Bruker BioSpin GmbH
Solvent	CDCl3
Temperature	300.0
Pulse Sequence	zgpg30
Experiment	1D
Number of Scans	496
Acquisition Date	2019-09-06T08:59:00
Spectrometer Frequency	100.62
Spectral Width	24038.5
Lowest Frequency	-1949.6
Nucleus	13C
Acquired Size	32768
Spectral Size	65536

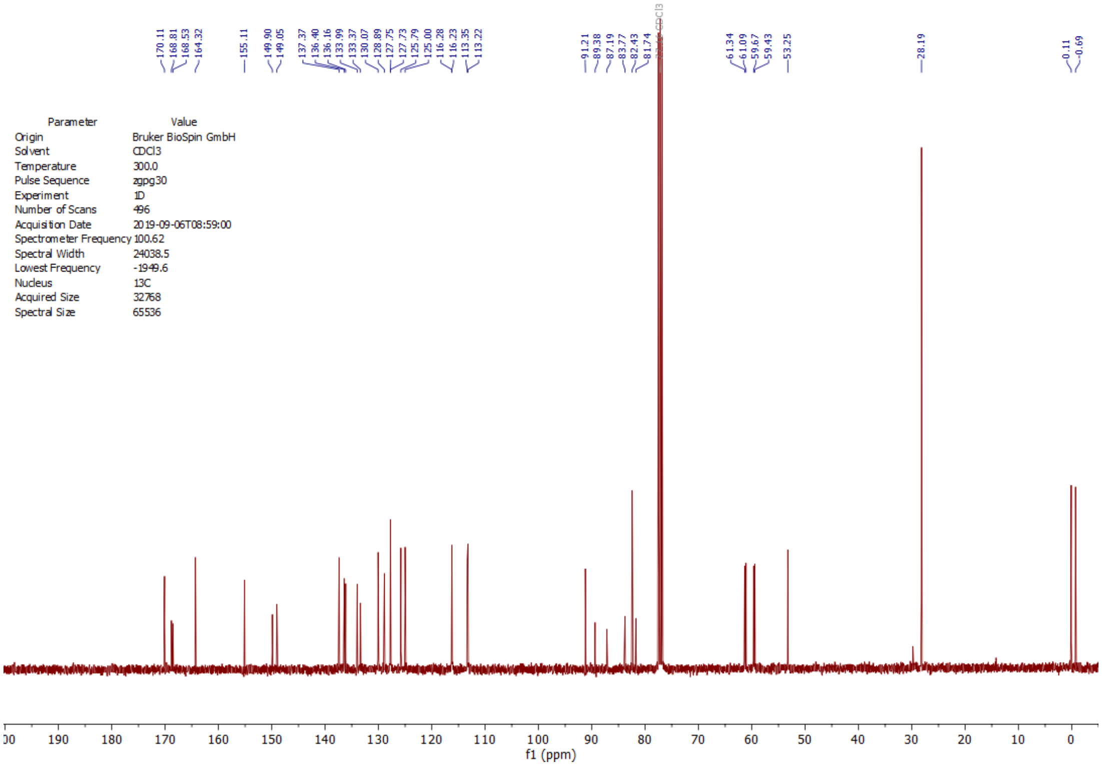

Parameter	Value
Origin	Bruker BioSpin GmbH
Solvent	CDCl3
Temperature	300.0
Pulse Sequence	zg30
Experiment	1D
Number of Scans	71
Acquisition Date	2019-09-08T19:01:00
Spectrometer Frequency	400.13
Spectral Width	8012.8
Lowest Frequency	-1545.4
Nucleus	1H
Acquired Size	32768
Spectral Size	65536

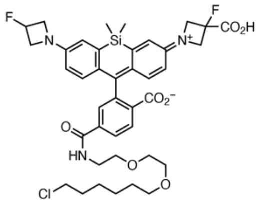

11

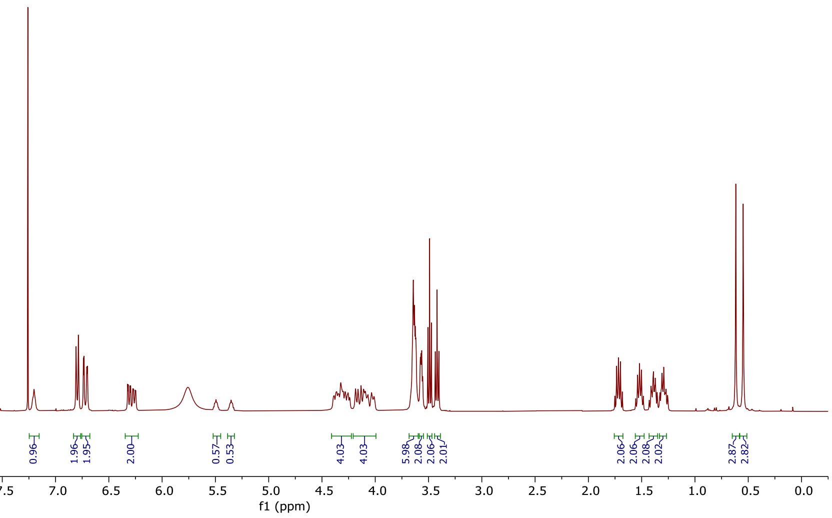

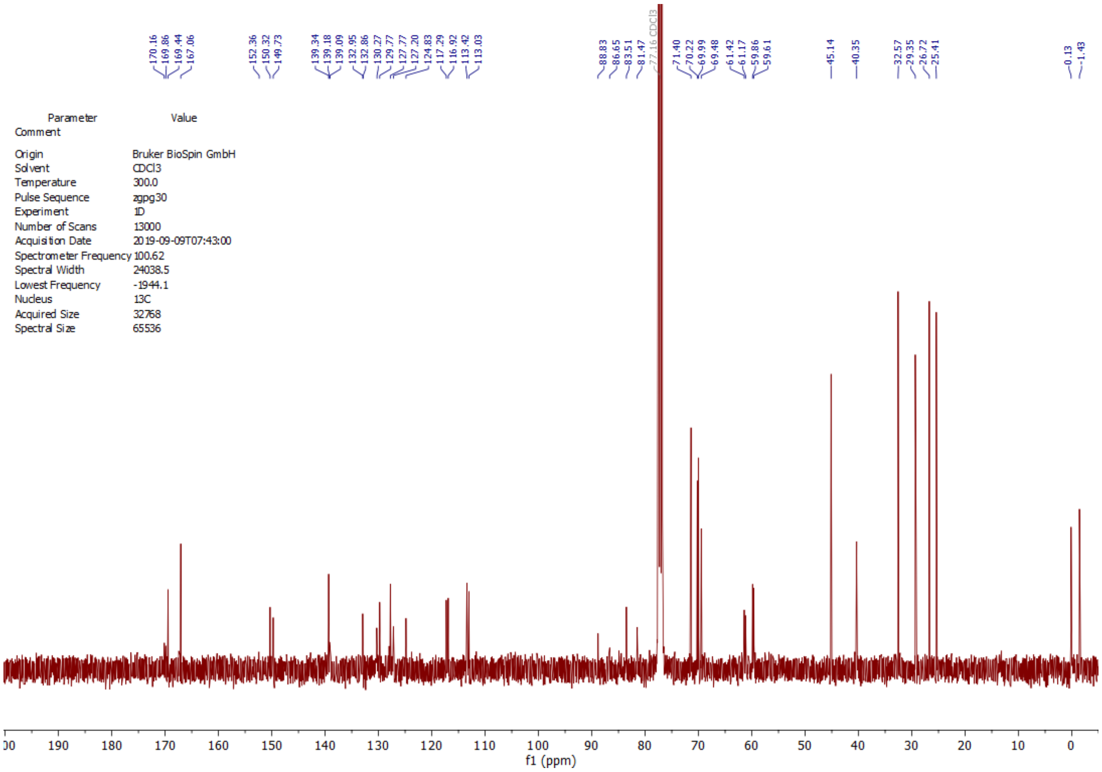

Parameter	Value
Comment	
Origin	Bruker BioSpin GmbH
Solvent	CDCl3
Temperature	300.0
Pulse Sequence	zgpg30
Experiment	1D
Number of Scans	13000
Acquisition Date	2019-09-09T07:43:00
Spectrometer Frequency	100.62
Spectral Width	24038.5
Lowest Frequency	-1944.1
Nucleus	13C
Acquired Size	32768
Spectral Size	65536

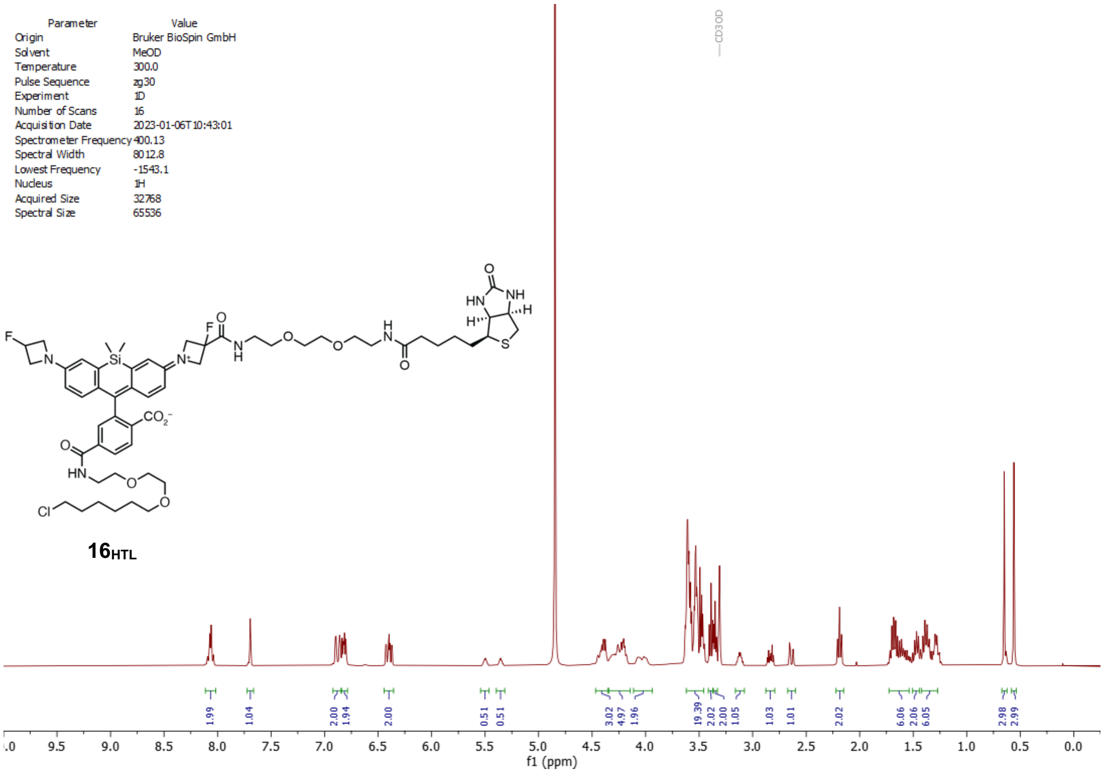

Parameter Value
Origin Bruker BioSpin GmbH
Solvent MeOD
Temperature 300.0
Pulse Sequence zg30
Experiment 1D
Number of Scans 16
Acquisition Date 2023-01-06T10:43:01
Spectrometer Frequency 400.13
Spectral Width 8012.8
Lowest Frequency -1543.1
Nucleus 1H
Acquired Size 32768
Spectral Size 65536
CD3OD
16HTL
f1 (ppm)

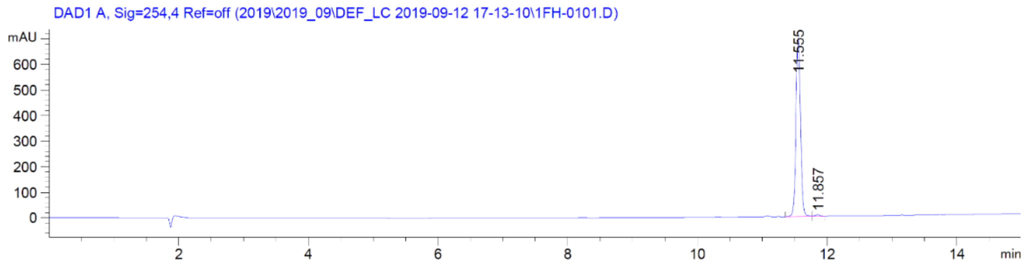

DAD1 A, Sig=254,4 Ref=off (2019\2019_09\DEF_LC 2019-09-12 17-13-10\1FH-0101.D)
mAU
11.555
11.857
min

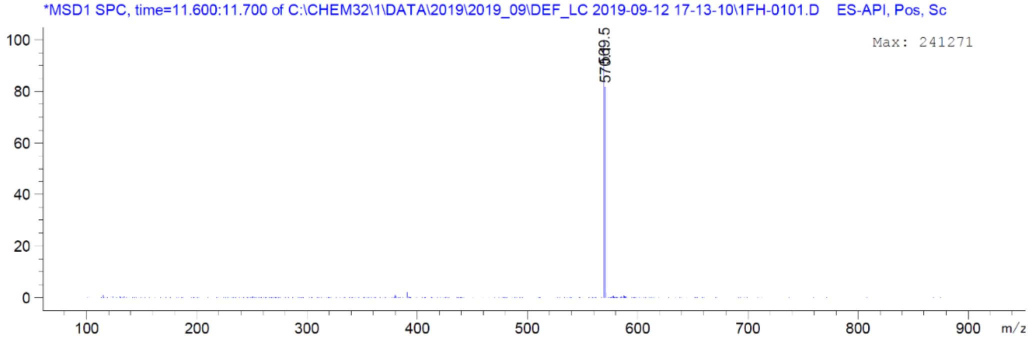

*MSD1 SPC, time=11.600:11.700 of C:\CHEM32\1\DATA\2019\2019_09\DEF_LC 2019-09-12 17-13-10\1FH-0101.D ES-API, Pos, Sc
Max: 241271
m/z

Parameter	Value
Origin	Bruker BioSpin GmbH
Solvent	MeOD
Temperature	320.0
Pulse Sequence	zgpg30
Experiment	1D
Number of Scans	20000
Acquisition Date	2023-03-25T12:04:57
Spectrometer Frequency	100.62
Spectral Width	24038.5
Lowest Frequency	-1958.0
Nucleus	13C
Acquired Size	32768
Spectral Size	65536

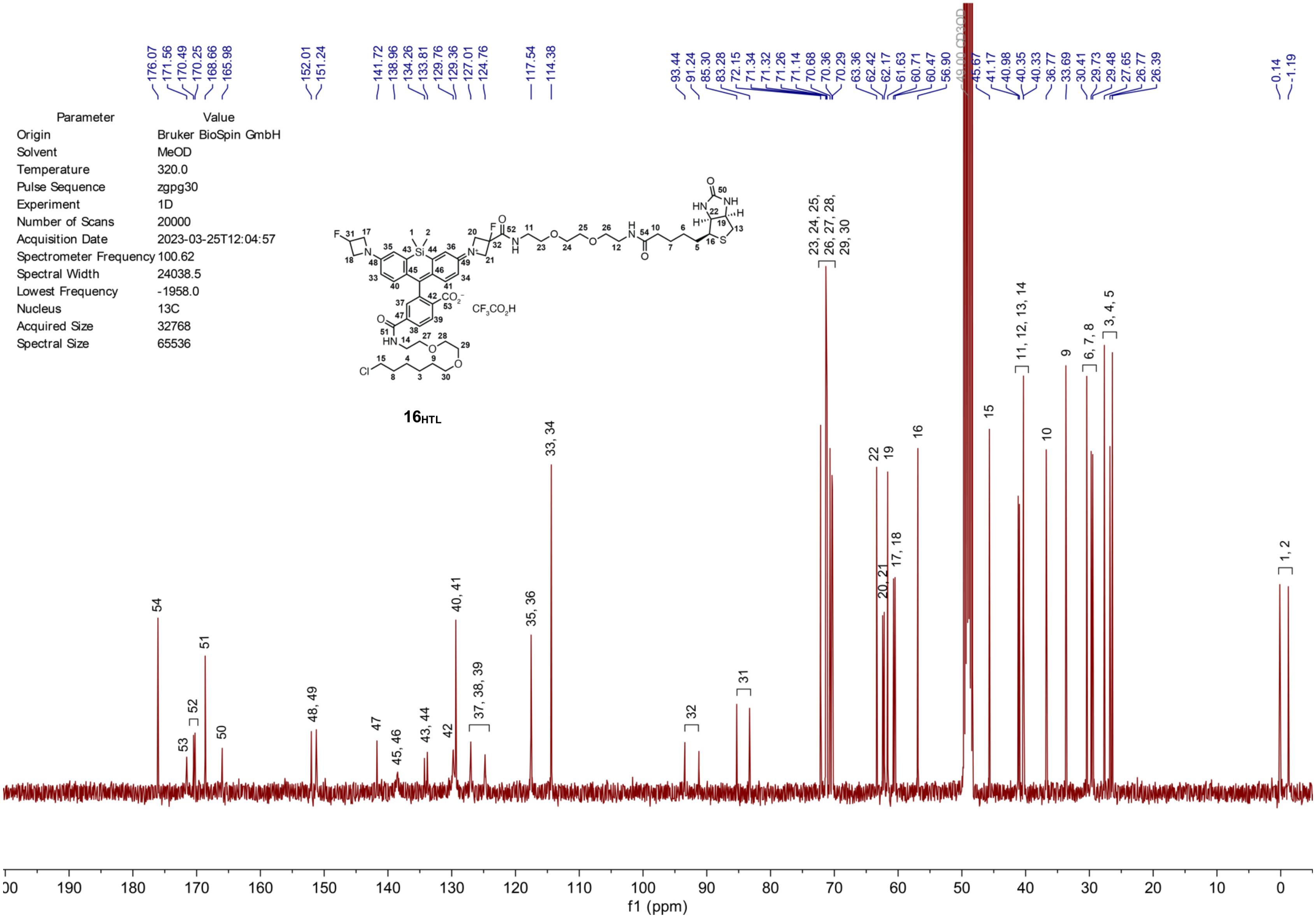

Parameter	Value
Origin	Bruker BioSpin GmbH
Solvent	MeOD
Temperature	295.3
Pulse Sequence	zg30
Experiment	1D
Number of Scans	128
Acquisition Date	2021-07-05T12:18:00
Spectrometer Frequency	400.13
Spectral Width	8012.8
Lowest Frequency	-1543.2
Nucleus	1H
Acquired Size	32768
Spectral Size	65536

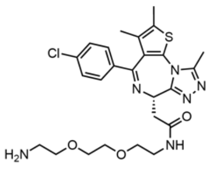

S19

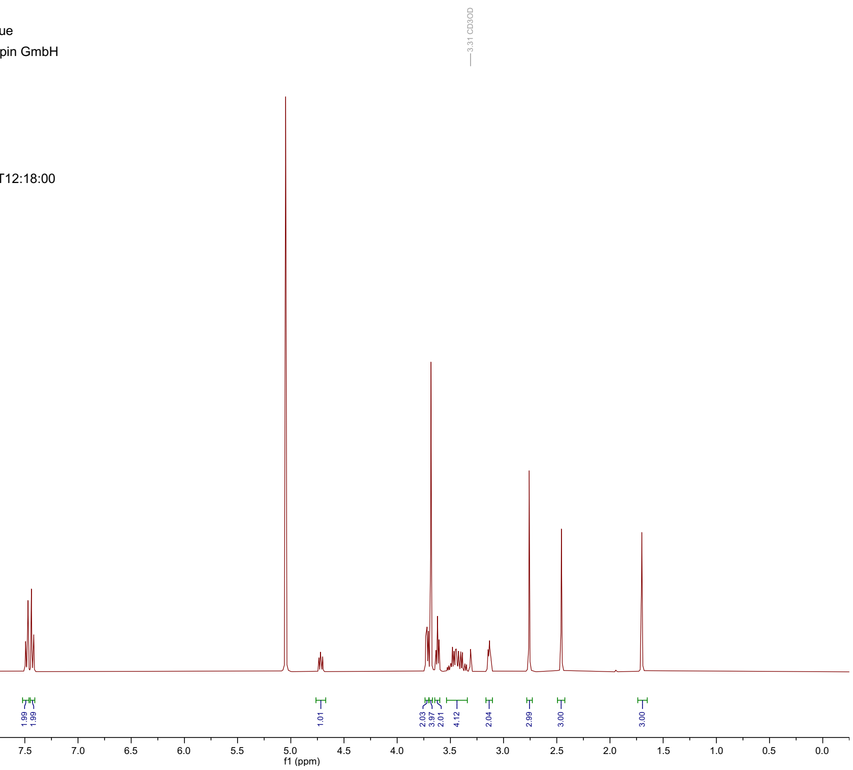

Parameter	Value
Origin	Bruker BioSpin GmbH
Solvent	CDCl3
Temperature	296.1
Pulse Sequence	zgpg30
Experiment	1D
Number of Scans	1024
Acquisition Date	2023-06-20T12:28:32
Spectrometer Frequency	100.62
Spectral Width	24038.5
Lowest Frequency	-2213.3
Nucleus	13C
Acquired Size	32768
Spectral Size	65536

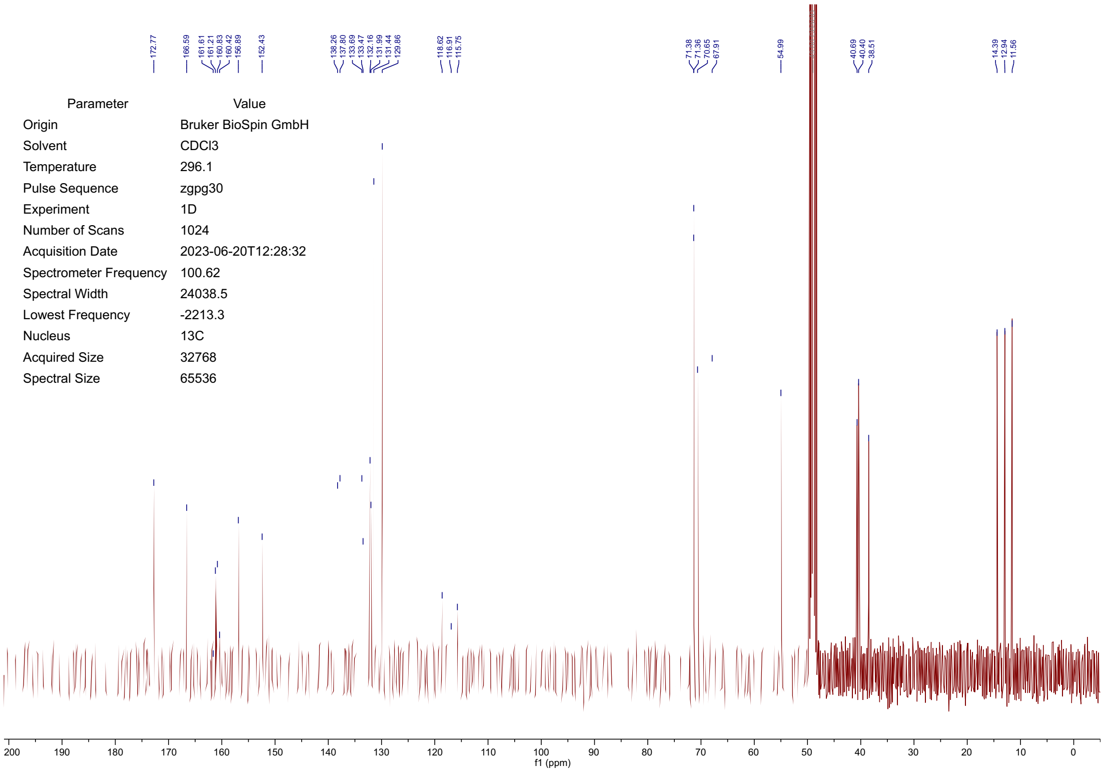

Parameter	Value
Origin	Bruker BioSpin GmbH
Solvent	MeOD
Temperature	295.4
Pulse Sequence	zg30
Experiment	1D
Number of Scans	128
Acquisition Date	2021-10-15T18:05:00
Spectrometer Frequency	400.13
Spectral Width	8012.8
Lowest Frequency	-1543.0
Nucleus	1H
Acquired Size	32768
Spectral Size	65536

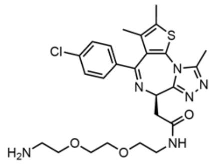

S23

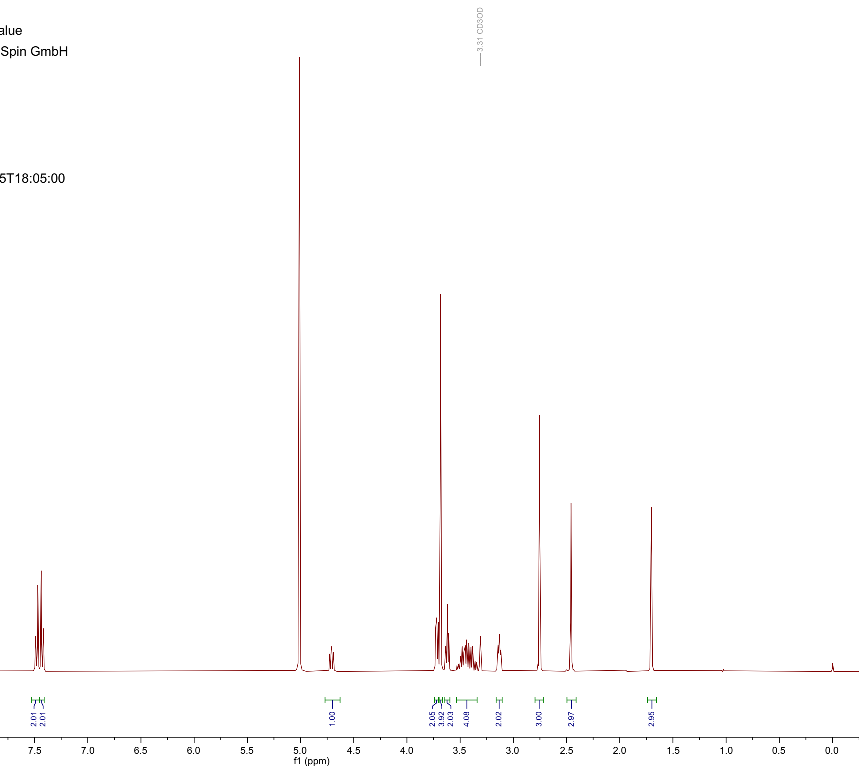

Parameter	Value
Origin	Bruker BioSpin GmbH
Solvent	MeOD
Temperature	296.2
Pulse Sequence	zgpg30
Experiment	1D
Number of Scans	16000
Acquisition Date	2021-10-16T10:05:00
Spectrometer Frequency	100.62
Spectral Width	24038.5
Lowest Frequency	-1819.5
Nucleus	13C
Acquired Size	32768
Spectral Size	65536

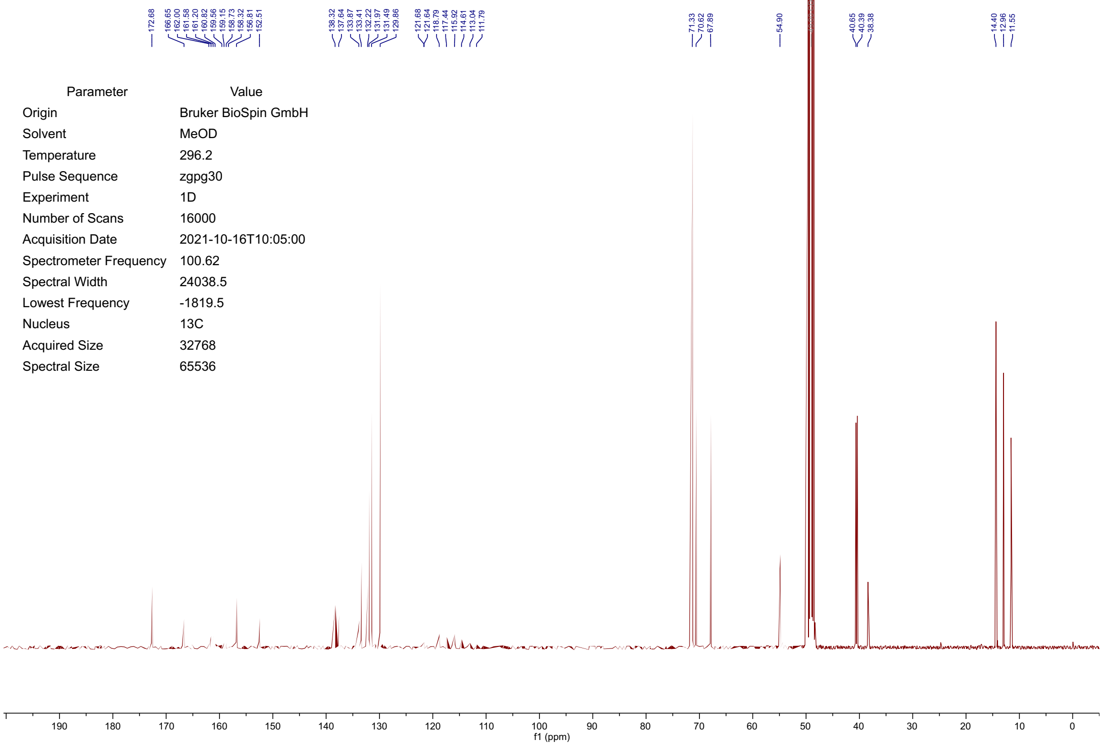

Parameter	Value
Solvent	MeOD
Temperature	298.0
Pulse Sequence	zg30
Experiment	1D
Number of Scans	48
Acquisition Date	2023-10-31T14:34:50
Spectrometer Frequency	400.13
Spectral Width	8012.8
Lowest Frequency	-1543.3
Nucleus	1H
Acquired Size	32768
Spectral Size	65536

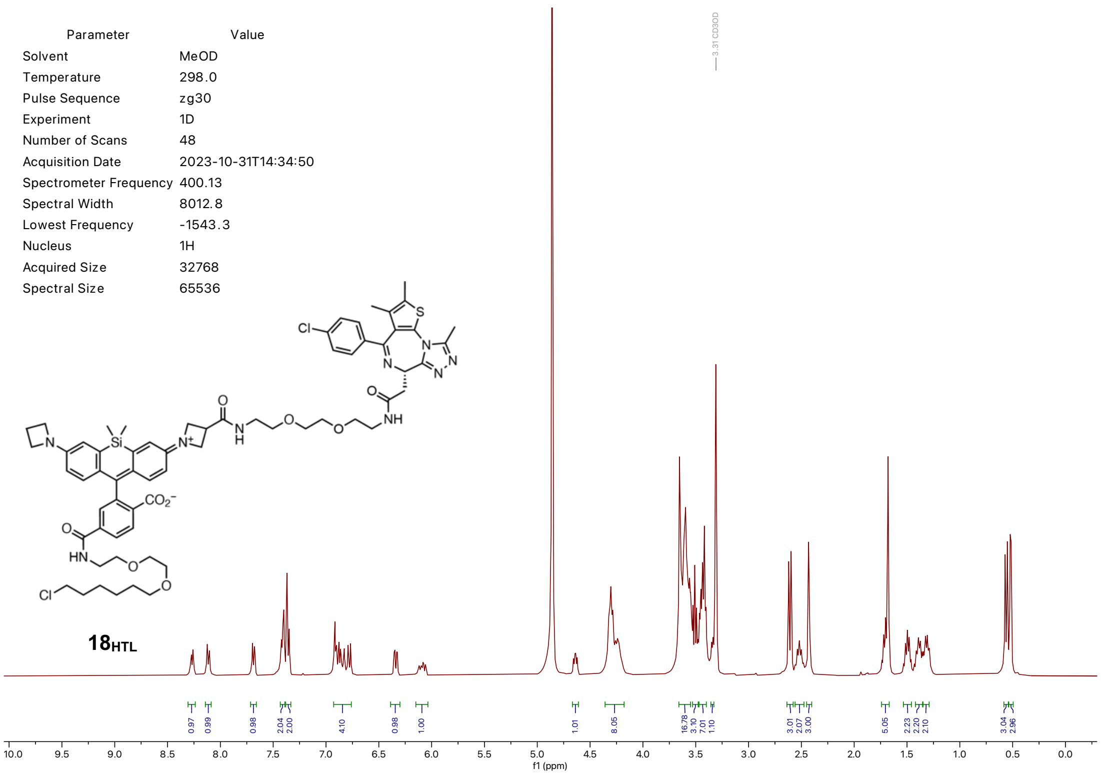

18_{HTL}

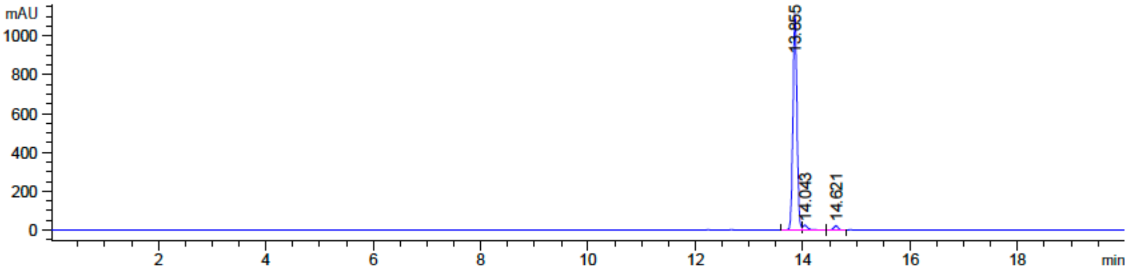

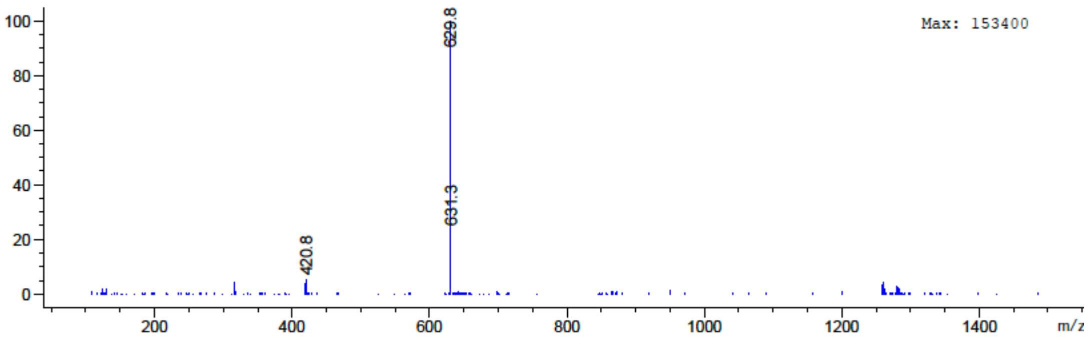

Parameter	Value
Solvent	MeOD
Temperature	298.1
Pulse Sequence	zgpg30
Experiment	1D
Number of Scans	16000
Acquisition Date	2023-10-31T14:29:19
Spectrometer Frequency	100.62
Spectral Width	24038.5
Lowest Frequency	-1817.6
Nucleus	13C
Acquired Size	32768
Spectral Size	65536

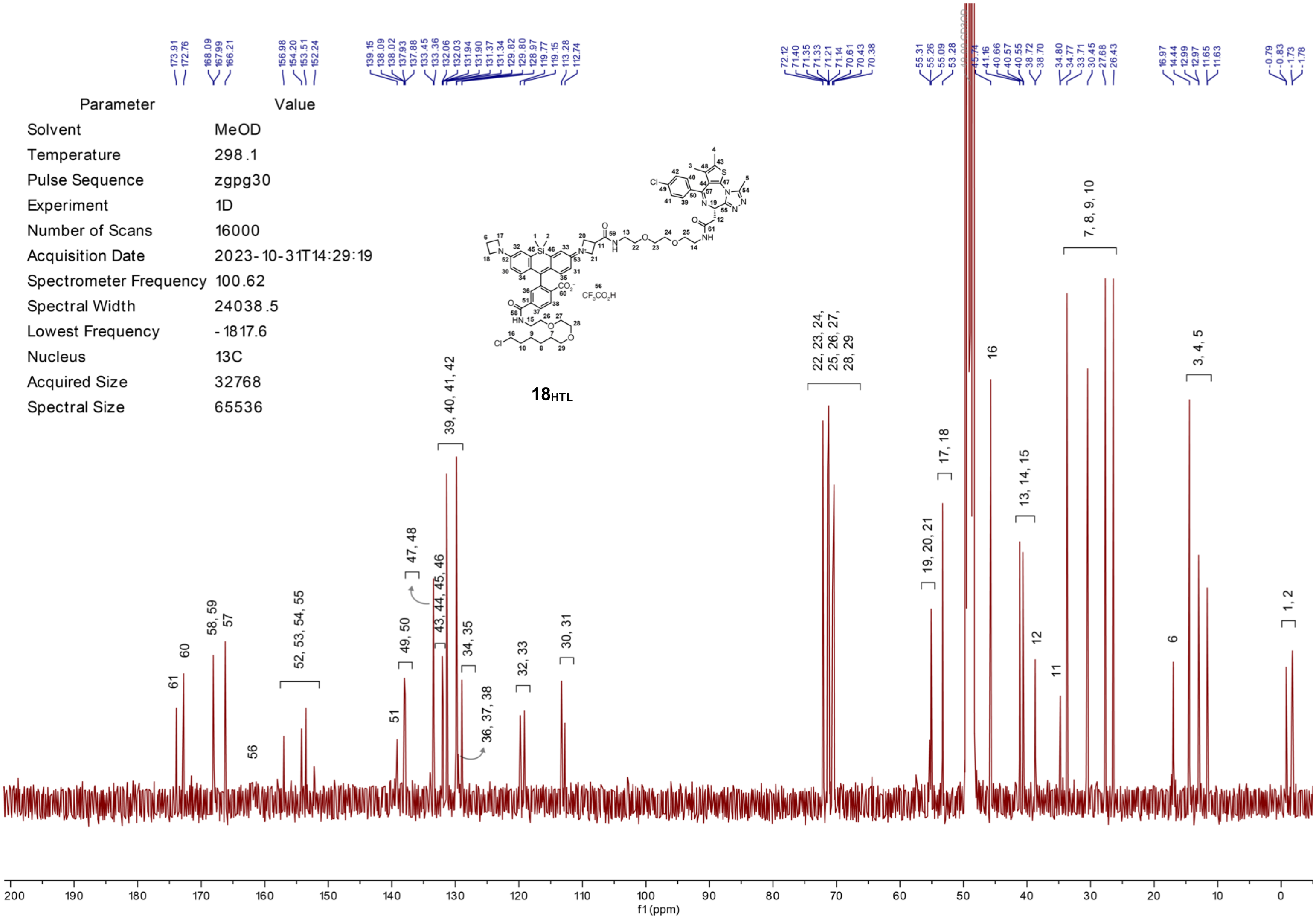

18HTL

Parameter	Value
Origin	Bruker BioSpin GmbH
Solvent	MeOD
Temperature	320.1
Pulse Sequence	zg30
Experiment	1D
Number of Scans	16
Acquisition Date	2023-06-23T22:13:36
Spectrometer Frequency	400.13
Spectral Width	8012.8
Lowest Frequency	-1542.9
Nucleus	1H
Acquired Size	32768
Spectral Size	65536

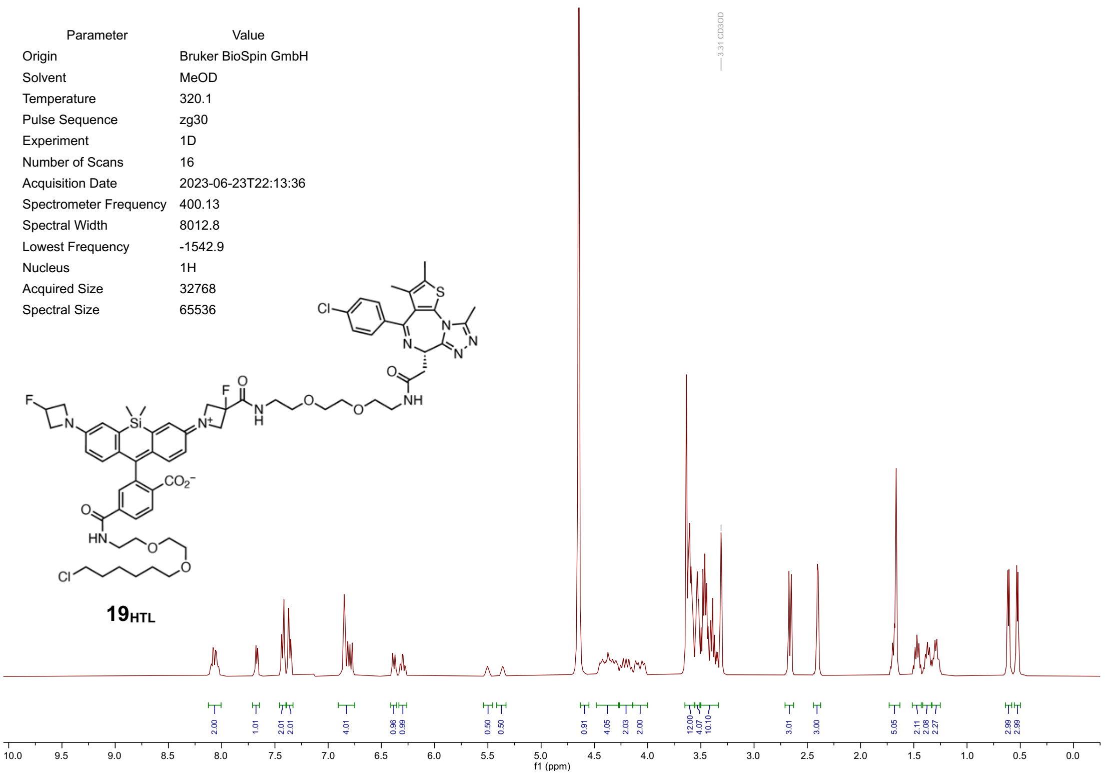

19HTL

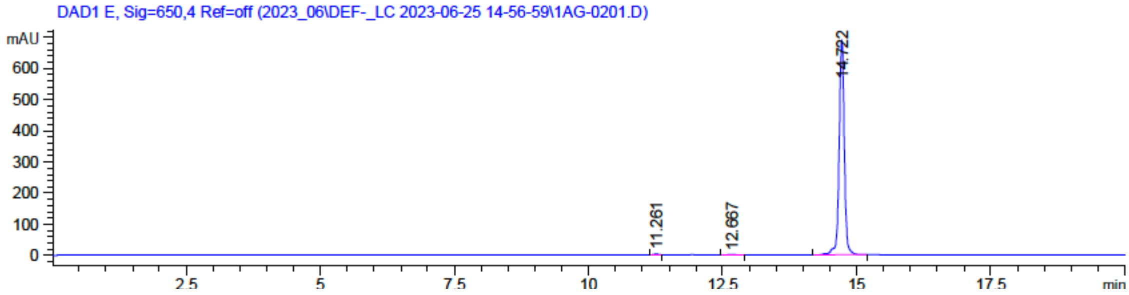

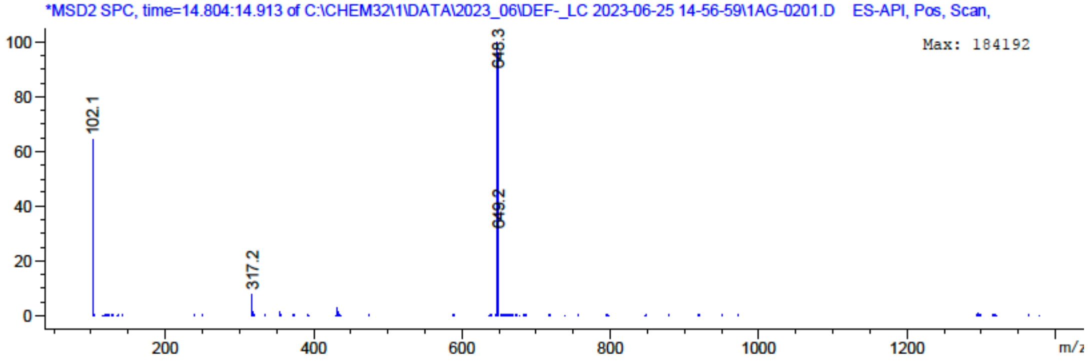

Parameter	Value
Origin	Bruker BioSpin GmbH
Solvent	MeOD
Temperature	299.9
Pulse Sequence	zgpg30
Experiment	1D
Number of Scans	20000
Acquisition Date	2023-06-25T08:53:04
Spectrometer Frequency	100.62
Spectral Width	24038.5
Lowest Frequency	-1958.0
Nucleus	13C
Acquired Size	32768
Spectral Size	65536

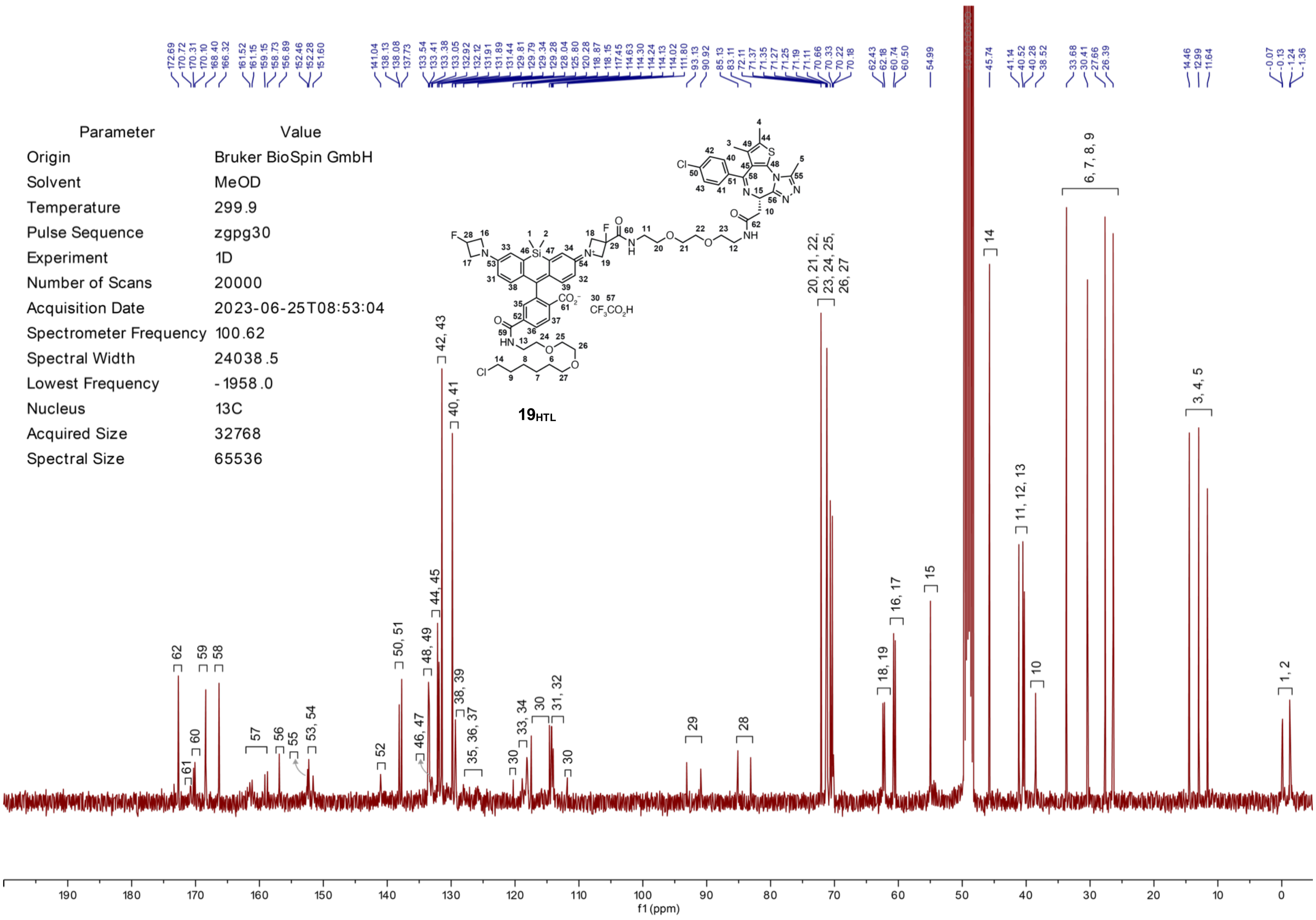

Parameter	Value
Origin	Bruker BioSpin GmbH
Solvent	MeOD
Temperature	295.5
Pulse Sequence	zg30
Experiment	1D
Number of Scans	128
Acquisition Date	2021-10-19T10:23:00
Spectrometer Frequency	400.13
Spectral Width	8012.8
Lowest Frequency	-1543.1
Nucleus	1H
Acquired Size	32768
Spectral Size	65536

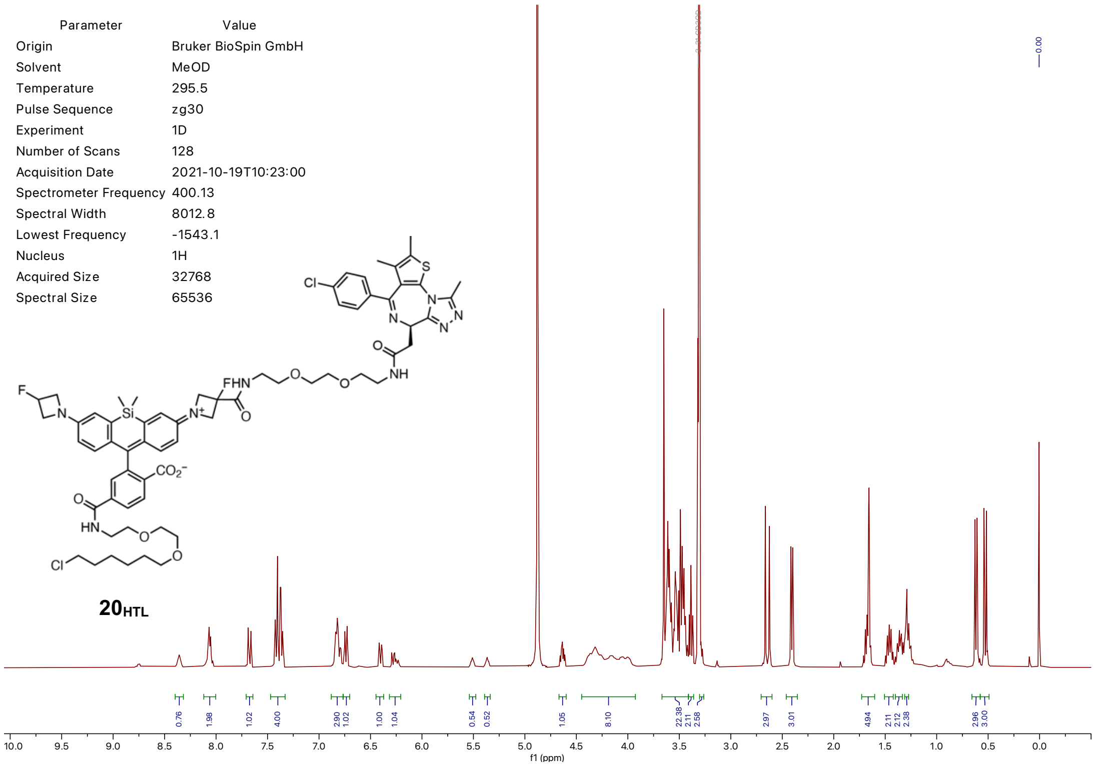

20_{HTL}

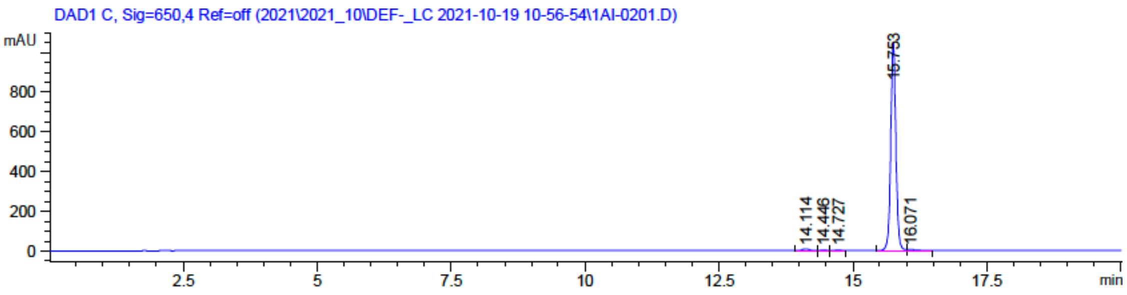

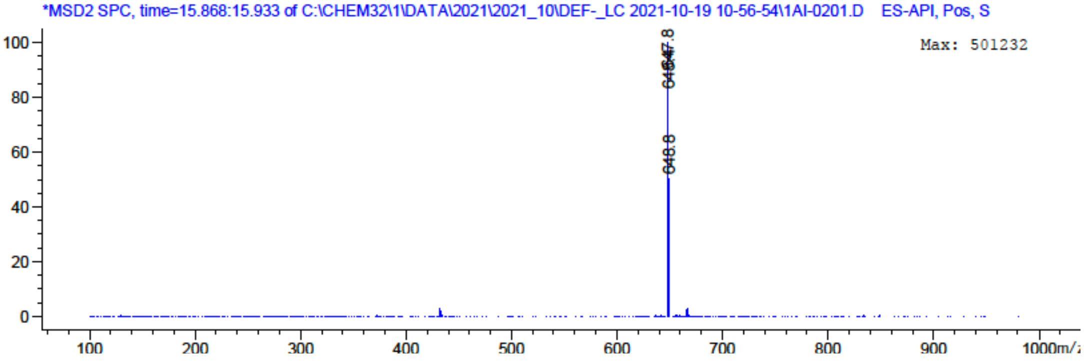

REFERENCES

(1) Encell, L. P.; Friedman Ohana, R.; Zimmerman, K.; Otto, P.; Vidugiris, G.; Wood, M. G.; Los, G. V.; McDougall, M. G.; Zimprich, C.; Karassina, N.; et al. Development of a dehalogenase-based protein fusion tag capable of rapid, selective and covalent attachment to customizable ligands. *Curr. Chem. Genomics* **2012**, *6*, 55-71.

(2) Grimm, J. B.; English, B. P.; Chen, J.; Slaughter, J. P.; Zhang, Z.; Revyakin, A.; Patel, R.; Macklin, J. J.; Normanno, D.; Singer, R. H.; et al. A general method to improve fluorophores for live-cell and single-molecule microscopy. *Nat. Methods* **2015**, *12*, 244-250.

(3) Grimm, J. B.; Muthusamy, A. K.; Liang, Y.; Brown, T. A.; Lemon, W. C.; Patel, R.; Lu, R.; Macklin, J. J.; Keller, P. J.; Ji, N.; et al. A general method to fine-tune fluorophores for live-cell and in vivo imaging. *Nat. Methods* **2017**, *14*, 987-994.

(4) Grimm, J. B.; Tkachuk, A. N.; Xie, L.; Choi, H.; Mohar, B.; Falco, N.; Schaefer, K.; Patel, R.; Zheng, Q.; Liu, Z.; et al. A general method to optimize and functionalize red-shifted rhodamine dyes. *Nat. Methods* **2020**, *17*, 815-821.

(5) Suzuki, K.; Kobayashi, A.; Kaneko, S.; Takehira, K.; Yoshihara, T.; Ishida, H.; Shiina, Y.; Oishi, S.; Tobita, S. Reevaluation of absolute luminescence quantum yields of standard solutions using a spectrometer with an integrating sphere and a back-thinned CCD detector. *Phys. Chem. Chem. Phys.* **2009**, *11*, 9850-9860.

(6) Andres, A.; Roses, M.; Rafols, C.; Bosch, E.; Espinosa, S.; Segarra, V.; Huerta, J. M. Setup and validation of shake-flask procedures for the determination of partition coefficients (logD) from low drug amounts. *Eur. J. Pharm. Sci.* **2015**, *76*, 181-191.

(7) Schönsee, C. D.; Bucheli, T. Experimental determination of octanol–water partition coefficients of selected natural toxins. *J. Chem. Eng. Data* **2020**, *65*, 1946-1953.

(8) Nemoto, Y.; De Camilli, P. Recruitment of an alternatively spliced form of synaptojanin 2 to mitochondria by the interaction with the PDZ domain of a mitochondrial outer membrane protein. *EMBO J.* **1999**, *18*, 2991-3006.

(9) Vevea, J. D.; Chapman, E. R. Acute disruption of the synaptic vesicle membrane protein synaptotagmin 1 using knockoff in mouse hippocampal neurons. *Elife* **2020**, *9*, e56469

(10) Lois, C.; Hong, E. J.; Pease, S.; Brown, E. J.; Baltimore, D. Germline transmission and tissue-specific expression of transgenes delivered by lentiviral vectors. *Science* **2002**, *295*, 868-872.

(11) Jordan, M.; Wurm, F. Transfection of adherent and suspended cells by calcium phosphate. *Methods* **2004**, *33*, 136-143.

(12) Promega Technical Note #9PIG859. *www.promega.com/resources/protocols/product-information-sheets/g/halotag-pegbiotin-ligand-protocol; accessed Feb 1, 2023.*

(13) Svendsen, S.; Zimprich, C.; McDougall, M. G.; Klaubert, D. H.; Los, G. V. Spatial separation and bidirectional trafficking of proteins using a multi-functional reporter. *BMC Cell Biol.* **2008**, *9*, 17.

(14) Schindelin, J.; Arganda-Carreras, I.; Frise, E.; Kaynig, V.; Longair, M.; Pietzsch, T.; Preibisch, S.; Rueden, C.; Saalfeld, S.; Schmid, B.; et al. Fiji: An open-source platform for biological-image analysis. *Nat. Methods* **2012**, *9*, 676-682.

(15) Corcelli, A.; Saponetti, M. S.; Zaccagnino, P.; Lopalco, P.; Mastrodonato, M.; Liquori, G. E.; Lorusso, M. Mitochondria isolated in nearly isotonic KCl buffer: Focus on cardiolipin and organelle morphology. *Biochim. Biophys. Acta* **2010**, *1798*, 681-687.

(16) Deo, C.; Sheu, S. H.; Seo, J.; Clapham, D. E.; Lavis, L. D. Isomeric tuning yields bright and targetable red Ca(2+) indicators. *J. Am. Chem. Soc.* **2019**, *141*, 13734-13738.
